# Boron‐ versus Nitrogen‐Centered Nucleophilic Reactivity of (Cyano)hydroboryl Anions: Synthesis of Cyano(hydro)organoboranes and 2‐Aza‐1,4‐diborabutatrienes

**DOI:** 10.1002/chem.202101025

**Published:** 2021-05-26

**Authors:** Annalena Gärtner, Matthäus Marek, Merle Arrowsmith, Dominic Auerhammer, Krzysztof Radacki, Dominic Prieschl, Rian D. Dewhurst, Holger Braunschweig

**Affiliations:** ^1^ Institute for Inorganic Chemistry Julius-Maximilians-Universität Würzburg Am Hubland 97074 Würzburg Germany; ^2^ Institute for Sustainable Chemistry & Catalysis with Boron Julius-Maximilians-Universität Würzburg Am Hubland 97074 Würzburg Germany

**Keywords:** boron, boryl anion, cumulene, nucleophile

## Abstract

Cyclic alkyl(amino)carbene‐stabilized (cyano)hydroboryl anions were synthesized by deprotonation of (cyano)dihydroborane precursors. While they display boron‐centered nucleophilic reactivity towards organohalides, generating fully unsymmetrically substituted cyano(hydro)organoboranes, they show cyano‐nitrogen‐centered nucleophilic reactivity towards haloboranes, resulting in the formation of hitherto unknown linear 2‐aza‐1,4‐diborabutatrienes.

## Graphical abstract

**Cyclic alkyl(amino)carbene‐stabilized** (cyano)hydroboryl anions were synthesized by deprotonation of the corresponding (cyano)dihydroborane precursors. They display boron‐centered nucleophilic reactivity towards organohalides but cyano‐nitrogen‐centered nucleophilic reactivity towards haloboranes, resulting in the formation of the first linear 2‐aza‐1,4‐diborabutatrienes.

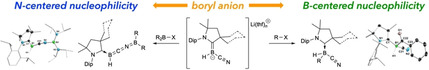

## Introduction

As anionic boron analogues of carbenes, boryl anions ([R_2_B]^−^) long remained in the realm of computational curiosities.[^1^, ^2^, ^3^] While the transient formation of [Ph_2_B]^−^ upon photolysis of [Ph_4_B]^−^ salts had been deduced from trapping reactions as early as 1967,^[4]^ these claims were later disputed.[^5^, ^6^, ^7^] Although Curran, Fensterbank, Malacria and Lacôte demonstrated in 2010 that Lewis base‐stabilized parent boryl anions [L→BH_2_]^−^ can indeed be generated in situ by the reduction of L→BH_2_I, and trapped with electrophiles, these species are too thermodynamically unstable to be isolated.[^8^, ^9^]

**Figure 1:**
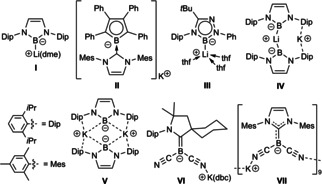
Selection of isolable boryl anions. Dme=dimethoxyethane, thf=tetrahydrofuran, dbc=dibenzo‐18‐crown‐6.

The first isolable boryl anion, compound **I** (Figure 1), was reported in 2006 by Yamashita and Nozaki, making use of the same features that help stabilize N‐heterocyclic carbenes (NHCs).^[10]^ The anionic boron center of **I** is incorporated within a five‐membered ring, electronically stabilized by the two adjacent nitrogen π donors and sterically shielded by the bulky Dip groups (Dip=2,6‐di*iso*propylphenyl), while complexation to the lithium cation provides further thermodynamic stability.[^11^, ^12^] Diazaborolyl anions such as **I** have since been used as powerful Brønsted bases, reducing agents and boron nucleophiles to access a plethora of new boron‐element bonds.[^13^, ^14^, ^15^, ^16^, ^17^, ^18^, ^19^]

In the last decade the available repertoire of stable boryl anions has been steadily increasing. In 2010 our group reduced a NHC‐stabilized bromoborole to the corresponding aromatic borolyl anion, **II**, which reacts as a boron nucleophile towards MeI.^[20]^ Taking further inspiration from NHCs, the electronic properties of which can be fine‐tuned by changes in the heterocycle, Kinjo and co‐workers designed the 1,2,4,3‐triazaborol‐3‐yl lithium complex **III**, which is capable of coupling two CO molecules to a diboraalkene and cyclizing arylisonitriles to 2‐boranylindole derivatives.^[21]^ The reactivity of **I** was further increased by synthesizing the mixed lithium‐potassium salt of the diazaborolyl anion dimer, **IV**, which is capable of deprotonating benzene,^[22]^ or the dimeric potassium salt **V**, in which both anionic boron centers are “naked” and promote facile C−H activation at a pendant Dip‐*iso*propyl group.^[23]^

While boryl anions **I**–**V** are accessed by reduction of haloboron precursors, Bertrand and co‐workers showed that the B−H bond of a cyclic alkyl(amino)carbene (CAAC)‐stabilized (dicyano)hydroborane undergoes an “umpolung” due to the electron‐withdrawing nature of the cyano ligands, which enables the synthesis of the corresponding boryl anion **VI** by deprotonation.^[24]^ The negative charge at boron in **VI** is further stabilized by π backdonation to the π‐acidic CAAC ligand. Using the same umpolung principle, Finze and co‐workers deprotonated [BH(CN)_3_]^−^ to the corresponding dianion [B(CN)_3_]^2−^,^[25]^ while Hörner and Frank synthesized stable NHC analogues of **VI**, such as the nonameric potassium salt **VII**.^[26]^ The latter was shown to react as a boron‐centered nucleophile with a variety of organic, main group and transition metal electrophiles.

**Scheme 1:**
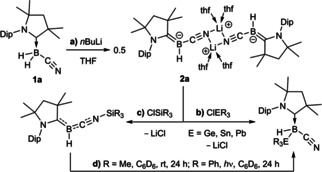
Synthesis of boryl anion **2 a** and its reactivity with group 14 electrophiles.

Our group has shown that a single cyano ligand at boron suffices to induce a B−H bond umpolung, enabling access to the (cyano)hydroboryl anion dimer **2 a** by deprotonation of the corresponding (CAAC^Me^)BH_2_(CN) precursor **1 a** (Scheme 1a, CAAC^Me^=1‐(2,6‐di*iso*propylphenyl)‐3,3,5,5‐tetramethyl‐pyrrolidin‐2‐ylidene).^[27]^

While **2 a** reacted as a soft boron‐centered nucleophile towards soft heavier triorganotetrel chlorides (Scheme 1b), the reaction with triorganosilyl chlorides occurred at the hard terminal cyano‐nitrogen, generating a silylisonitrile‐stabilized borylene (Scheme 1c), which in solution undergoes subsequent silyl migration to boron (Scheme 1d).

The divergent reactivity of **2 a**, both as a soft boron‐ and as a hard nitrogen‐centered nucleophile, prompted us to undertake further investigations into the reactivity of (cyano)hydroboryl anions with electrophiles. In this contribution we present the synthesis of monomeric and dimeric CAAC‐stabilized (cyano)hydroboryl anions and show their divergent reactivity towards a wide range of organic electrophiles and haloboranes, leading to fully unsymmetrically‐substituted cyano(hydro)boranes and the first examples of 2‐aza‐1,4‐diborabutatrienes (i. e. LHB=C=N=BR_2_), respectively.

## Results and Discussion

### Synthesis of boryl anion precursors

**Scheme 2:**
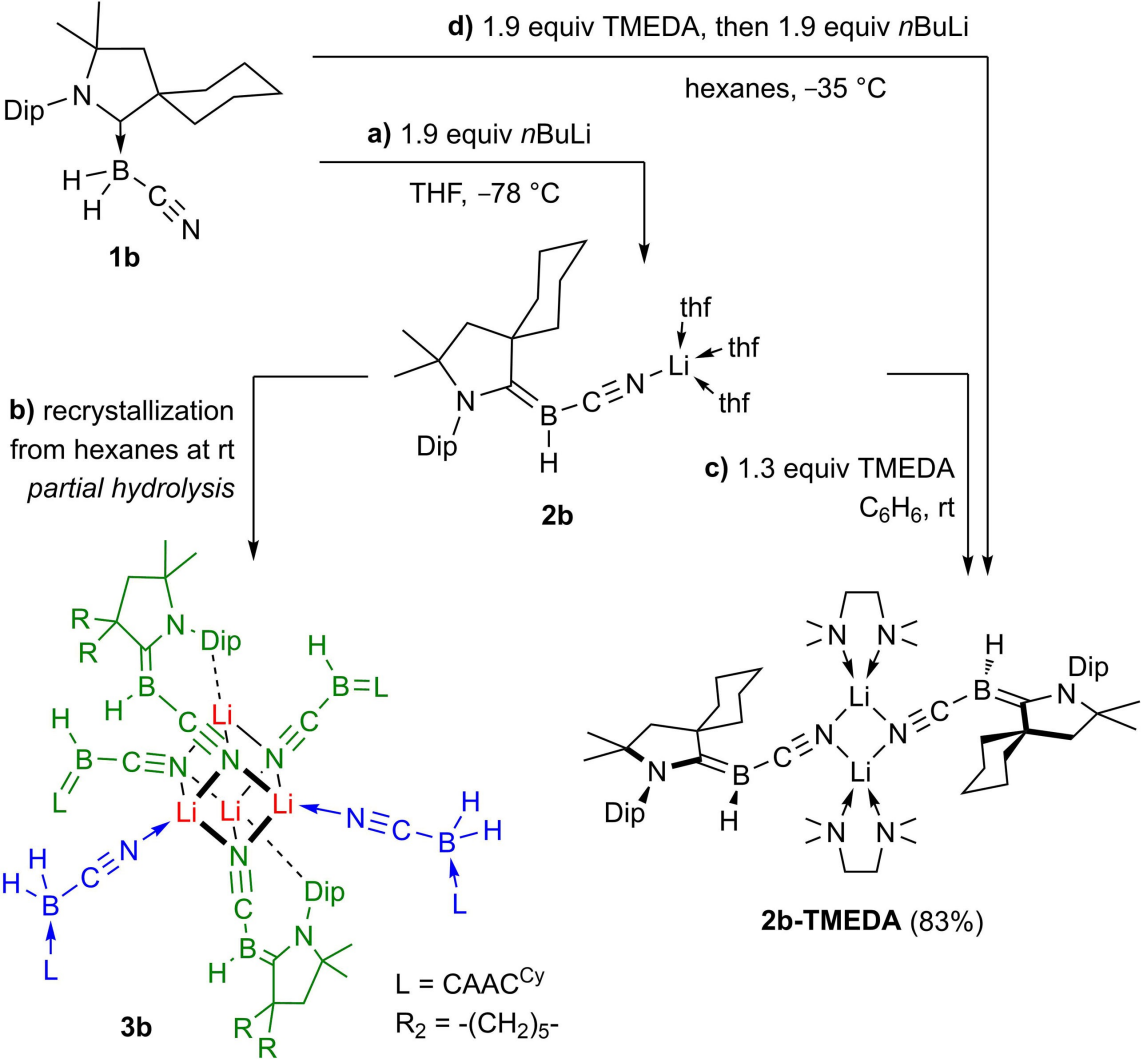
Synthesis of boryl anions **2 b** and **2 b‐TMEDA**, and partial hydrolysis of **2 b** to **3 b** (boryl anion units shown in green, borane units in blue). Isolated yields in brackets. No yield determined for the hydrolysis product **3 b**. TMEDA = tetramethylethylenediamine.

**Figure 2:**
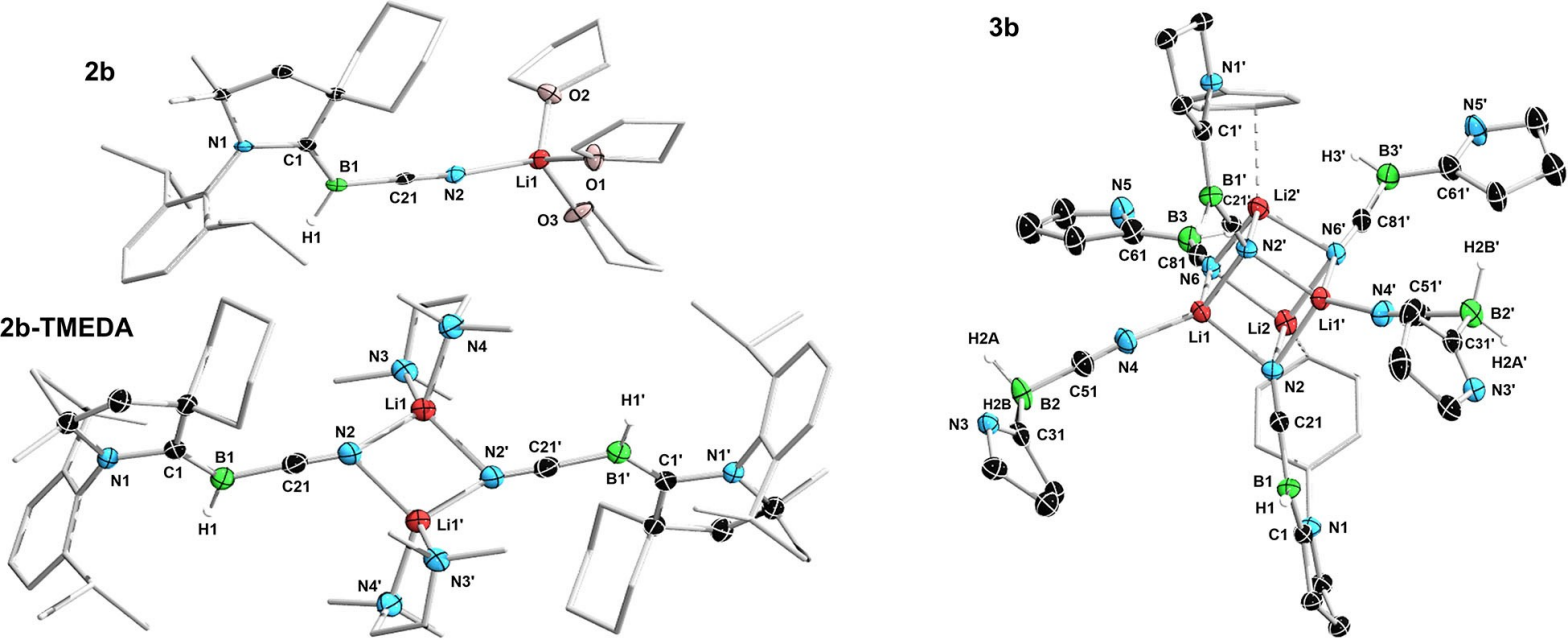
Crystallographically derived molecular structures of **2 b**, **2 b‐TMEDA** and **3 b**. Atomic displacement ellipsoids are set at 50 % probability. Ellipsoids of carbon atoms of the ligand periphery and hydrogen atoms omitted for clarity, except for boron‐bound hydrides. For further clarity Me and Dip groups of **3 b** were also omitted, except for Dip groups at N1/N1′ (*i*Pr groups omitted) to show the π interaction with Li2/Li2′. Selected bond lengths (Å) and angles (°) for **2 b**: N1−C1 1.417(4), C1−B1 1.450(5), B1−C21 1.539(5), C21−N2 1.158(3), N2−Li1 1.989(6), B1−C21−N2 176.8(3), C21−N2−Li 173.9(3), Σ(∠B)=359.8(2); for **2 b‐TMEDA**: N1−C1 1.3973(16), C1−B1 1.465(2), B1−C21 1.518(2), C21−N2 1.1451(18), N2−Li1 2.022(3), N2−Li1′ 2.086(3), B1−C21−N2 175.73(15), Li1−N2−Li1′ 83.83(10), N2−Li1−N2′ 96.16(10), Σ(∠B)=359.8(7); for **3 b**: C1−B1 1.4684(19), B1−C21 1.5137(19), C21−N2 1.1718(17), C31−B2 1.615(2), B2−C51 1.592(2), C51−N4 1.1427(19), N4−Li1 1.950(3), C61−B3 1.470(2), B3−C81 1.506(2), C81−N6 1.1863(18), Li1−N2 2.116(2), Li1−N6 2.076(3), Li2−N2 2.032(3), Li2−N6 2.077(3), Li1−N2′ 2.126(3), Li2−N6′ 2.137(3), Li2⋅⋅⋅Ar 2.289(3), Σ(∠B1)=360.0(6), Σ(∠B3)=359.9(7), Σ(∠Li1_cube_)=284.57(10), Σ(∠Li2 _cube_)=287.68(11).

With the aim of obtaining a monomeric analogue of **2 a**, the more sterically demanding CAAC^Cy^ ligand (CAAC^Cy^=2‐(2,6‐di*iso*propylphenyl)‐3,3‐dimethyl‐2‐azaspiro[4.5]decan‐1‐ylidene) was employed to synthesize the (cyano)dihydroborane precursor **1 b**. The deprotonation of **1 b** with *n*BuLi in THF yielded the boryl anion **2 b** (Scheme 2a), which displays an ^11^B NMR doublet at −13.9 ppm, nearly identical to **2 a** (δ_11B_=−10.8 ppm).^[27]^ Due to its extremely high solubility in nonpolar hydrocarbon solvents only a few crystals of **2 b** could be obtained, which confirmed the monomeric nature of the species (Figure 2).

Unlike in **2 a**, the lithium cation in **2 b** is terminally bound to the cyano substituent and stabilized by three THF molecules. The boron center is trigonal planar (Σ(∠B)=359.8(2)°) while the B1−C1 bond length of 1.450(5) Å is similar to that of **2 a** (1.447(3) Å)^[27]^ and denotes a B=C double bond, indicating strong π backdonation from the boryl anion lone pair into the empty p orbital at the CAAC ligand. Due to the high Brønsted basicity of the borylene all attempts to recrystallize **2 b** in higher yields from hexanes resulted in partial hydrolysis back to **1 b** and crystallization of the cubane‐type cluster **3 b** (Scheme 2b, Figure 2). Four [(CAAC^Cy^)BH(CN)Li] units generate the central Li_4_N_4_ cube of *C*_2*v*_ symmetry, the opposing lithium apices of which are stabilized by two (CAAC^Cy^)BH_2_(CN) units coordinating as nitrogen donors, and two π interactions with adjacent Dip substituents, respectively. The structure of **3 b** is reminiscent of that of [(PhC=N)Li(NC_5_H_5_)]_4_, which also crystallizes as a tetramer with a cubic Li_4_N_4_ core and pyridine‐stabilized lithium apices.^[28]^

In order to isolate **2 b** in high yields and analytical purity its tetramethylethylenediamine (TMEDA) analogue **2 b‐TMEDA** was synthesized by the addition of TMEDA to crude **2 b** (Scheme 2c), or alternatively by deprotonating **1 b** in hexanes at −35 °C in the presence of TMEDA (Scheme 1d). **2 b‐TMEDA** (δ_11B_=−12.9 ppm, d, ^1^ *J*_B‐H_=98.3 Hz) was isolated as a yellow crystalline solid in 62 % yield. Its solid‐state structure (Figure 2) is dimeric, similar to that of **2 a**. The main difference with **2 a** is that the borylene plane in **2 b‐TMEDA** is not coplanar with the central Li_2_N_2_ unit but rotated by ca. 70°, presumably due to steric repulsion between the TMEDA ligands and the CAAC‐cyclohexyl moiety.

### Reactions of 2 a and 2 b as boron‐centered nucleophiles

**Scheme 3:**
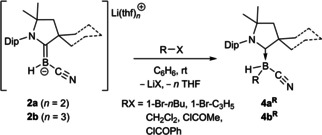
Reactivity of **2 a** and **2 b** towards organohalide electrophiles (**a**: with CAAC^Me^; **b**: with CAAC^Cy^). Isolated yields: **4 a^R^**, R=COMe (83 %), COPh (79 %), CH_2_Cl (72 %), *n*Bu (61 %), C_3_H_5_ (74 %); **4 b^R^**, R=COMe (79 %), COPh (73 %), CH_2_Cl (71 %), C_3_H_5_ (87 %).

Similarly to **I**,[^10^, ^17^, ^18^, ^29^] the reactions of the boryl anions **2 a** and **2 b** towards organohalides (RX=1‐bromobutane, allyl bromide, dichloromethane, acetyl chloride, benzoyl chloride) at room temperature resulted in the clean formation of the corresponding colorless cyano(hydro)organoboranes **4 a^R^** and **4 b^R^**, respectively, by salt elimination (Scheme 3). The ^11^B NMR shifts of each **4 a^R^**/**4 b^R^** pair are nearly identical (Δδ_11B_≈1.5 ppm) and range from −23.6 ppm for R=COMe to −25.1 ppm for R=Br. This boron‐centered reactivity reflects that of **2 a** with heavier group 14 triorganochlorides (Scheme 1b).^[27]^

It is noteworthy that unlike **2 a** and **2 b**, the diazaborolyl anion **I** reacts with 1‐bromobutane to provide the corresponding bromoborane rather than the butylborane.^[17]^ Marder and Lin showed that the preference of boryl anion **I** for halogen abstraction from RX over nucleophilic attack at the halide‐bound carbon atom increases for the heavier halides, and can be promoted by the stability of the R^−^ fragment in the transition state of the halogen abstraction step.^[30]^

**Scheme 4:**
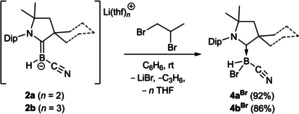
Reactivity of **2 a** and **2 b** towards 1,2‐dibromopropane. Isolated yields in brackets.

The reaction between **2 a** or **2 b** with 1,2‐dibromopropane in benzene at room temperature was accompanied by rapid gas evolution as the reaction mixture became colorless. In C_6_D_6_ the ^1^H NMR spectra of the two reaction mixtures showed the formation of propene as a by‐product (δ_1H_=5.71, 4.99 and 4.93 ppm for the alkene protons), while the ^11^B NMR spectra showed a single boron‐containing product with a doublet around −25 ppm (^1^ *J*_B‐H_≈100 Hz), identified by X‐ray structural analyses as the CAAC‐stabilized bromo(cyano)hydroboranes **4 a^Br^** and **4 b^Br^**, respectively (Scheme 4). In this case the entropically favored elimination of propene is likely to be the driving force of the bromine abstraction versus the nucleophilic attack at carbon.

**Figure 3:**
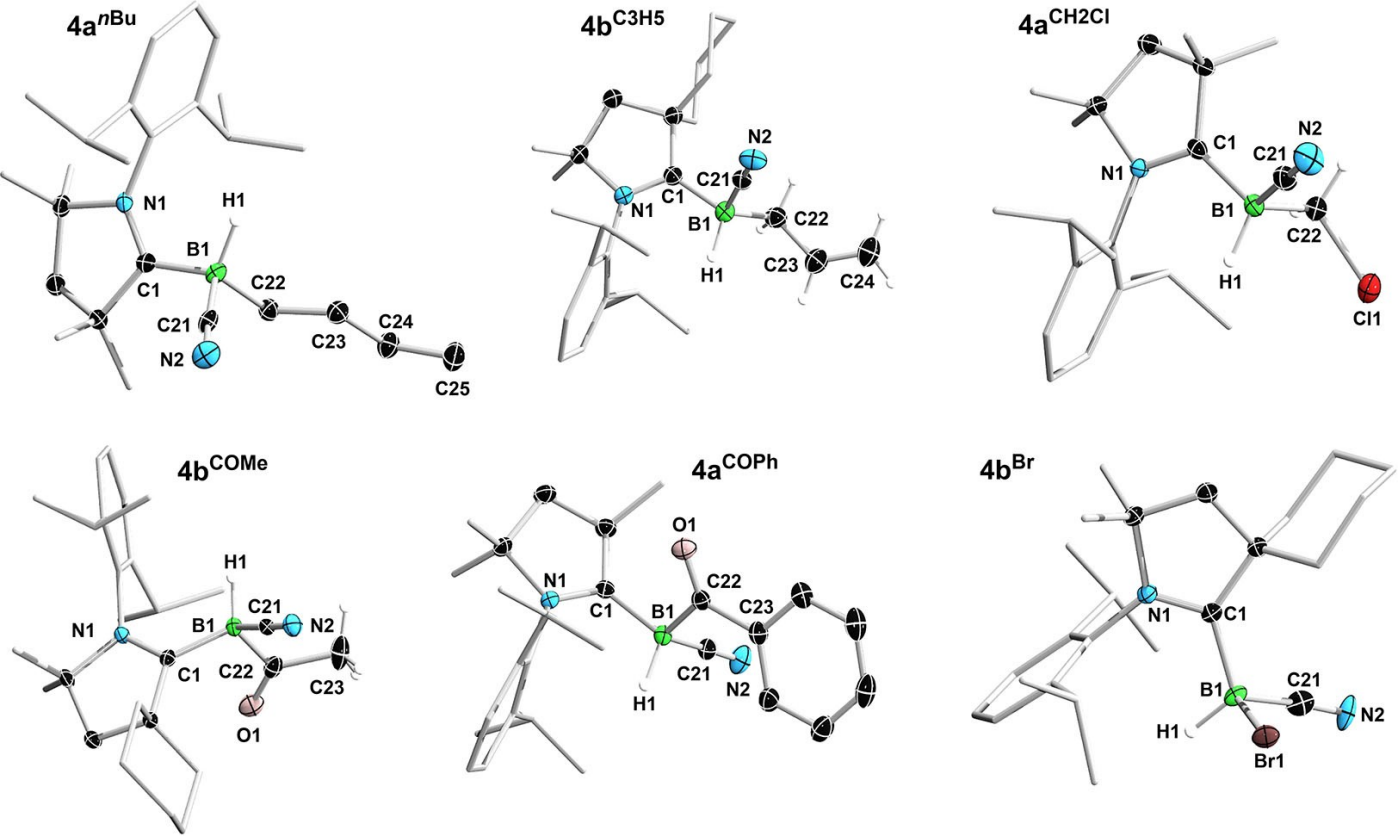
Crystallographically derived molecular structures of **4 a** ^***n*****Bu**^, **4 b^C3H5^**, **4 a^CH2Cl^**, **4 b^COMe^**, **4 a^COPh^** and **4 b^Br^**. Atomic displacement ellipsoids are set at 50 % probability. Ellipsoids of carbon atoms of the ligand periphery and hydrogen atoms omitted for clarity, except for boron‐bound hydrides.

All **4 a/b^R^** boranes crystallized as colorless solids and were characterized by X‐ray crystallographic analyses (Figure 3 and Figures S60–S64). In all cases the B1−C1 bond length is ca. 1.62 Å, slightly longer than in the precursors **1 a** (1.608(3) Å) and **1 b** (1.597(3) Å), typical for a CAAC→borane donor interaction, while the C1−N1 bond length of ca. 1.30 Å corresponds to a double bond. Furthermore, the substituents at boron are always arranged so that the N1−C1−B1−H1 torsion angle tends towards 0° (from ca. 0.9° in **4 a** ^***n*****Bu**^ to ca. 20.7° in **4 a^COPh^**), in order to minimize steric repulsion between the Dip substituent and the CN and R substituents. Overall the syntheses presented in Schemes 2 and 3 represent facile routes to fully unsymmetrically substituted boranes, which would otherwise be difficult to access.

### Reactions of 2 a and 2 b as cyano‐nitrogen‐centered nucleophiles

**Scheme 5:**
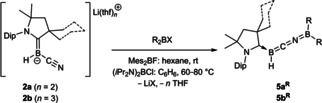
Reactivity of **2 a** and **2 b** towards haloboranes. Mes=2,4,6‐Me_3_C_6_H_2_. Isolated yields: **5 a^R^**, R=Mes (89 %), N*i*Pr_2_ (76 %); **5 b^R^**, R=Mes (74 %), N*i*Pr_2_ (68 %).

The reactivity of **2 a** and **2 b** towards haloboranes (R_2_BX) proceeded rather differently. Instead of turning colorless as in the reactions with organohalides the reaction mixtures turned dark red or bright orange for Mes_2_BF (Mes=2,4,6‐Me_3_C_6_H_2_) or (*i*Pr_2_N)_2_BCl, respectively. The ^11^B NMR spectra of the reaction products, compounds **5 a/b^R^**, showed a broad resonance around 36 ppm for **5 a/b^Mes^** and 22 ppm for **5 a/b^N*i*Pr2^**, upfield‐shifted from the precursors Mes_2_BF (δ_11B_=53 ppm)^[31]^ and (*i*Pr_2_N)_2_BCl (δ_11B_=34 ppm),^[32]^ as well as a B*H* doublet around −9 ppm for **5 a/b^Mes^** and −14 ppm **5 a/b^N*i*Pr2^**, in a similar range to those of precursors **2 a** and **2 b** (δ_11B_=−10.8 and −12.9 ppm, respectively). These data suggested nucleophilic attack at the borane by the terminal cyano‐nitrogen donor rather than by the boryl anion (Scheme 5), in analogy to the reaction between **2 a** and triorganosilyl chlorides (Scheme 1c).^[27]^

**Figure 4:**
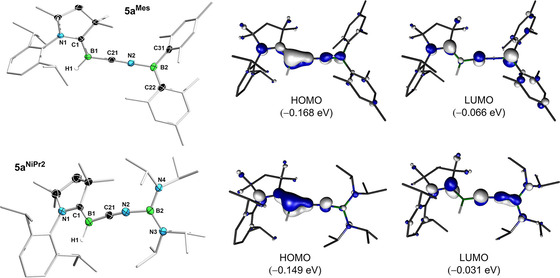
Left: Crystallographically‐derived molecular structures of **5a^Mes^** and **5a^N*i*Pr2^**. Atomic displacement ellipsoids are set at 50 % probability. Ellipsoids of carbon atoms of the ligand periphery and hydrogen atoms omitted for clarity, except for boron‐bound hydrides. Selected bond lengths (Å) and angles (°) for **5 a^Mes^**: N1−C1 1.3382(13), C1−B1 1.5117(17), B1−C21 1.4574(15), C21−N2 1.158(3), N2−B2 1.4107(15), B1−C21−N2 174.05(11), C21−N2−B2 169.71(11), Σ(∠B1)=359.3(5), Σ(∠B2)=359.97(10); for **5 a^N*i*Pr2^**: N1−C1 1.347(9), C1−B1 1.449(5), B1−C21 1.4801(16), C21−N2 1.1814(14), N2−B2 1.4338(14), B2−N3 1.4158(14), B2−N4 1.4263(14), B1−C21−N2 174.56(11), C21‐N2‐B2 156.36(10), Σ(∠B1)=359.68(5), Σ(∠B2)=360.00(9). Right: Canonical Kohn−Sham molecular orbitals of **5 a^Mes^** and **5 a^N*i*Pr2^** at the B3LYP/def2‐SVP level of theory.

This was confirmed by X‐ray structural analyses (Figure 4, Figures S65 and S66), which showed that the geometry around B1 remains trigonal planar (Σ(∠B1) ≈360°) and the BR_2_ moiety coordinates at the cyano‐nitrogen N2. The B1−C21−N2−B2 moiety presents a near‐linear arrangement with a B1‐C21‐N2 angle of ca. 174° and a C21−N2−B2 angle of ca. 170° for **5 a/b^Mes^** and ca. 160° for **5 a/b^N*i*Pr2^**. In the **5 a/b^Mes^** derivatives the B1−C21, C21−N2 and N2−B2 distances (ca. 1.45, 1.17 and 1.41 Å, respectively) are all within the range of double bonds,[^33^, ^34^] while the C1−B1 and N1−C1 bond lengths (ca. 1.51 and 1.34 Å, respectively) suggest only a small amount of π backbonding from the electron‐rich boron to the CAAC ligand. In the **5 a/b^N*i*Pr2^** derivatives the electronic structure is slightly different: due to partial π backbonding from the amino substituents to B2 (B2‐N3/4 ca. 1.43 Å) the B1−C21, C21−N2 and N2−B2 distances (ca. 1.48, 1.18 and 1.45 Å, respectively) are slightly elongated compared to **5 a/b^Mes^**. Moreover, the C1−B1 and N1−C1 bond lengths (ca. 1.47 and 1.36 Å, respectively) also indicate partial double bonds, suggesting that π electron density in **5 a/b^N*i*Pr2^** is delocalized over the entire [N1−C1−B1−C21−N2−B2−N3/4] framework. The preference for the addition of the boron electrophiles at the cyano‐nitrogen rather than at the borylene center is likely owed to the formation of a strong B−N multiple bond rather than a comparatively weak B−B single bond.

The UV‐vis spectra of **5 a^Mes^** and **5 b^Mes^** in hexane showed absorption maxima at 516 and 520 nm, respectively, accounting for their red color, as well as a secondary absorption band around 380 nm. In contrast, the major absorption bands of **5 a^N*i*Pr2^** (423 nm) and **5 b^N*i*Pr2^** (429 nm) are blueshifted by ca. 100 nm compared to those of **5 a^Mes^** and **5 b^Mes^**, accounting for their orange color, and overlap with strong secondary absorptions around 400 nm.

Density functional theory (DFT) calculations on **5 a^Mes^** and **5 a^N*i*Pr2^** performed at the B3LYP/def2‐SVP^[35]^ and PBE0‐D3/def2‐SVP[^36^, ^37^] levels of theory (see Supporting Information for details) showed that in both compounds the canonical Kohn‐Sham HOMO extends over the [N1−B1−C21−N2−B2(‐N3/4)] π systems, with a strong π‐bonding character between B1 and C21, and to a lesser degree B1 and C1 (Figure 4), as suggested by the X‐ray crystallographic data. Nodal planes are found in the N1‐C1 and C21‐N2 bond regions. Additionally, the **5 a^N*i*Pr2^** derivative shows small contributions from the lone pairs at N3 and N4, with nodal planes at B2−N3 and B2−N4. Furthermore, bond orders calculated using natural resonance theory (NRT)^[38]^ analysis (see Figure S67 in the Supporting Information) suggest a better delocalization of the [N1−C1−B1−C21−N2] π system in **5 a^N*i*Pr2^** compared to **5 a^Mes^**, as deduced from the crystallographic data. **5 a/b^R^** may thus be regarded as the first examples of 2‐aza‐1,4‐diborabutatrienes. Other reported azaborabutatrienes include a 1‐aza‐2‐borabutatriene rhodium complex generated by borylene transfer from a molybdenum aminoborylene complex to a rhodium vinylidene,^[39]^ as well as a couple of aminoborylacetylenes Et_2_N−C≡C−BY_2_ (Y=Mes, NMe_2_),[^40^, ^41^] which show significant contribution from their 1‐aza‐4‐borabutatriene resonance forms, Et_2_N=C=C=BY_2_.

## Conclusion

We have shown that CAAC‐stabilized (cyano)hydroboryl anions may be isolated as either dimeric or monomeric lithium THF adducts, depending on the sterics of the CAAC ligand. The monomeric species, however, is extremely moisture‐sensitive and is best generated in situ for further reactivity. The prepared [(CAAC)BH(CN)]^−^ anions acts as boron‐centered nucleophiles towards the majority of organohalides, including alkyl, haloalkyl, and allyl halides as well as acid chlorides, resulting in the formation of fully unsymmetrically substituted cyano(hydro)organoboranes. This reaction constitutes a very promising late‐stage method to elaborate the family of boranes with a wide range of organic electrophiles, compounds that would otherwise be difficult to prepare. In contrast, the reaction with 1,2‐dibromopropane leads to bromine abstraction and elimination of propene gas, which is likely the driving force of this reaction. The reactions of [(CAAC)BH(CN)]^−^ with haloboranes result in nucleophilic attack by the terminal cyano nitrogen atom and the formation of a linear B=C=N=B chain. X‐ray crystallographic and DFT analyses of these orange/red compounds show a significant contribution of the cumulenic B=C=N=B resonance form, making these the first examples of azadiborabutatrienes.

## Experimental Section

**Crystallographic data**: Deposition numbers 2065319, 2065320, 2065321, 2065322, 2065323, 2065324, 2065325, 2065326, 2065327, 2065328, 2065329, 2065330, 2065331, 2065332, 2065333, 2065334, 2065335, 2065336, and 2065337 contain the supplementary crystallographic data for this paper. These data are provided free of charge by the joint Cambridge Crystallographic Data Centre and Fachinformationszentrum Karlsruhe Access Structures service.

## Conflict of interest

The authors declare no conflict of interest.

## Supporting information

As a service to our authors and readers, this journal provides supporting information supplied by the authors. Such materials are peer reviewed and may be re‐organized for online delivery, but are not copy‐edited or typeset. Technical support issues arising from supporting information (other than missing files) should be addressed to the authors.

Supplementary
