# Supplementary material for: Boron‐ versus Nitrogen‐Centered Nucleophilic Reactivity of (Cyano)hydroboryl Anions: Synthesis of Cyano(hydro)organoboranes and 2‐Aza‐1,4‐diborabutatrienes
Source: Chemistry. 2021 May 26;27(37):9694–9. doi: 10.1002/chem.202101025 (PMC8362087; doi:10.1002/chem.202101025)
Supplement: Supplementary file 1 — Supplementary [file CHEM-27-9694-s001.pdf]

Chemistry–A European Journal

Supporting Information

Boron- versus Nitrogen-Centered Nucleophilic Reactivity of (Cyano)hydroboryl Anions: Synthesis of Cyano(hydro) organoboranes and 2-Aza-1,4-diborabutatrienes

Annalena Gärtner, Matthäus Marek, Merle Arrowsmith, Dominic Auerhammer, Krzysztof Radacki, Dominic Prieschl, Rian D. Dewhurst, and Holger Braunschweig*

Table of contents

Methods and materials 2

Synthetic procedures 3

NMR spectra of isolated compounds 16

NMR spectrum of the reaction of **2a** with 1,2-dibromopropane 71

UV-vis spectra 72

X-ray crystallographic data 74

Computational details 84

References 90

Methods and materials

All manipulations were performed either under an atmosphere of dry argon or in vacuo using standard Schlenk line or glovebox techniques. Deuterated solvents were dried over molecular sieves and degassed by three freeze-pump-thaw cycles prior to use. All other solvents were distilled and degassed from appropriate drying agents. Solvents were stored under argon over activated 4 Å molecular sieves. NMR spectra were acquired on a Bruker Avance 500 NMR spectrometer. Chemical shifts (δ) are provided in ppm and internally referenced to the carbon nuclei ($^{13}C\{^{1}H\}$) or residual protons (^{1}H) of the solvent. Heteronuclei NMR spectra are referenced to external standards (^{11}B: $BF_3{\cdot}OEt_2$). UV-vis spectra were measured on a JASCO V-660 UV-vis spectrometer inside a glovebox. High-resolution mass spectrometry (HRMS) data were obtained from a Thermo Scientific Exactive Plus spectrometer.

Solvents and reagents were purchased from Sigma-Aldrich or Alfa Aesar. $CAAC^{Me}$ (1-(2,6-diisopropylphenyl)-3,3,5,5-tetramethylpyrrolidin-2-ylidene),[1] $CAAC^{Cy}$ (2-(2,6-diisopropylphenyl)-3,3-dimethyl-2-azaspiro[4.5]decan-1-ylidene),[1] $[(CAAC^{Me})BH(CN)Li(thf)_2]_2$ **2a**,[2] Mes_2BF (Mes = 2,4,6-$Me_3C_6H_2$)[3] and $(i\mathrm{Pr}_2N)_2BCl$[4] were synthesized using literature procedures.

Synthetic procedures

[(CAAC^{Cy})BH$_2$(CN)], 1b

(Me_3N)BH_2(CN) (1.10 g, 11.3 mmol) was dissolved in benzene (50 mL) and added to a freshly prepared solution of CAAC^{Cy} (3.54 g, 10.9 mmol) dissolved in benzene (200 mL). The resulting green reaction mixture was stirred for 4 days under reflux. All volatiles were removed *in vacuo* and the green solid was washed with three portions of hexane (20 mL each). After drying *in vacuo* residual (Me_3N)BH_2(CN) was removed by sublimation at 70 °C. Compound **1b** was obtained as a colorless solid (3.32 g, 9.11 mmol, 84%). $^{1}H\{^{11}B\}$ NMR (500 MHz, C_6D_6, 297 K): δ = 7.12 (t, ^{3}J = 7.8 Hz, 1H, *p*-Ar*H*), 6.98 (d, ^{3}J = 7.8 Hz, 2H, *m*-Ar*H*), 2.87 (dt, ^{3}J = 13.3 Hz, ^{3}J = 3.5 Hz, 2H, Cy-C*H*$_2$), 2.49 (sept, ^{3}J = 6.7 Hz, 2H, *i*Pr-C*H*), 1.86 (s, 2H, B*H*), 1.58–1.49 (m, 3H, Cy-C*H*$_2$), 1.47 (s, 2H, C*H*$_2$), 1.43–1.38 (m, 1H, Cy-C*H*$_2$), 1.37–1.33 (m, 2H, Cy-C*H*$_2$), 1.31 (d, ^{2}J = 6.7 Hz, 1H, *i*Pr-C*H*$_3$), 1.08 (d, ^{3}J = 6.7 Hz, 3H, *i*Pr-C*H*$_3$), 1.05–0.99 (m, 2H, Cy-C*H*$_2$), 0.76 (s, 3H, NC(C*H*$_3$)$_2$) ppm. $^{13}C\{^{1}H\}$ NMR (126 MHz, C_6D_6, 297 K): δ = 231.4 ($C_{carbene}$, identified by HMBC), 145.0 (*o*-Ar*C*), 132.9 (*i*-Ar*C*), 129.9 (*p*-Ar*C*), 125.0 (*m*-Ar*C*), 77.5 (N*C*(CH_3)$_2$), 58.2 (*C*(CH_3)$_2$), 45.5 (*C*H$_2$), 35.5 (Cy-*C*H$_2$), 30.4 (NC(*C*H$_3$)$_2$), 29.4 (*i*Pr-*C*H), 28.7 (NC(*C*H$_3$)$_2$), 25.9 (*i*Pr-*C*H$_3$), 25.0 (Cy-*C*H$_2$), 23.5 (*i*Pr-*C*H$_3$), 22.4 (Cy-*C*H$_2$) ppm. *Note: The C_{CN} resonance was not detected due to broadening caused by quadrupolar coupling with the $^{10/11}B$ nucleus.* ^{11}B NMR (160 MHz, C_6D_6, 297 K): δ = –32.3 (d, $^{1}J_{B–H}$ = 90.9 Hz) ppm. HRMS ASAP for $[C_{24}H_{37}BN_2]^+$ = $[M + H]^+$: calcd. 365.3123; found: 365.3113.

[(CAAC^{Cy})BH(CN)Li(TMEDA)], 2b-TMEDA

a) (CAAC^{Cy})BH_2(CN) (500 mg, 1.37 mmol) was dissolved in THF (20 mL) and cooled to –78 °C. Within 5 min *n*BuLi in hexane (2.50 M, 1.04 mL, 2.61 mmol, 1.90 equiv.) was added dropwise, whereupon the suspension turned yellow. The reaction mixture was warmed to room temperature and stirred for a further 2 h. All volatiles were removed *in vacuo* to give a sticky orange solid. Residual solvent was removed by dissolving the crude product in hexane (2 mL) following removal of all volatiles *in vacuo*. This procedure was repeated three times to yield **2b** as a fluffy orange solid (605 mg, 1.03 mmol, 75%). *Note: The purity of [(CAAC^{Cy})BH(CN)Li(THF)$_3$]* ***2b*** *is sufficient for reactivity studies. All attempts to crystallize the compound failed due to its high solubility in common organic solvents, even at –70 °C. In order to yield analytically pure material the TMEDA adduct was synthesized as follows:* TMEDA (23.8 mg, 222 µmol) was added to a solution of [(CAAC^{Cy})BH(CN)Li(THF)$_3$] (100 mg, 171 µmol) in benzene (3 mL). The solution was stirred for 30 min at room

temperature, whereupon a yellow precipitate formed. After removal of the solvent *in vacuo* the solid was washed with hexane (2 mL) and dried *in vacuo* to yield **2b-TMEDA** as a yellow solid (69.1 mg, 142 µmol, 83%).

b) ($CAAC^{Cy}$)BH_2(CN) (250 mg, 686 µmol) was suspended in hexane (15 mL) and TMEDA (200 µL, 1.33 mmol, 1.90 equiv.) was added. After cooling the reaction to –35 °C *n*BuLi in hexane (2.5 M, 520 µL, 1.30 mmol, 1.90 equiv.) was added dropwise, whereupon the suspension turned light yellow. After stirring for 15 min at –35 °C the orange solution was warmed to room temperature and stirred for a further 1.5 h. All volatiles were removed *in vacuo* and the crude product was recrystallized from hexane (2 mL) to yield **2b-TMEDA** (207 mg, 426 mmol, 62%). *Note: Evaporation of the solvent in vacuo resulted in some hydrolysis to compound **1b**.* $^{1}H\{^{11}B\}$ NMR (500 MHz, C_6D_6, 297 K): δ = 7.27–7.25 (m, 3H, Ar*H*), 3.56 (sept, ^{3}J = 6.8 Hz, 2H, *i*Pr-C*H*), 2.36–2.27 (m, 2H, Cy-CH_2), 2.09 (s, 2H, CH_2), 2.08–2.06 (m, 2H, Cy-CH_2), 2.04 (s, 12H, TMEDA-CH_3), 1.84 (s, 4H, TMEDA-CH_2), 1.82–1.59 (m, 6H, Cy-CH_2), 1.81 (s, 1H, B*H*), 1.54 (d, ^{2}J = 6.7 Hz, 6H, *i*Pr-CH_3), 1.43 (d, ^{3}J = 6.8 Hz, 6H, *i*Pr-CH_3), 1.30 (s, 6H, NC(CH_3)$_2$) ppm. $^{13}C\{^{1}H\}$ NMR (126 MHz, C_6D_6, 297 K): δ = 150.8 (*o*-Ar*C*), 139.5 (*i*-Ar*C*), 126.9 (*p*-Ar*C*), 124.2 (*m*-Ar*C*), 64.1 (N*C*(CH$_3$)$_2$), 56.6 (TMEDA-*C*H$_2$), 51.4 (*C*H$_2$), 46.0 (TMEDA-*C*H$_3$), 42.0 (Cy-*C*H$_2$), 30.1 (NC(*C*H$_3$)$_2$), 28.7 (*i*Pr-*C*H), 27.5 (*i*Pr-*C*H$_3$), 26.9 (Cy-*C*H$_2$), 24.8 (Cy-*C*H$_2$), 24.4 (*i*Pr-*C*H$_3$) ppm. *Note: The C_{CN} and $C_{carbene}$ resonances were not detected, even by HMBC, due to broadening caused by quadrupolar coupling with the $^{10/11}B$ nucleus.* ^{11}B NMR (160 MHz, C_6D_6, 297 K): δ = –12.9 (d, $^{1}J_{B–H}$ = 98.3 Hz) ppm. $^{7}Li\{^{1}H\}$ NMR (194.4 MHz, C_6D_6, 297 K): δ = –0.24 ppm. UV-vis (THF, 25 °C): λ_{max} = 331 nm.

($CAAC^{Me}$)BH(CN)(COMe), $4a^{COMe}$

Acetyl chloride (30.0 µL, 420 µmol, 1.99 equiv.) was quickly added to a stirred solution of **2a** (100 mg, 211 µmol) in benzene whereupon the orange color vanished and a colorless solid precipitated. After 5 min of stirring at room temperature the precipitate was filtered off and the filtrate dried *in vacuo*. The resulting solid was washed with hexane (4 mL) and diethyl ether (0.5 mL), dried *in vacuo* and extracted with dichloromethane. Removal of the solvent and drying *in vacuo* afforded **$4a^{COMe}$** as an off-white solid (64.3 mg, 175 µmol, 83%). Colorless single crystals were obtained by slow evaporation of a saturated hexane solution. $^{1}H\{^{11}B\}$ NMR (500 MHz, C_6D_6, 297 K): δ = 7.07 (t, ^{3}J = 7.8 Hz, 1H, *p*-Ar*H*), 6.97 (dd, ^{3}J = 7.8 Hz, ^{4}J = 1.5 Hz, 1H, *m*-Ar*H*), 6.92 (dd, ^{3}J = 7.7 Hz, ^{4}J = 1.5 Hz, 1H, *m*-Ar*H*), 3.04 (sept, ^{3}J = 6.5 Hz, 1H, *i*Pr-C*H*), 2.53 (sept, ^{3}J = 6.5 Hz, 1H, *i*Pr-C*H*), 2.39 (s, 3H, COCH_3), 2.21 (s,

1H, B*H*), 1.59 (s, 3H, C(C*H*$_3$)), 1.57 (d, ^2J = 12.8 Hz, 1H, C*H*$_2$), 1.52 (s, 3H, NC(C*H*$_3$)), 1.35 (d, ^2J = 12.8 Hz, 1H, C*H*$_2$), 1.33 (d, ^3J = 6.7 Hz, 3H, *i*Pr-C*H*$_3$), 1.11 (d, ^3J = 6.7 Hz, 3H, *i*Pr-C*H*$_3$), 1.04 (d, ^3J = 6.7 Hz, 3H, *i*Pr-C*H*$_3$), 0.94 (d, ^3J = 6.7 Hz, 3H, *i*Pr-C*H*$_3$), 0.85 (s, 3H, C(C*H*$_3$)$_2$), 0.70 (s, 3H, NC(C*H*$_3$)$_2$) ppm. ^{13}C{^{1}H} NMR (126 MHz, C_6D_6, 297 K): δ = 240.9 (*C*OCH$_3$, identified by HMBC), 227.6 ($C_{carbene}$, identified by HMBC), 146.5 (*o*-Ar*C*), 145.0 (*o*-Ar*C*), 132.6 (*i*-Ar*C*), 130.0 (*p*-Ar*C*), 125.4 (*m*-Ar*C*), 124.8 (*m*-Ar*C*), 78.8 (N*C*(CH$_3$)$_2$), 53.7 (*C*(CH$_3$)$_2$), 49.8 (*C*H$_2$), 35.6 (CO*C*H$_3$) 30.4 (NC(*C*H$_3$)$_2$), 30.4 (C(*C*H$_3$)$_2$), 29.0 (*i*Pr-*C*H), 28.8 (*i*Pr-*C*H), 28.0 (C(*C*H$_3$)$_2$), 27.7 (*i*Pr-*C*H$_3$), 26.3 (NC(*C*H$_3$)$_2$), 25.8 (*i*Pr-*C*H$_3$), 23.5 (*i*Pr-*C*H$_3$), 23.2 (*i*Pr-*C*H$_3$) ppm. *Note: The C_{CN} resonance was not detected due to broadening caused by quadrupolar coupling with the $^{10/11}$B nucleus.* ^{11}B NMR (160 MHz, C_6D_6, 297 K): δ = –23.8 (d, $^1J_{B–H}$ = 91.0 Hz) ppm. HRMS LIFDI for $[C_{23}H_{36}BN_2O]^+$ = $[M + H]^+$: calcd. 367.2915; found: 367.2914.

(CAAC^{Cy})BH(CN)(COMe), 4b^{COMe}

To a stirred solution of **2b** (100 mg, 171 µmol) acetyl chloride (25.0 µL, 350 µmol, 2.05 equiv.) was quickly added. The orange color vanished and a colorless solid precipitated. After 5 min of stirring at room temperature the precipitate was filtered off and the filtrate dried *in vacuo*. The resulting solid was washed with hexane (4 mL) and diethyl ether (0.5 mL), dried *in vacuo* and extracted with dichloromethane. Removal of the solvent and drying *in vacuo* afforded **4b^{COMe}** as an off-white solid (54.7 mg, 135 µmol, 79% yield). Colorless single crystals were obtained by slow evaporation of a saturated hexane solution. ^{1}H{^{11}B} NMR (500 MHz, C_6D_6, 297 K): δ = 7.08 (t, ^3J = 7.7 Hz, 1H, *p*-Ar*H*), 6.99 (dd, ^3J = 7.8 Hz, ^4J = 1.6 Hz, 1H, *m*-Ar*H*), 6.93 (dd, ^3J = 7.7 Hz, ^4J = 1.5 Hz, 1H, *m*-Ar*H*), 3.10 (sept, ^3J = 6.6 Hz, 1H, *i*Pr-C*H*), 2.97 (br dt, ^3J = 13.2 Hz, ^3J = 3.7 Hz, 1H, Cy-C*H*$_2$), 2.56 (sept, ^3J = 6.6 Hz, 1H, *i*Pr-C*H*), 2.42 (s, 3H, COC*H*$_3$), 2.25 (dt, ^3J = 13.5 Hz, ^3J = 3.7 Hz, 1H, Cy-C*H*$_2$), 2.24 (s, 1H, B*H*), 2.03–1.97 (m, 1H, Cy-C*H*$_2$), 1.75 (d, ^2J = 13.2 Hz, 1H, C*H*$_2$), 1.56–1.46 (m, 4H, Cy-C*H*$_2$), 1.44 (d, ^2J = 13.2 Hz, 1H, C*H*$_2$), 1.37–1.27 (m, 1H, Cy-C*H*$_2$),1.35 (d, ^3J = 6.7 Hz, 3H, *i*Pr-C*H*$_3$), 1.19–1.09 (m, 1H, Cy-C*H*$_2$), 1.15 (d, ^3J = 6.7 Hz, 3H, *i*Pr-C*H*$_3$), 1.03 (d, ^3J = 6.7 Hz, 3H, *i*Pr-C*H*$_3$), 1.00–0.92 (m, 1H, Cy-C*H*$_2$), 0.95 (d, ^3J = 6.4 Hz, 3H, *i*Pr-C*H*$_3$), 0.90 (s, 3H, NC(C*H*$_3$)), 0.70 (s, 3H, NC(C*H*$_3$)) ppm. ^{13}C{^{1}H} NMR (126 MHz, C_6D_6, 297 K): δ = 240.6 (*C*OCH$_3$, identified by HMBC), 227.1 ($C_{carbene}$, identified by HMBC), 146.6 (*o*-Ar*C*), 144.9 (*o*-Ar*C*), 132.7 (*i*-Ar*C*), 129.9 (*p*-Ar*C*), 125.4 (*m*-Ar*C*), 124.8 (*m*-Ar*C*), 78.9 (N*C*(CH$_3$)$_2$), 59.2 (*C*(C_5H_{10})), 44.1 (*C*H$_2$), 36.1 (Cy-*C*H$_2$), 35.6 (CO*C*H$_3$), 34.8 (Cy-*C*H$_2$), 30.8 (NC(*C*H$_3$)$_2$), 29.0 (*i*Pr-*C*H), 28.8 (*i*Pr-*C*H), 27.8 (*i*Pr-*C*H$_3$), 26.8 (NC(*C*H$_3$)$_2$), 25.7 (*i*Pr-*C*H$_3$), 25.1 (Cy-*C*H$_2$), 23.5 (*i*Pr-*C*H$_3$), 23.2 (*i*Pr-*C*H$_3$), 22.4 (Cy-

CH$_2$), 22.3 (Cy-CH$_2$) ppm. *Note: The C_{CN} resonance was not detected due to broadening caused by quadrupolar coupling with the $^{10/11}$B nucleus.* ^{11}B NMR (160 MHz, C_6D_6, 297 K): δ = –23.6 (d, $^1J_{B–H}$ = 90.0 Hz) ppm. HRMS LIFDI for [$C_{26}H_{39}BN_2O$] = [M]: calcd. 406.3150; found: 606.3146.

(CAAC^{Me})BH(CN)(COPh), 4a^{COPh}

Compound **2a** (50 mg, 105 µmol) was dissolved in diethyl ether (1.5 mL) and treated with benzoyl chloride (26.7 mg, 190 µmol, 1.80 equiv.) whereupon the orange color vanished and a colorless solid precipitated. After filtration, the filtrate was dried *in vacuo* and the resulting solid was washed with hexane (2 mL). Extraction with diethyl ether (1 mL) and slow evaporation of the concentrated solution at –30 °C yielded colorless single crystals of **4a^{COPh}** (35.7 mg, 83.3 µmol, 79%). ^{1}H{^{11}B} NMR (500 MHz, C_6D_6, 297 K): δ = 8.37–8.34 (m, 2H, *o*-Ph*H*), 7.15–7.11 (m, 2H, *m*-Ph*H*), 7.10–7.06 (m, 1H, *p*-Ph*H*), 7.02 (t, ^3J = 7.7 Hz, 1H, *p*-Ar*H*), 6.95 (dd, ^3J = 7.8 Hz, ^4J = 1.6 Hz, 1H, *m*-Ar*H*), 6.87 (dd, ^3J = 7.7 Hz, ^4J = 1.6 Hz, 1H, *m*-Ar*H*), 3.06 (sept, ^3J = 6.6 Hz, 1H, *i*Pr-C*H*), 2.87 (s, 1H, B*H*), 2.69 (sept, ^3J = 6.7 Hz, 1H, *i*Pr-C*H*), 1.71 (d, ^2J = 12.9 Hz, 1H, CH_2), 1.63 (d, ^3J = 6.9 Hz, 3H, *i*Pr-CH_3), 1.43 (d, ^3J = 6.7 Hz, 3H, *i*Pr-CH_3), 1.39 (d, ^2J = 12.9 Hz, 1H, CH_2), 1.07 (d, ^3J = 6.7 Hz, 3H, *i*Pr-CH_3), 1.07 (d, ^3J = 6.7 Hz, 3H, *i*Pr-CH_3), 0.90 (s, 3H, C(CH_3)$_2$), 0.80 (d, ^3J = 6.4 Hz, 3H, *i*Pr-CH_3), 0.76 (s, 3H, NC(CH_3)$_2$) ppm. ^{13}C{^{1}H} NMR (126 MHz, C_6D_6, 297 K): δ = 232.6 (*C*OPh, identified by HMBC), 229.1 ($C_{carbene}$, identified by HMBC), 146.7 (*o*-Ar*C*), 145.0 (*o*-Ar*C*), 142.7(*i*-Ph*H*), 131.7 (*p*-Ph*C*), 130.1 (*p*-Ar*C*), 128.4 (*m*-PhC), 128.3 (*o*-Ph*C*), 125.4 (*m*-Ar*C*), 124.8 (*m*-Ar*C*), 78.8 (N*C*(CH$_3$)$_2$), 54.2 (*C*(CH$_3$)$_2$), 50.0 (*C*H$_2$), 30.5 (NC(*C*H$_3$)$_2$), 30.4 (C(*C*H$_3$)$_2$), 29.0 (*i*Pr-*C*H), 28.9 (*i*Pr-*C*H), 28.0 (*i*Pr-*C*H$_3$), 26.3 (NC(*C*H$_3$)$_2$), 25.7 (*i*Pr-*C*H$_3$), 23.4 (*i*Pr-*C*H$_3$), 23.2 (*i*Pr-*C*H$_3$) ppm. ^{11}B NMR (160 MHz, C_6D_6, 297 K): δ = –24.5 (d, $^1J_{B–H}$ = 89.9 Hz) ppm. *Note: The C_{CN} resonance was not detected due to broadening caused by quadrupolar coupling with the $^{10/11}$B nucleus.* HRMS LIFDI for [$C_{28}H_{37}BN_2O$]$^+$ = [M + H]$^+$: calcd. 429.3072; found: 429.3073.

(CAAC^{Cy})BH(CN)(COPh), 4b^{COPh}

Compound **1b** (50 mg, 85.2 µmol) was dissolved in benzene and treated with benzoyl chloride (21.6 mg, 153 µmol, 1.80 equiv.), whereupon the orange color vanished and a colorless solid precipitated. After filtration, the filtrate was dried *in vacuo* and the resulting off-white solid washed with hexane (3 mL) and diethyl ether (1 mL). The residue was dried *in vacuo* to yield **4b^{COPh}** (29.2 mg, 62.2 µmol, 73%). Colorless single crystals were obtained by slow evaporation of a saturated hexane solution. ^{1}H{^{11}B} NMR (500 MHz, C_6D_6, 297 K): δ = 8.40–8.36 (m, 2H,

o-Ph*H*), 7.16–7.12 (m, 2H, *m*-Ph*H*), 7.11–7.07 (m, 1H, *p*-Ph*H*), 7.04 (t, ^3J = 7.7 Hz, 1H, *p*-Ar*H*), 6.96 (dd, ^3J = 7.7 Hz, ^4J = 1.2 Hz, 1H, *m*-Ar*H*), 6.91 (dd, ^3J = 7.7 Hz, ^4J = 1.2 Hz, 1H, *m*-Ar*H*), 3.13 (sept, ^3J = 6.6 Hz, 1H, *i*Pr-C*H*), 3.11–3.02 (m, 1H, Cy-CH_2), 2.91 (s, 1H, B*H*), 2.73 (sept, ^3J = 6.7 Hz, 1H, *i*Pr-C*H*), 2.36 (dt, ^3J = 13.6 Hz, ^3J = 3.4 Hz, 1H), 2.18–2.11 (m, 1H, Cy-CH_2), 1.84 (d, ^2J = 13.2 Hz, 1H, CH_2), 1.56–1.52 (m, 2H, Cy-CH_2), 1.51–1.42 (m, 2H, Cy-CH_2), 1.45 (d, ^3J = 6.6 Hz, 3H, *i*Pr-CH_3), 1.34–1.24 (m, 1H, Cy-CH_2), 1.22–1.13 (m, 1H, Cy-CH_2), 1.11 (d, ^3J = 6.6 Hz, 3H, *i*Pr-CH_3), 1.07 (d, ^3J = 6.6 Hz, 3H, *i*Pr-CH_3), 1.00–0.95 (m, 1H, Cy-CH_2), 0.97 (s, 3H, NC(CH_3)), 0.82 (d, ^3J = 6.4 Hz, 3H, *i*Pr-CH_3), 0.77 (s, 3H, NC(CH_3)) ppm. ^{13}C{^{1}H} NMR (126 MHz, C_6D_6, 297 K): δ = 231.8 (*C*OPh, identified by HMBC), 228.7 ($C_{carbene}$, identified by HMBC), 146.8 (*o*-Ar*C*), 145.0 (*o*-Ar*C*), 142.8 (*p*-Ph*C*), 132.8 (*i*-Ar*C*), 131.7 (*i*-Ph*C*), 130.0 (*p*-Ar*C*), 128.4 (*o*-Ph*C*), 128.3 (*m*-Ph*C*), 125.4 (*m*-Ar*C*), 124.8 (*m*-Ar*C*), 79.0 (N*C*(CH_3)$_2$), 59.2 (*C*(C_5H_{10})), 44.2 (*C*H_2), 35.9 (Cy-*C*H_2), 34.6 (Cy-*C*H_2), 30.9 (NC(*C*H_3)$_2$), 29.0 (*i*Pr-*C*H), 28.8 (*i*Pr-*C*H), 28.0 (*i*Pr-*C*H_3), 26.9 (NC(*C*H_3)$_2$), 25.8 (*i*Pr-*C*H_3), 25.1 (Cy-*C*H_2), 23.5 (*i*Pr-*C*H_3), 23.3 (*i*Pr-*C*H_3), 22.5 (Cy-*C*H_2), 22.5 (Cy-*C*H_2) ppm. *Note: The* C_{CN} *resonance was not detected due to broadening caused by quadrupolar coupling with the* $^{10/11}$*B nucleus.* ^{11}B NMR (160 MHz, C_6D_6, 297 K): δ = –24.3 (d, $^1J_{B–H}$ = 88.6 Hz) ppm. HRMS LIFDI for [$C_{31}H_{41}BN_2O$] = [M]: calcd. 468.3306; found: 468.3301.

(CAAC^{Me})BH(CN)(CH_2Cl), 4a^{CH2Cl}

To a solution of compound **2a** (100 mg, 211 µmol) in 3 mL of diethyl ether CH_2Cl_2 (32.2 mg, 379 µmol, 1.80 equiv.) was added. The orange solution turned dark and subsequently the color vanished, whereupon a colorless solid precipitated. After filtration and slow evaporation of the filtrate colorless single crystals of **4a^{CH2Cl}** were obtained (56.6 mg, 152 µmol, 72%). ^{1}H{^{11}B} NMR (500 MHz, C_6D_6, 297 K): δ = 7.09 (t, ^3J = 7.8 Hz, 1H, *p*-Ar*H*), 6.98 (dd, ^3J = 7.8 Hz, ^4J = 1.5 Hz, 1H, *m*-Ar*H*), 6.92 (dd, ^3J = 7.8 Hz, ^4J = 1.5 Hz, 1H, *m*-Ar*H*), 3.28 (dd, ^2J = 11.8 Hz, ^3J = 6.8 Hz, 1H, B(CH_2Cl)), 2.99 (dd, ^2J = 11.8 Hz, ^3J = 4.1 Hz, 1H, B(CH_2Cl)), 2.60 (sept, ^3J = 6.7 Hz, 1H, *i*Pr-C*H*), 2.30 (sept, ^3J = 6.7 Hz, 1H, *i*Pr-C*H*), 2.09 (dd, ^3J = 6.8 Hz, ^3J = 4.1 Hz, 1H, B*H*), 1.63 (s, 3H, (C(CH_3)$_2$), 1.50 (d, ^3J = 6.5 Hz, 3H, *i*Pr-CH_3), 1.37 (d, ^2J = 13.2 Hz, 1H, CH_2), 1.24 (d, ^2J = 13.2 Hz, 1H, CH_2), 1.16 (d, ^3J = 6.6 Hz, 3H, *i*Pr-CH_3), 1.14 (s, 3H, (C(CH_3)$_2$), 1.09 (d, ^3J = 6.6 Hz, 3H, *i*Pr-CH_3), 1.01 (d, ^3J = 6.6 Hz, 3H, *i*Pr-CH_3), 0.72 (s, 3H, (NC(CH_3)$_2$), 0.66 (s, 3H, (NC(CH_3)$_2$) ppm. ^{13}C{^{1}H} NMR (126 MHz, C_6D_6, 297 K): δ = 227.5 ($C_{carbene}$, identified by HMBC), 145.5 (*o*-Ar*C*), 144. 533 (*o*-Ar*C*), 132.4 (*i*-Ar*C*), 130.2 (*p*-Ar*C*), 125.4 (*m*-Ar*C*), 124.8 (*m*-Ar*C*), 78.7 (N*C*(CH_3)$_2$), 53.6 (*C*(CH_3)$_2$), 51.0 (*C*H_2), 37.5 (B(*C*H_2Cl), identified by HMBC), 30.1 (C(*C*H_3)$_2$), 29.5 (*i*Pr-*C*H), 29.3 (*i*Pr-*C*H),

28.5 (NC(CH_3)$_2$), 28.2 (C(CH_3)$_2$), 28.2 (NC(CH_3)$_2$), 27.3 (*i*Pr-CH_3), 26.3 (*i*Pr-CH_3), 23.7 (*i*Pr-CH_3), 23.5 (*i*Pr-CH_3) ppm. *Note: The C_{CN} resonance was not detected due to broadening caused by quadrupolar coupling with the $^{10/11}B$ nucleus.* ^{11}B NMR (160 MHz, C_6D_6, 297 K): δ = –23.9 (d, $^1J_{B–H}$ = 93.1 Hz) ppm. HRMS ASAP for $[C_{22}H_{35}BN_2Cl]^+$ = $[M + H]^+$: calcd. 373.2576; found: 373.2569.

(CAAC^{Cy})BH(CN)(CH_2Cl), 4b^{CH2Cl}

To a solution of compound **2b** (100.0 mg, 171 µmol) in benzene (0.6 mL) CH_2Cl_2 (26.1 mg, 307 µmol, 1.80 equiv.) was added, whereupon the orange color vanished and a colorless solid precipitated. After filtration, the filtrate was dried *in vacuo* and the resulting residue was washed with hexane (2 mL) and diethyl ether (0.5 mL). The colorless solid was dried *in vacuo* to yield **4b^{CH2Cl}** (50.1 mg, 121 µmol, 71%). Slow evaporation of a concentrated benzene solution gave colorless single crystals. ^{1}H{^{11}B} NMR (500 MHz, C_6D_6, 297 K): δ = 7.08 (t, ^3J = 7.8 Hz, 1H, *p*-Ar*H*), 6.98 (dd, ^3J = 7.8 Hz, ^4J = 1.5 Hz, 1H, *m*-Ar*H*), 6.92 (dd, ^3J = 7.8 Hz, ^4J = 1.5 Hz, 1H, *m*-Ar*H*), 3.35 (dd, ^2J = 11.8 Hz, ^3J = 7.05 Hz, 1H, B(CH_2Cl)), 3.02 (dd, ^2J = 11.8 Hz, ^3J = 3.9 Hz, 1H, B(CH_2Cl)), 2.80 (dt, ^2J = 13.4 Hz, ^3J = 3.8 Hz, 1H, Cy-CH_2), 2.62 (sept, ^3J = 6.6 Hz, 1H, *i*Pr-C*H*), 2.31 (sept, ^3J = 6.6 Hz, 1H, *i*Pr-C*H*), 2.14–2.05 (m, 1H, B*H*), 2.09 (dt, ^2J = 13.2 Hz, ^3J = 3.8 Hz, 1H, Cy-CH_2), 1.78–1.71 (m, 1H, Cy-CH_2), 1.55–1.48 (m, 1H, Cy-CH_2), 1.50 (d, ^3J = 6.6 Hz, 3H, *i*Pr-CH_3), 1.47–1.38 (m, 4H, Cy-CH_2, CH_2), 1.15 (d, ^3J = 6.6 Hz, 3H, *i*Pr-CH_3), 1.14–1.08 (m, 1H, Cy-CH_2), 1.10 (d, ^3J = 6.6 Hz, 3H, *i*Pr-CH_3), 1.02 (d, ^3J = 6.6 Hz, 3H, *i*Pr-CH_3), 1.01–0.87 (m, 3H, Cy-CH_2), 0.71 (s, 3H, NC(CH_3)), 0.66 (s, 3H, NC(CH_3)) ppm. ^{13}C{^{1}H} NMR (126 MHz, C_6D_6, 297 K): δ = 145.4 (*o*-Ar*C*), 144.1 (*o*-Ar*C*), 132.4 (*i*-Ar*C*), 130.0 (*p*-Ar*C*), 125.4 (*m*-Ar*C*), 124.7 (*m*-Ar*C*), 78.6 (N*C*(CH_3)$_2$), 59.4 (*C*(C_5H_{10})), 44.8 (*C*H_2), 38.1 (B(*C*H_2Cl), identified by HSQC), 36.6 (Cy-*C*H_2), 33.9 (Cy-*C*H_2), 29.5 (*i*Pr-*C*H), 29.4 (*i*Pr-*C*H), 29.0 (NC(*C*H_3)$_2$), 28.7 (NC(*C*H_3)$_2$), 27.4 (*i*Pr-*C*H_3), 26.3 (*i*Pr-*C*H_3), 24.9 (Cy-*C*H_2), 23.7 (*i*Pr-*C*H_3), 23.4 (*i*Pr-*C*H_3), 22.2 (Cy-*C*H_2) ppm. *Note: The C_{CN} and $C_{carbene}$ resonances were not detected, even by HMBC, due to broadening caused by quadrupolar coupling with the $^{10/11}B$ nucleus.* ^{11}B NMR (160 MHz, C_6D_6, 297 K): δ = –23.8 (d, $^1J_{B–H}$ = 94.9 Hz) ppm. HRMS ASAP for $[C_{25}H_{39}BN_2Cl]^+$ = $[M + H]^+$: calcd. 413.2889; found: 413.2884.

(CAAC^{Me})BH(CN)(*n*Bu), 4a^{nBu}

Compound **2a** (100 mg, 211 µmol) was dissolved in benzene (0.6 mL) and 1-bromobutane (40.0 µL, 440 µmol 1.99 equiv.) was added. The orange color vanished and a colorless solid precipitated. After filtration, the filtrate was dried *in vacuo* and the resulting residue was washed with a mixture of hexane (2 mL) and diethyl ether (0.5 mL). The colorless solid was dried *in vacuo* and extracted with dichloromethane. Slow evaporation of the concentrated solution yielded colorless single crystals of **4a^{nBu}** (48.9 mg, 129 µmol, 61%). ^{1}H{^{11}B} NMR (500 MHz, CD_2Cl_2, 297 K): δ = 7.43 (t, ^{3}J = 7.8 Hz, 1H, *p*-Ar*H*), 7.30 (dd, ^{3}J = 7.8 Hz, ^{4}J = 1.5 Hz, 1H, *m*-Ar*H*), 7.27 (dd, ^{3}J = 7.8 Hz, ^{4}J = 1.5 Hz, 1H, *m*-Ar*H*), 2.64 (sept, ^{3}J = 6.6 Hz, 1H, *i*Pr-C*H*), 2.57 (sept, ^{3}J = 6.6 Hz, 1H, *i*Pr-C*H*), 2.10 (d, ^{2}J = 13.2 Hz, 1H, CH_2), 2.05 (d, ^{2}J = 13.2 Hz, 1H, CH_2), 1.76 (s, 3H, C(CH_3)$_2$), 1.56 (s, 3H, C(CH_3)$_2$), 1.43–1.35 (m, 2H, B(CH_2)$_3$CH$_3$), 1.32 (d, ^{3}J = 6.6 Hz, 3H, *i*Pr-CH_3), 1.32 (s, 3H, (NC(CH_3)$_2$), 1.31 (d, ^{3}J = 6.6 Hz, 3H, *i*Pr-CH_3), 1.27 (s, 3H, (NC(CH_3)$_2$), 1.26 (d, ^{3}J = 6.6 Hz, 3H, *i*Pr-CH_3), 1.25–1.19 (m, 2H, B(CH_2)$_3$CH$_3$), 1.17–1.13 (m, 1H, B*H*), 1.08 (d, ^{3}J = 6.6 Hz, 3H, *i*Pr-CH_3), 1.00–0.92 (m, 1H, B(CH_2)$_3$CH$_3$) 0.82 (t, ^{3}J = 7.3 Hz, 3H, B(CH$_2$)$_3$CH_3), 0.68–0.59 (m, 1H, B(CH_2)$_3$CH$_3$), 0.32–0.24 (m, 1H, B(CH_2)$_3$CH$_3$) ppm. ^{13}C{^{1}H} NMR (126 MHz, CD_2Cl_2, 297 K): δ = 230.7 ($C_{carbene}$, identified by HMBC), 145.4 (*o*-Ar*C*), 144.8 (*o*-Ar*C*), 133.4 (*i*-Ar*C*), 129.9 (*p*-Ar*C*), 125.2 (*m*-Ar*C*), 124.9 (*m*-Ar*C*), 78.8 (N*C*(CH$_3$)$_2$), 54.2 (*C*(CH$_3$)$_2$), 51.7 (*C*H$_2$), 33.8 (B(*C*H$_2$)$_3$CH$_3$), 30.0 (C(*C*H$_3$)), 29.5 (*i*Pr-*C*H), 29.5 (*i*Pr-*C*H), 29.2 (C(*C*H$_3$)), 29.1 (NC(*C*H$_3$)$_2$), 29.1 (NC(*C*H$_3$)$_2$), 26.7 (B(*C*H$_2$)$_3$CH$_3$), 26.7 (*i*Pr-*C*H$_3$), 26.1 (*i*Pr-*C*H$_3$), 23.6 (*i*Pr-*C*H$_3$), 23.5 (*i*Pr-*C*H$_3$), 20.2 (B(*C*H$_2$)$_3$CH$_3$), 14.3 (B(CH$_2$)$_3$*C*H$_3$) ppm. *Note: The C_{CN} resonance was not detected due to broadening caused by quadrupolar coupling with the $^{10/11}$B nucleus.* ^{11}B NMR (160 MHz, CD_2Cl_2, 297 K): δ = –24.6 (d, $^{1}J_{B–H}$ = 91.9 Hz) ppm. HRMS ASAP for [$C_{25}H_{42}BN_2$] = [M + H]$^{+}$: calcd. 381.3436; found 381.3435.

(CAAC^{Cy})BH(CN)(*n*Bu), 4b^{nBu}

Compound **2b** (50 mg, 85.2 µmol) was dissolved in toluene (0.6 mL) and 1-bromobutane (20.0 µL, 185 µmol, 2.18 equiv.) was added. The orange color vanished within 20 min. After removal of all volatiles *in vacuo*, the residue was extracted with pentane (0.5 mL). Filtration and drying the filtrate *in vacuo* afforded **4b^{nBu}**. *Note: Due to its high solubility in common organic solvents all attempts to further purify or crystallize **4b^{nBu}** failed.* ^{1}H{^{11}B} NMR (400 MHz, CD_2Cl_2, 297 K): δ = 7.42 (t, ^{3}J = 7.8 Hz, 1H, *p*-Ar*H*), 7.29 (dd, ^{3}J = 7.8 Hz, ^{4}J = 1.5 Hz, 1H, *m*-Ar*H*), 7.26 (dd, ^{3}J = 7.8 Hz, ^{4}J = 1.5 Hz, 1H, *m*-Ar*H*), 2.65 (sept, ^{3}J = 6.6 Hz, 1H, *i*Pr-C*H*), 2.68–2.59 (m, 1H, Cy-CH_2), 2.56 (sept, ^{3}J = 6.6 Hz, 1H, *i*Pr-C*H*),

2.24 (d, ^2J = 13.3 Hz, 1H, CH_2), 2.13 (d, ^2J = 13.3 Hz, 1H, CH_2), 1.89–1.74 (m, 4H, Cy-CH_2), 1.61–1.55 (m, 1H, Cy-CH_2), 1.48–1.40 (m, 4H, Cy-CH_2, B(CH_2)$_3$CH$_3$), 1.32–1.24 (m, 15H, *i*Pr-CH_3, NC(CH_3)$_2$), 1.23–1.17 (m, 3H, Cy-CH_2, B(CH_2)$_3$CH$_3$), 1.69–1.15 (m, 1H, B*H*), 1.06 (d, ^3J = 6.6 Hz, 3H, *i*Pr-CH_3), 0.99–0.92 (m, 1H, B(CH_2)$_3$CH$_3$) 0.82 (t, ^3J = 7.2 Hz, 3H, B(CH$_2$)$_3$CH_3), 0.73–0.68 (m, 1H, B(CH_2)$_3$CH$_3$), 0.35–0.26 (m, 1H, B(CH_2)$_3$CH$_3$) ppm. ^{13}C{^{1}H} NMR (100 MHz, CD$_2$Cl$_2$, 297 K): δ = 145.4 (*o*-Ar*C*), 144.7 (*o*-Ar*C*), 132.9 (*i*-Ar*C*, identified by HMBC), 129.8 (*p*-Ar*C*), 125.2 (*m*-Ar*C*), 124.8 (*m*-Ar*C*), 78.9 (N*C*(CH$_3$)$_2$), 59.9 (*C*(C$_5$H$_{10}$)), 45.3 (*C*H$_2$), 36.7 (Cy-*C*H$_2$), 34.9 (Cy-*C*H$_2$), 33.9 (B(*C*H$_2$)$_3$CH$_3$), 29.6 (*i*Pr-*C*H), 29.5 (*i*Pr-*C*H), 29.5 (NC(*C*H$_3$)$_2$), 29.4 (NC(*C*H$_3$)$_2$), 26.8 (*i*Pr-*C*H$_3$), 26.7 (B(*C*H$_2$)$_3$CH$_3$), 26.1 (*i*Pr-*C*H$_3$), 25.5 (Cy-*C*H$_2$), 23.6 (*i*Pr-*C*H$_3$), 23.5 (*i*Pr-*C*H$_3$), 22.6 (Cy-*C*H$_2$), 22.5 (Cy-*C*H$_2$), 14.4 (B(CH$_2$)$_3$*C*H$_3$) ppm. *Note: The C_{CN} and $C_{carbene}$ resonances were not detected, even by HMBC, due to broadening caused by quadrupolar coupling with the $^{10/11}$B nucleus.* ^{11}B NMR (128 MHz, CD$_2$Cl$_2$, 297 K): δ = –24.4 (d, $^1J_{B–H}$ = 89.7 Hz) ppm. HRMS ASAP for [C$_{28}$H$_{46}$BN$_2$] = [M + H]$^+$: calcd. 421.3749; found 421.3745.

(CAAC^{Me})BH(CN)(C$_3$H$_5$), 4a^{C3H5}

To a solution of compound **2a** (100 mg, 211 µmol) in benzene (0.6 mL) allyl bromide (38.0 mg, 314 µmol, 1.5 equiv.) was added, whereupon the orange color vanished. After removal of all volatiles *in vacuo* the residue was washed with hexane (2 mL) and diethyl ether (1 mL). The off-white solid was dried *in vacuo* and extracted with toluene. After filtration, the filtrate was dried *in vacuo* to afford **4a^{C3H5}** (56.8 mg, 156 µmol, 74%). Slow evaporation of the washing solution yielded colorless single crystals of **4a^{C3H5}**. ^{1}H{^{11}B} NMR (500 MHz, C$_6$D$_6$, 297 K): δ = 7.07 (t, ^3J = 7.8 Hz, 1H, *p*-Ar*H*), 6.97 (dd, ^3J = 7.8 Hz, ^4J = 1.4 Hz, 1H, *m*-Ar*H*), 6.91 (dd, ^3J = 7.8 Hz, ^4J = 1.4 Hz, 1H, *m*-Ar*H*), 6.39–6.29 (m, 1H, B(CH$_2$C*H*CH$_2$)), 5.29–5.22 (m, 1H, B(CH$_2$CHCH_2)), 5.06–5.02 (m, 1H, B(CH$_2$CHCH_2)), 2.64 (sept, ^3J = 6.7 Hz, 1H, *i*Pr-C*H*), 2.35 (sept, ^3J = 6.7 Hz, 1H, *i*Pr-C*H*), 1.97–1.89 (m, 1H, B(CH_2CHCH$_2$)), 1.71 (s, 3H, (C(CH_3)$_2$), 1.48 (d, ^3J = 6.5 Hz, 3H, *i*Pr-CH_3), 1.46–1.41 (m, 2H, B(CH_2CHCH$_2$), CH_2), 1.25 (d, ^2J = 13.2 Hz, 1H, CH_2), 1.20 (s, 3H, (C(CH_3)$_2$), 1.13–1.09 (m, 6H, *i*Pr-CH_3), 1.03 (d, ^3J = 6.6 Hz, 3H, *i*Pr-CH_3), 0.72 (s, 3H, (NC(CH_3)$_2$), 0.69 (s, 3H, (NC(CH_3)$_2$) ppm. *Note: The BH resonance was not detected due to overlap with other resonances.* ^{13}C{^{1}H} NMR (126 MHz, CDCl$_3$, 297 K): δ = 228.3 ($C_{carbene}$, identified by HMBC), 144.2 (*o*-Ar*C*), 144.1 (*o*-Ar*C*), 132.3 (*i*-Ar*C*), 142.7 (B(CH$_2$*C*HCH$_2$)), 129.8 (*p*-Ar*C*), 125.1 (*m*-Ar*C*), 124.6 (*m*-Ar*C*), 110.7 (B(CH$_2$CH*C*H$_2$)), 78.7 (N*C*(CH$_3$)$_2$), 54.0 (*C*(CH$_3$)$_2$), 51.6 (*C*H$_2$), 30.0 (C(*C*H$_3$)), 29.4 (*i*Pr-*C*H), 29.1 (NC(*C*H$_3$)$_2$), 28.7 (NC(*C*H$_3$)$_2$), 26.9 (*i*Pr-*C*H$_3$), 26.2 (*i*Pr-*C*H$_3$), 25.7

(B(CH_2CHCH$_2$)), 23.6 (*i*Pr-CH_3), 23.5 (*i*Pr-CH_3) ppm. *Note: The C_{CN} resonance was not detected due to broadening caused by quadrupolar coupling with the $^{10/11}$B nucleus.* ^{11}B NMR (160 MHz, C_6D_6, 297 K): δ = –24.7 (d, $^1J_{B–H}$ = 91.1 Hz) ppm. HRMS ASAP for [$C_{24}H_{38}BN_2$] = [M + H]$^+$: calcd. 365.3123; found 365.3120.

(CAAC^{Cy})BH(CN)(C_3H_5), 4b^{C3H5}

To a solution of **2b** (50 mg, 85.2 µmol) in benzene (0.6 mL) allyl bromide (15.5 mg, 128 µmol, 1.5 eqiv.) was added, whereupon the orange color vanished. All volatiles were removed *in vacuo* and the residual off-white solid was washed with hexane (2 mL) and diethyl ether (0.5 mL). Extraction with benzene (0.6 mL) and drying the filtrate *in vacuo* yielded **4b^{C3H5}** (30.0 mg, 74.2 µmol, 87%). Slow evaporation of the washing solution yielded colorless crystals of **4b^{C3H5}**. ^{1}H{^{11}B} NMR (500 MHz, C_6D_6, 297 K): δ = 7.09 (t, ^3J = 7.6 Hz, 1H, *p*-Ar*H*), 7.06–7.02 (m, 1H, *m*-Ar*H*), 6.92 (dd, ^3J = 7.5 Hz, ^4J = 1.6 Hz, 1H, *m*-Ar*H*), 6.38–6.26 (m, 1H, B(CH$_2$C*H*CH$_2$)), 5.29–5.22 (m, 1H, B(CH$_2$CHC*H*$_2$)), 5.04 (dd, ^3J = 10.0 Hz, ^4J = 2.5 Hz, 1H, B(CH$_2$CHC*H*$_2$)), 2.85–2.74 (m, 2H, Cy-C*H*$_2$), 2.39 (sept, ^3J = 6.6 Hz, 1H, *i*Pr-C*H*), 2.86–2.77 (dt, ^3J = 13.1 Hz, ^3J = 3.7 Hz, 2H, Cy-C*H*$_2$), 2.11–1.99 (m, 2H, Cy-C*H*$_2$), 1.81–1.73 (m, 1H, *i*Pr-C*H*), 1.66–1.58 (m, 2H), 1.55 (d, ^3J = 6.6 Hz, 3H, *i*Pr-C*H*$_3$), 1.52–1.44 (m, 3H), 1.34–1.39 (m, 1H, Cy-C*H*$_2$), 1.27–1.16 (m, 5H, Cy-C*H*$_2$, *i*Pr-C*H*$_3$), 1.12 (d, ^3J = 6.6 Hz, 3H, *i*Pr-C*H*$_3$), 1.10–1.06 (m, 1H, Cy-C*H*$_2$), 1.04 (d, ^3J = 6.6 Hz, 3H, *i*Pr-C*H*$_3$), 0.84 (s, 3H, (NC(C*H*$_3$)$_2$), 0.69 (s, 3H, (NC(C*H*$_3$)$_2$) ppm. *Note: The B*H *resonance was not detected due to overlap with other resonances.* ^{13}C{^{1}H} NMR (126 MHz, C_6D_6, 297 K): δ = 145.6 (*o*-Ar*C*), 144.1 (*o*-Ar*C*), 143.1 (B(CH$_2$*C*HCH$_2$)), 132.8 (*i*-Ar*C*), 129.8 (*p*-Ar*C*), 125.3 (*m*-Ar*C*), 124.4 (*m*-Ar*C*), 111.2 (B(CH$_2$CH*C*H$_2$)), 78.1 (N*C*(CH$_3$)$_2$), 54.0 (*C*(C$_5$H$_{10}$)), 44.7 (*C*H$_2$), 36.7 (Cy-*C*H$_2$), 34.3 (Cy-*C*H$_2$), 29.5 (*i*Pr-*C*H), 29.1 (NC(*C*H$_3$)$_2$), 29.1 (*i*Pr-*C*H), 28.5 (NC(*C*H$_3$)$_2$), 27.3 (*i*Pr-*C*H$_3$), 26.2 (*i*Pr-*C*H$_3$), 25.7 (B(*C*H$_2$CHCH$_2$)), 23.6 (*i*Pr-*C*H$_3$), 23.2 (*i*Pr-*C*H$_3$), 22.3 (Cy-*C*H$_2$), 22.3 (Cy-*C*H$_2$) ppm. *Note: The C_{CN} and $C_{carbene}$ resonances were not detected, even by HMBC, due to broadening caused by quadrupolar coupling with the $^{10/11}$B nucleus.* ^{11}B NMR (160 MHz, C_6D_6, 297 K): δ = –24.7 (d, $^1J_{B–H}$ = 87.6 Hz) ppm. HRMS LIFDI for [$C_{27}H_{42}BN_2$] = [M + H]$^+$: calcd. 405.3436; found 405.3434.

(CAAC^{Me})BH(CN)Br, 4a^{Br}

To a solution of compound **2a** (50.0 mg, 105 µmol) in benzene (0.6 mL) 1,2-dibromopropane (25.3 µL, 242 µmol, 2.30 equiv.) was added, whereupon an evolution of gas was observed and the orange color vanished. After filtration, the filtrate was dried *in vacuo*. The resulting residue

was washed with hexane (2 mL) and a mixture of dichloromethane (0.2 mL) and diethyl ether (1 mL). Extraction with tetrahydrofuran (0.5 mL) and slow evaporation of the concentrated solution yielded colorless single crystals of **4a$^{\mathbf{Br}}$** (39.0 mg, 96.7 µmol, 92%). ^{1}H{^{11}B} NMR (500 MHz, $CDCl_3$, 297 K): δ = 7.44 (t, ^{3}J = 7.8 Hz, 1H, *p*-Ar*H*), 7.31–7.26 (m, 2H, *m*-Ar*H*), 2.63 (sept, ^{3}J = 6.6 Hz, 1H, *i*Pr-C*H*), 2.56 (sept, ^{3}J = 6.6 Hz, 1H, *i*Pr-C*H*), 2.25 (br s, 1H, B*H*), 2.18 (d, ^{2}J = 13.2 Hz, 3H, CH_2), 2.15 (d, ^{2}J = 13.2 Hz, 1H, CH_2), 1.90 (s, 3H, (C(CH_3)$_2$), 1.87 (s, 3H, (C(CH_3)$_2$), 1.41 (s, 3H, (NC(CH_3)$_2$), 1.33 (s, 3H, (NC(CH_3)$_2$), 1.32 (d, ^{3}J = 6.7 Hz, 6H, *i*Pr-CH_3), 1.25 (d, ^{3}J = 6.7 Hz, 3H, *i*Pr-CH_3), 1.24 (d, ^{3}J = 6.7 Hz, 3H, *i*Pr-CH_3) ppm. ^{13}C{^{1}H} NMR (126 MHz, $CDCl_3$, 297 K): δ = 218.3 (C_{carbene}, identified by HMBC), 145.1 (*o*-Ar*C*), 144.8 (*o*-Ar*C*), 131.8 (*i*-Ar*C*), 130.4 (*p*-Ar*C*), 125.5 (*m*-Ar*C*), 125.1 (*m*-Ar*C*), 79.5 (N*C*(CH_3)$_2$), 54.3 (*C*(CH_3)$_2$), 51.4 (*C*H_2), 31.9 (C(*C*H_3)), 30.1 (C(*C*H_3)), 29.3 (*i*Pr-*C*H), 29.3 (*i*Pr-*C*H), 29.2 (NC(*C*H_3)$_2$), 28.3 (NC(*C*H_3)$_2$), 27.8 (*i*Pr-*C*H_3), 26.3 (*i*Pr-*C*H_3), 23.8 (*i*Pr-*C*H_3), 23.7 (*i*Pr-*C*H_3) ppm. *Note: The C_{CN} resonance was not detected due to broadening caused by quadrupolar coupling with the $^{10/11}$B nucleus.* ^{11}B NMR (160 MHz, $CDCl_3$, 297 K): δ = –25.1 (d, $^{1}J_{B–H}$ = 100.4 Hz) ppm. HRMS ASAP for [$C_{21}H_{33}BN_2Br$] = [M + H]$^+$: calcd. 403.1915; found 403.1911.

(CAAC$^{\mathbf{Cy}}$)BH(CN)Br, 4b$^{\mathbf{Br}}$

To a solution of compound **2b** (50.0 mg, 85.2 µmol) in benzene (0.6 mL) 1,2-dibromopropane (20.0 µL, 171 µmol, 2.30 equiv.) was added, whereupon an evolution of gas was observed and the orange color vanished. After filtration, the filtrate was dried *in vacuo*. The resulting residue was washed with hexane (2 mL) and a mixture of dichloromethane (0.2 mL) and diethyl ether (1 mL). Extraction with tetrahydrofuran (0.5 mL) and slow evaporation of the concentrated solution yielded colorless single crystals of **4b$^{\mathbf{Br}}$** (32.5 mg, 73.3 µmol, 86%). ^{1}H{^{11}B} NMR (500 MHz, $CDCl_3$, 297 K): δ = 7.43 (t, ^{3}J = 7.8 Hz, 1H, *p*-Ar*H*), 7.30–7.25 (m, 2H, *m*-Ar*H*), 2.86–2.77 (m, 2H, Cy-CH_2), 2.65 (sept, ^{3}J = 6.5 Hz, 1H, *i*Pr-C*H*), 2.56 (sept, ^{3}J = 6.5 Hz, 1H, *i*Pr-C*H*), 2.33 (d, ^{2}J = 13.3 Hz, 1H, CH_2), 2.27 (br s, 1H, B*H*), 2.19 (d, ^{2}J = 13.3 Hz, 1H, CH_2), 1.95–1.73 (m, 5H, Cy-CH_2), 1.52–1.43 (m, 2H, Cy-CH_2), 1.41 (s, 3H, CH_3), 1.38–1.33 (m, 5H, Cy-CH_2), 1.32–1.29 (m, 9H, CH_3, *i*Pr-CH_3, *i*Pr-CH_3), 1.24 (d, ^{3}J = 6.6 Hz, 3H, *i*Pr-CH_3), 1.24 (d, ^{3}J = 6.6 Hz, 3H, *i*Pr-CH_3) ppm. ^{13}C{^{1}H} NMR (126 MHz, $CDCl_3$, 297 K): δ = 217.1 (C_{carbene}, identified by HMBC), 145.2 (*o*-Ar*C*), 144.8 (*o*-Ar*C*), 131.9 (*i*-Ar*C*), 130.3 (*p*-Ar*C*), 125.5 (*m*-Ar*C*), 125.1 (*m*-Ar*C*), 79.5 (N*C*(CH_3)), 59.8 (*C*(C_5H_{10})), 45.0 (*C*H_2), 38.2 (Cy-*C*H_2), 35.6 (Cy-*C*H_2), 30.0 (NC(*C*H_3)), 29.3 (*i*Pr-*C*H), 29.1 (*i*Pr-*C*H), 28.8 (NC(*C*H_3)), 28.0 (*i*Pr-*C*H_3), 26.2 (*i*Pr-*C*H_3), 24.8 (Cy-*C*H_2), 23.8 (*i*Pr-*C*H_3), 23.7 (*i*Pr-*C*H_3), 22.7 (Cy-*C*H_2), 22.4 (Cy-

CH_2) ppm. *Note: The C_{CN} resonance was not detected due to broadening caused by quadrupolar coupling with the $^{10/11}B$ nucleus.* ^{11}B NMR (160 MHz, $CDCl_3$, 297 K): δ = –24.9 (d, $^1J_{B–H}$ = 99.4 Hz) ppm. HRMS ASAP for [$C_{24}H_{37}BN_2Br$] = [M + H]$^+$: calcd. 443.2228; found 443.2225.

(CAAC^{Me})BH(CN–BMes$_2$), 5a^{Mes}

Compound **2a** (100 mg, 211 µmol) and Mes_2BF (35.5 mg, 211 µmol) were dissolved in 3 mL of hexane, whereupon the reaction mixture turned dark red. The precipitate was filtered off and slow evaporation of the filtrate yielded red single crystals, which were washed with one small portion of hexane (0.3 mL) and dried *in vacuo* to yield **5a^{Mes}** (107 mg, 187 µmol, 89%). ^{1}H{^{11}B} NMR (500 MHz, C_6D_6, 297 K): δ = 7.20 (t, ^3J = 7.7 Hz, 1H, *p*-Ar*H*), 7.09 (d, ^3J = 7.7 Hz, 2H, *m*-Ar*H*), 6.92 (br s, 4H, Mes*H*), 2.93 (sept, ^3J = 6.7 Hz, 1H, *i*Pr-C*H*), 2.77 (br s, B*H*), 2.44 (s, 12H, *o*-MesCH_3), 2.17 (br s, 6H, *p*-MesCH_3), 1.58 (s, 2H, CH_2), 1.48 (s, 6H, CH_3), 1.39 (d, ^3J = 6.8 Hz, 6H, *i*Pr-CH_3), 1.20 (d, ^3J = 6.8 Hz, 6H, *i*Pr-CH_3), 0.95 (s, 6H, NC(CH_3)$_2$) ppm. ^{13}C{^{1}H} NMR (126 MHz, C_6D_6, 297 K): δ = 209.9 ($C_{carbene}$, identified by HMBC), 147.5 (*o*-Ar*C*), 142.7 (Mes*C*), 140.9 (Mes*C*), 138.2 (*o*-Mes*C*), 134.9 (*i*-Ar*C*), 129.0 (*p*-Ar*C*), 128.8 (*m*-Mes*C*), 124.9 (*m*-Ar*C*), 70.7 (*C*(CH_3)), 53.5 (*C*H_2), 48.2 (N*C*(CH_3)), 32.8 (C(*C*H_3)), 29.2 (*i*Pr-*C*H), 28.9 (NC(*C*H_3)), 26.7 (*i*Pr-*C*H_3), 23.6 (*i*Pr-*C*H_3), 23.1 (*o*-Mes*C*H_3), 21.4 (*p*-Mes*C*H_3) ppm. *Note: The C_{CN} resonance was not detected due to broadening caused by quadrupolar coupling with the $^{10/11}B$ nucleus.* ^{11}B NMR (160 MHz, C_6D_6, 297 K): δ = 36.2 (br, *B*Mes$_2$), –9.4 (d, $^1J_{B–H}$ = 109.1 Hz, *B*H) ppm. HRMS LIFDI for [$C_{39}H_{54}B_2N_2$] = [M]: calcd. 572.4468; found 572.4473. UV-vis (hexane, 25 °C): λ^1_{max} = 516 nm, λ^2_{max} = 381 nm.

(CAAC^{Cy})BH(CN–BMes$_2$), 5b^{Mes}

Compound **2b** (100 mg, 171 µmol) and Mes_2BF (45.7 mg, 171 µmol) were dissolved in 3 mL of hexane whereby the reaction mixture turned dark red. The precipitate was filtered off and slow evaporation of the solvent gave red single crystals which were washed with one small portion of hexane (0.3 mL) and dried *in vacuo* to yield **5b^{Mes}** (77.3 mg, 126 µmol, 74%). ^{1}H{^{11}B} NMR (500 MHz, C_6D_6, 297 K): δ = 7.20 (t, ^3J = 7.7 Hz, 1H, *p*-Ar*H*), 7.09 (d, ^3J = 7.7 Hz, 2H, *m*-Ar*H*), 6.79 (br s, 4H, Mes*H*), 2.95 (sept, ^3J = 6.7 Hz, 1H, *i*Pr-C*H*), 2.79 (br s, B*H*), 2.44 (s, 12H, *o*-MesCH_3), 2.27–2.13 (br m, 8H, *p*-MesCH_3, Cy-CH_2), 1.74–1.67 (m, 2H, Cy-CH_2), 1.74 (s, 2H, CH_2), 1.52–1.45 (m, 2H, Cy-CH_2), 1.40 (d, ^3J = 6.7 Hz, 6H, *i*Pr-CH_3), 1.22 (d, ^3J = 6.7 Hz, 6H, *i*Pr-CH_3), 1.19–1.12 (m, 2H, Cy-CH_2), 0.98 (s, 6H, NC(CH_3)$_2$), 0.74–0.61 (m, 2H, Cy-CH_2) ppm. ^{13}C{^{1}H} NMR (126 MHz, C_6D_6, 297 K): δ = 147.5 (*o*-Ar*C*),

143.1 (Mes*C*), 140.8 (Mes*C*), 137.6 (Mes*C*), 135.0 (*i*-Ar*C*), 129.0 (*p*-Ar*C*), 128.6 (*m*-Mes*C*), 124.9 (*m*-Ar*C*), 71.0 (N*C*(CH_3)), 53.6 (*C*(C_5H_{10})), 47.8 (*C*H_2), 40.1 (Cy-*C*H_2), 29.4 (*i*Pr-*C*H), 29.3 (NC(*C*H_3)), 26.7 (*i*Pr-*C*H_3), 24.8 (Cy-CH_2), 23.7 (*i*Pr-*C*H_3), 23.6 (Cy-CH_2), 23.1 (*o*-Mes*C*H_3), 21.3 (*p*-Mes*C*H_3) ppm. *Note: The C_{CN} and $C_{carbene}$ resonances were not detected, even by HMBC, due to broadening caused by quadrupolar coupling with the $^{10/11}B$ nucleus.* ^{11}B NMR (160 MHz, C_6D_6, 297 K): δ = 35.6 (br, *B*Mes_2), –9.1 (d, $^1J_{B–H}$ = 106.1 Hz, *B*H) ppm. HRMS LIFDI for [$C_{42}H_{58}B_2N_2$] = [M]: calcd. 612.4777; found 612.4781. UV-vis (hexane, 25 °C): λ^1_{max} = 520 nm, λ^2_{max} = 380 nm.

(CAAC^{Me})BH(CN–B(N*i*Pr$_2$)$_2$), 5a^{NiPr2}

Compound **2a** (100 mg, 211 µmol) and (*i*$Pr_2N)_2$BCl (93.9 mg, 379 µmol, 1.80 equiv.) were dissolved in benzene (1 mL) and heated at 60 °C overnight. The reaction mixture turned bright orange and a colorless solid precipitated. All volatiles were removed *in vacuo* and the residue was extracted with hexane. Slow evaporation of the saturated hexane solution yielded orange single crystals, which were washed with one portion of hexane (0.3 mL) and dried *in vacuo* to yield **5a^{NiPr2}** (85.8 mg, 160 µmol, 76%). ^{1}H{^{11}B} NMR (500 MHz, C_6D_6, 297 K): δ = 7.23–7.16 (m, 3H, *p*-Ar*H*, *m*-Ar*H*), 3.39 (sept, ^3J = 6.8 Hz, 4H, N(*i*Pr-C*H*)), 3.29 (sept, ^3J = 6.8 Hz, 2H, *i*Pr-C*H*), 2.35 (br s, 1H, B*H*), 1.90 (s, 2H, C*H*$_2$), 1.78 (s, 6H, C*H*$_3$), 1.54 (d, ^3J = 6.8 Hz, 6H, *i*Pr-C*H*$_3$), 1.32 (d, ^3J = 6.8 Hz, 6H, *i*Pr-C*H*$_3$), 1.17 (s, 6H, NC(C*H*$_3$)), 1.13 (d, ^3J = 6.8 Hz, 24H, N(*i*Pr-C*H*$_3$)) ppm. ^{13}C{^{1}H} NMR (126 MHz, C_6D_6, 297 K): δ = 200.2 ($C_{carbene}$, identified by HMBC), 149.0 (*i*-Ar*C*), 136.8 (*o*-Ar*C*), 128.2 (*m*-Ar*C*), 124.6 (*p*-Ar*C*), 67.7 (N*C*(CH_3)), 55.5 (*C*H_2), 46.7 (N(*i*Pr-*C*H)), 46.6 (*C*(CH_3)), 34.1 (C(*C*H_3)), 29.5 (NC(*C*H_3)), 29.1 (*i*Pr-*C*H), 27.3 (*i*Pr-*C*H_3), 24.0 (*i*Pr-*C*H_3), 23.7 (N(*i*Pr-*C*H_3)) ppm. *Note: The C_{CN} resonance was not detected due to broadening caused by quadrupolar coupling with the $^{10/11}B$ nucleus.* ^{11}B NMR (160 MHz, C_6D_6, 297 K): δ = 22.2 (*B*(N*i*$Pr_2)_2$), –14.4 (d, $^1J_{B–H}$ = 119.6 Hz, *B*H) ppm. HRMS LIFDI for [$C_{33}H_{60}B_2N_4$] = [M]: calcd. 534.4999; found 534.4999. UV-vis (hexane, 25 °C): λ^1_{max} = 423 nm, λ^2_{max} = 403 nm.

(CAAC^{Cy})BH(CN–B(N*i*Pr$_2$)$_2$), 5b^{NiPr2}

To a solution of **1b** (100 mg, 171 µmol) in benzene (1 mL) (*i*$Pr_2N)_2$BCl (76.0 mg, 308 µmol, 1.80 equiv.) was added. The reaction mixture was heated at 80 °C overnight, whereupon a colorless precipitate formed and the solution turned bright orange. After removal of all volatiles *in vacuo* the residue was extracted with hexane. Slow evaporation of the hexane solution yielded orange single crystals, which were washed with one portion of hexane (0.3 mL) and

dried *in vacuo* to yield **5b$^{\mathbf{N}\boldsymbol{i}\mathbf{Pr2}}$** (66.8 mg, 116 µmol, 68%). ^{1}H{^{11}B} NMR (500 MHz, C_6D_6, 297 K): δ = 7.23–7.16 (m, 3H, *p*-Ar*H*, *m*-Ar*H*), 3.40 (sept, ^{3}J = 6.7 Hz, 4H, N(*i*Pr-C*H*)), 3.28 (sept, ^{3}J = 6.7 Hz, 2H, *i*Pr-C*H*), 2.35 (br s, 1H, B*H*), 2.34–2.27 (m, 2H, Cy-CH_2), 2.06–2.00 (m, 2H, Cy-CH_2), 1.95 (s, 2H, CH_2), 1.80–1.74 (m, 3H, Cy-CH_2), 1.54 (d, ^{3}J = 6.7 Hz, 6H, *i*Pr-CH_3), 1.53–1.46 (m, 3H, Cy-CH_2), 1.32 (d, ^{3}J = 6.7 Hz, 6H, *i*Pr-CH_3), 1.15 (s, 6H, NC(CH_3)), 1.13 (d, ^{3}J = 6.7 Hz, 24H, N(*i*Pr-CH_3)) ppm. ^{13}C{^{1}H} NMR (126 MHz, C_6D_6, 297 K): δ = 201.5 ($C_{carbene}$, identified by HMBC), 149.0 (*i*-Ar*C*), 136.8 (*o*-Ar*C*), 128.1 (*m*-Ar*C*), 124.6 (*p*-ArC), 67.8 (N*C*(CH$_3$)), 51.5 (*C*(C_5H_{10})), 49.0 (*C*H$_2$), 46.7 (N(*i*Pr-*C*H)), 41.8 (Cy-*C*H$_2$), 29.8 (NC(*C*H$_3$)), 29.1 (*i*Pr-*C*H), 27.3 (*i*Pr-*C*H$_3$), 25.8 (Cy-*C*H$_2$), 24.1 (Cy-*C*H$_2$), 24.0 (*i*Pr-*C*H$_3$), 23.6 (N(*i*Pr-*C*H$_3$)) ppm. *Note: The C_{CN} resonance was not detected due to broadening caused by quadrupolar coupling with the $^{10/11}$B nucleus.* ^{11}B NMR (160 MHz, C_6D_6, 297 K): δ = 22.4 (*B*(N*i*Pr$_2$)$_2$), –14.2 (d, $^{1}J_{B–H}$ = 114.3 Hz, *B*H) ppm. HRMS LIFDI for [$C_{36}H_{64}B_2N_4$] = [M]: calcd. 574.5304; found 574.5312. UV-vis (hexane, 25 °C): λ^{1}_{max} = 429 nm, λ^{2}_{max} = 401 nm.

NMR spectra of isolated compounds

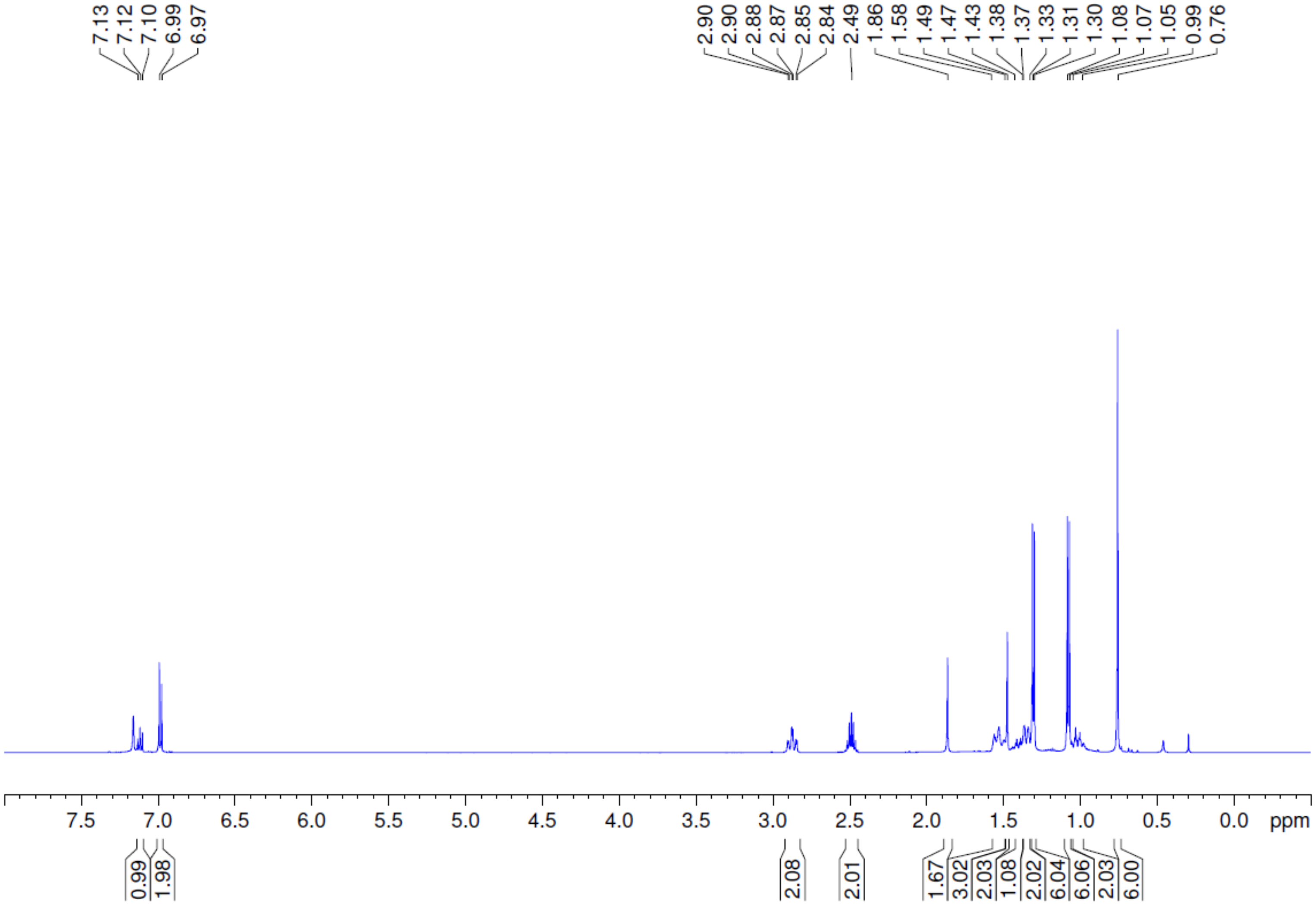

Figure S1. $^{1}H\{^{11}B\}$ NMR spectrum of **1b** in C_6D_6.

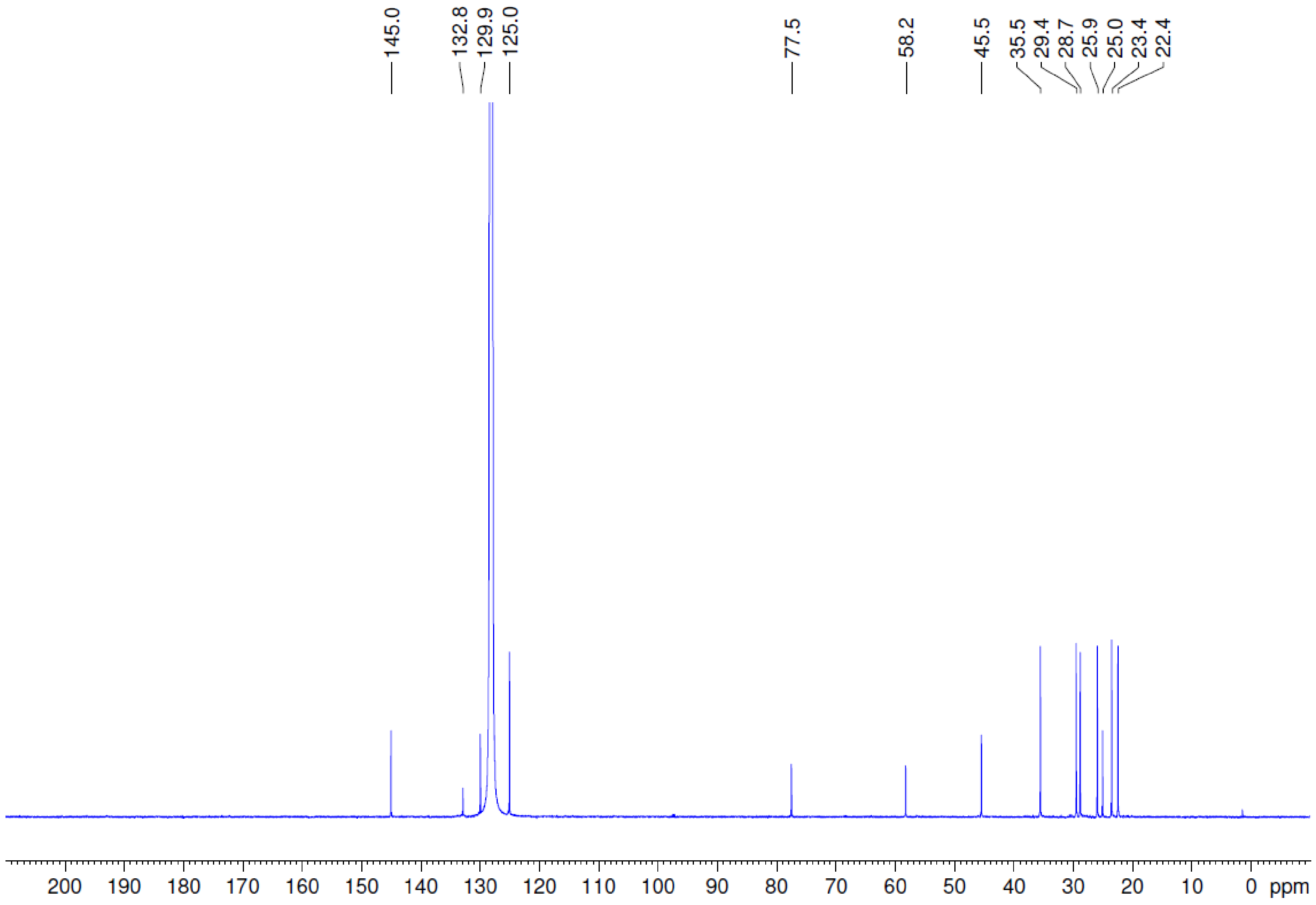

Figure S2. $^{13}C\{^{1}H\}$ NMR spectrum of **1b** in C_6D_6.

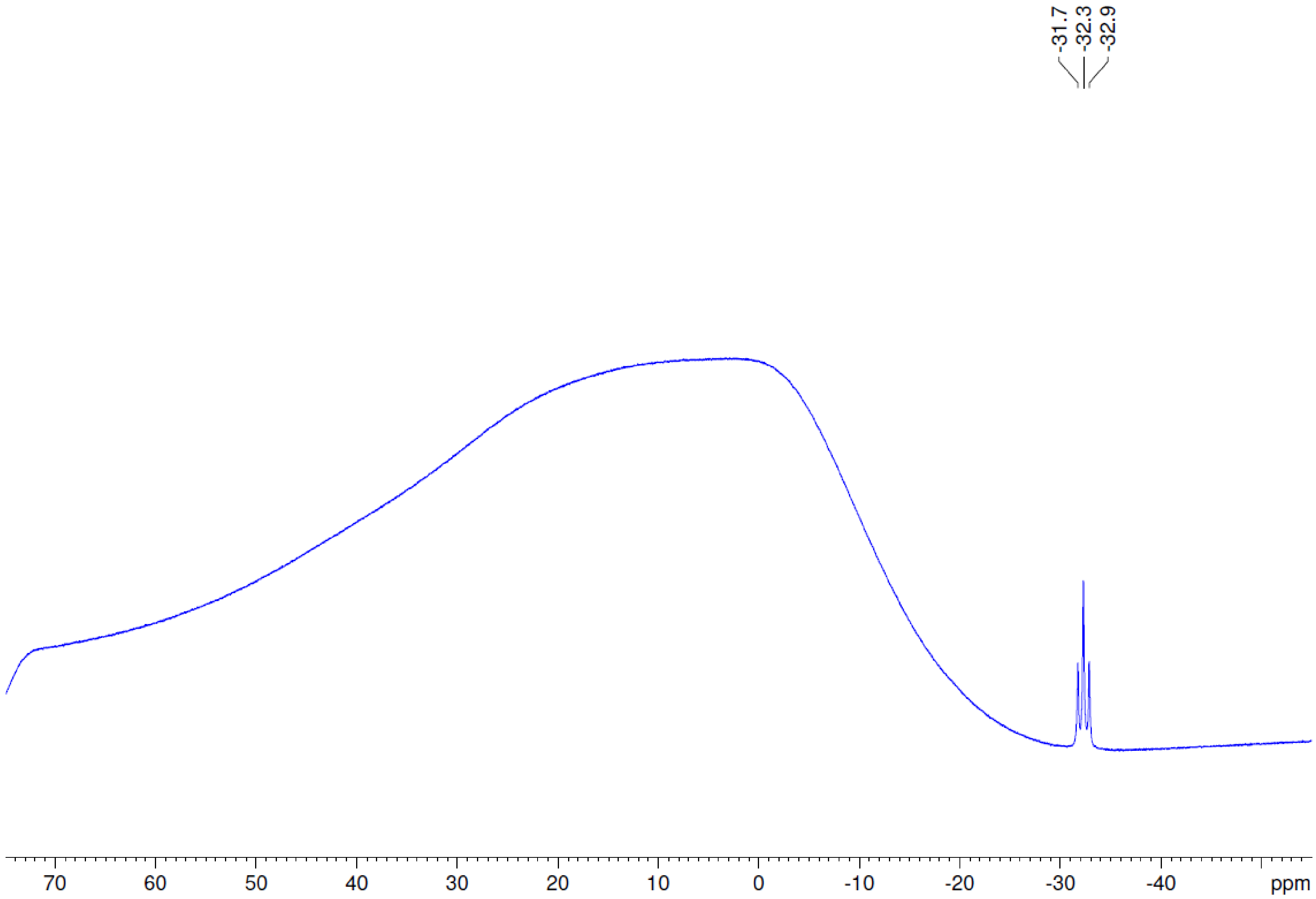

Figure S3. ^{11}B NMR spectrum of **1b** in C_6D_6.

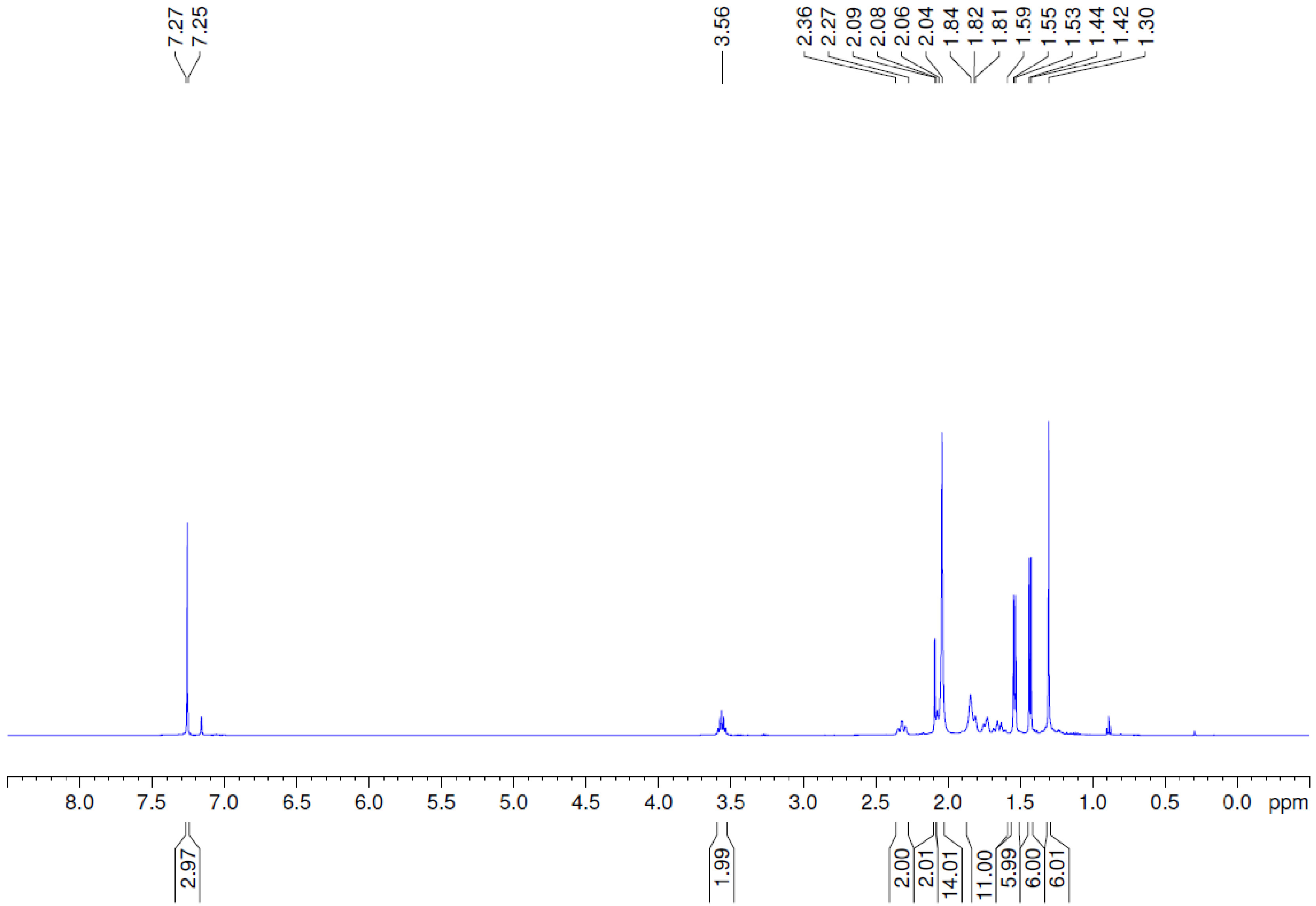

Figure S4. $^1H\{^{11}B\}$ NMR spectrum of **2b-TMEDA** in C_6D_6. The additional resonance at 0.89 ppm corresponds to residual hexane.

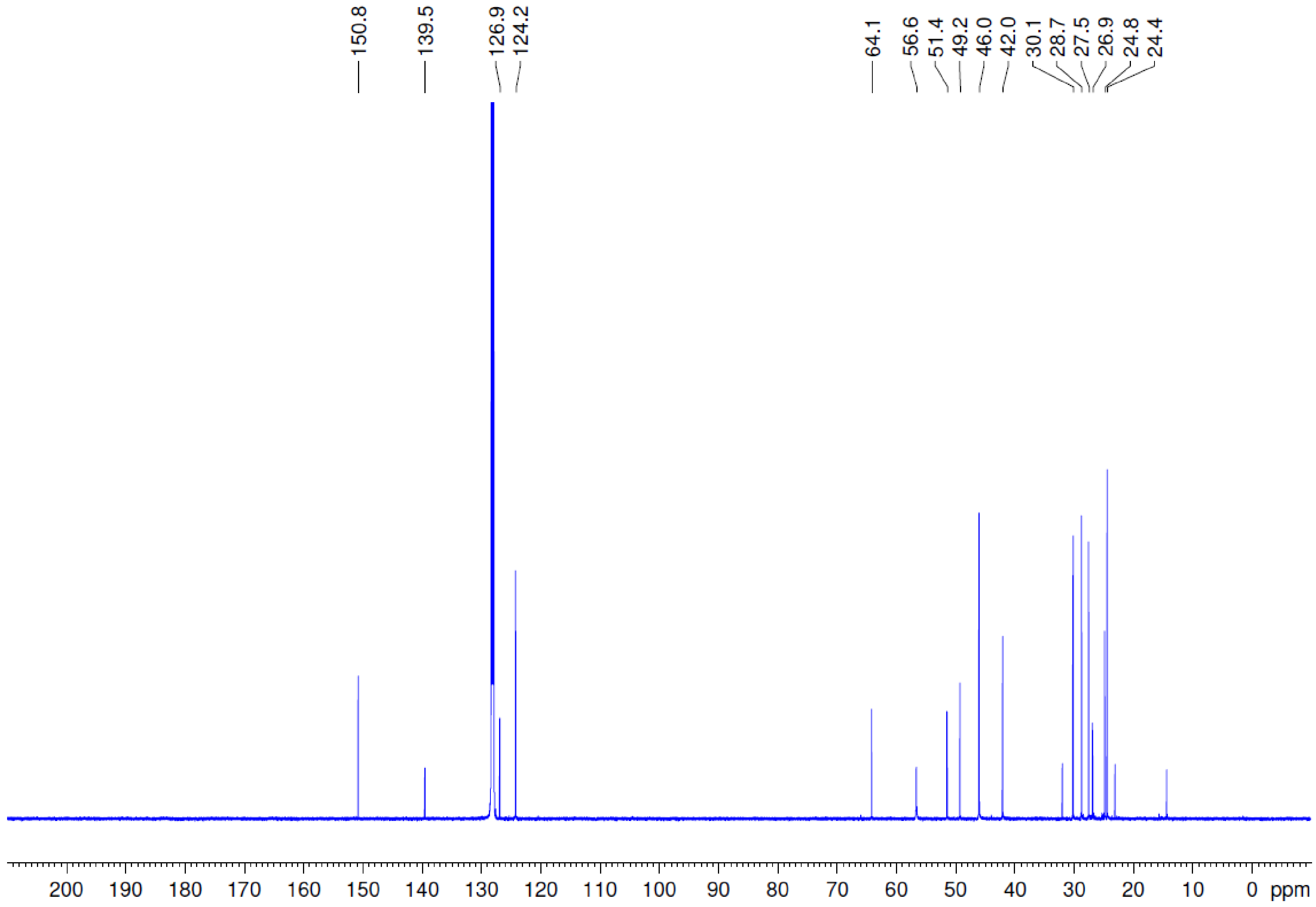

Figure S5. $^{13}C\{^1H\}$ NMR spectrum of **2b-TMEDA** in C_6D_6. The additional resonances at 14.3, 23.0 and 31.9 ppm correspond to residual hexane.

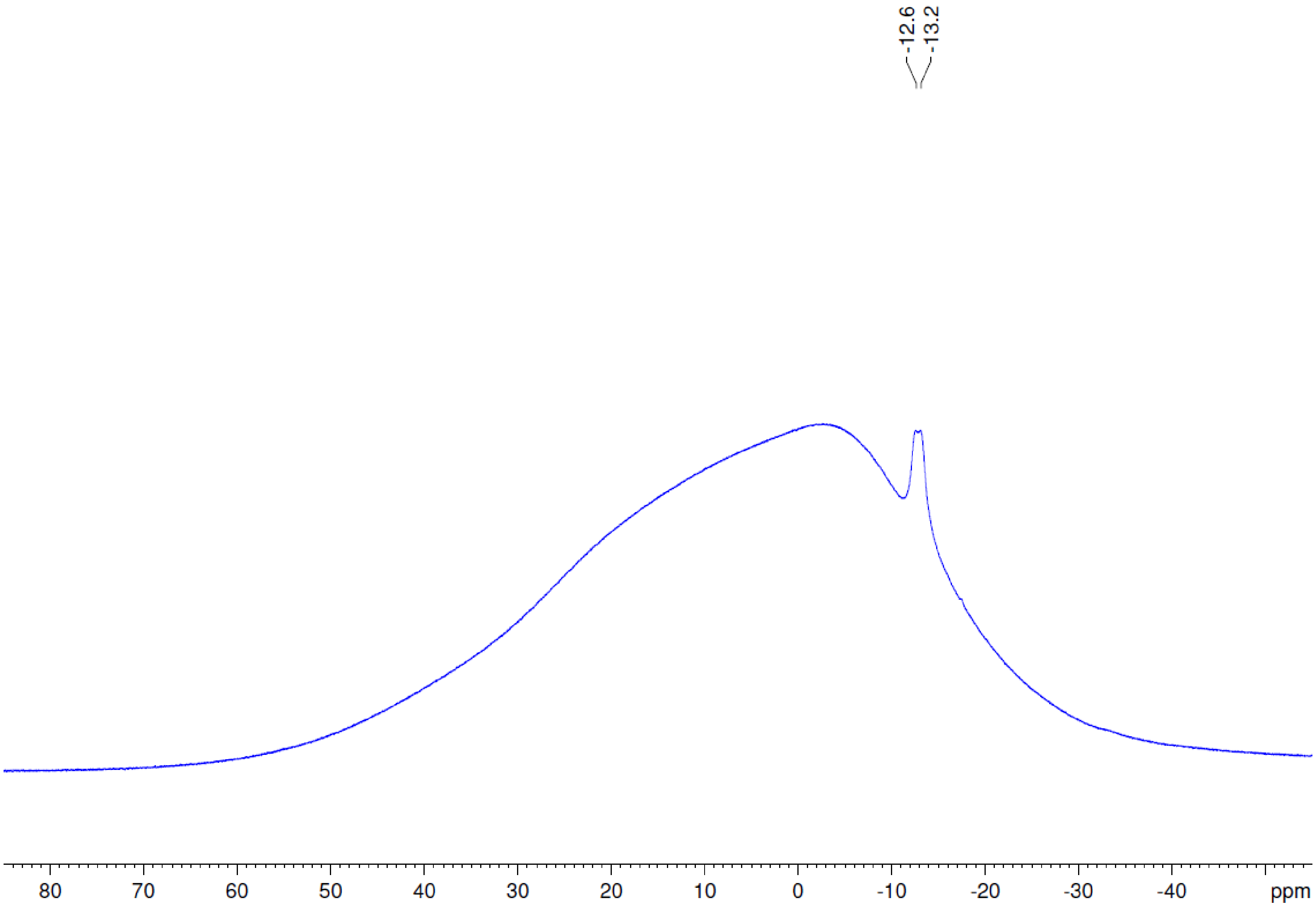

Figure S6. ^{11}B NMR spectrum of **2b-TMEDA** in C_6D_6.

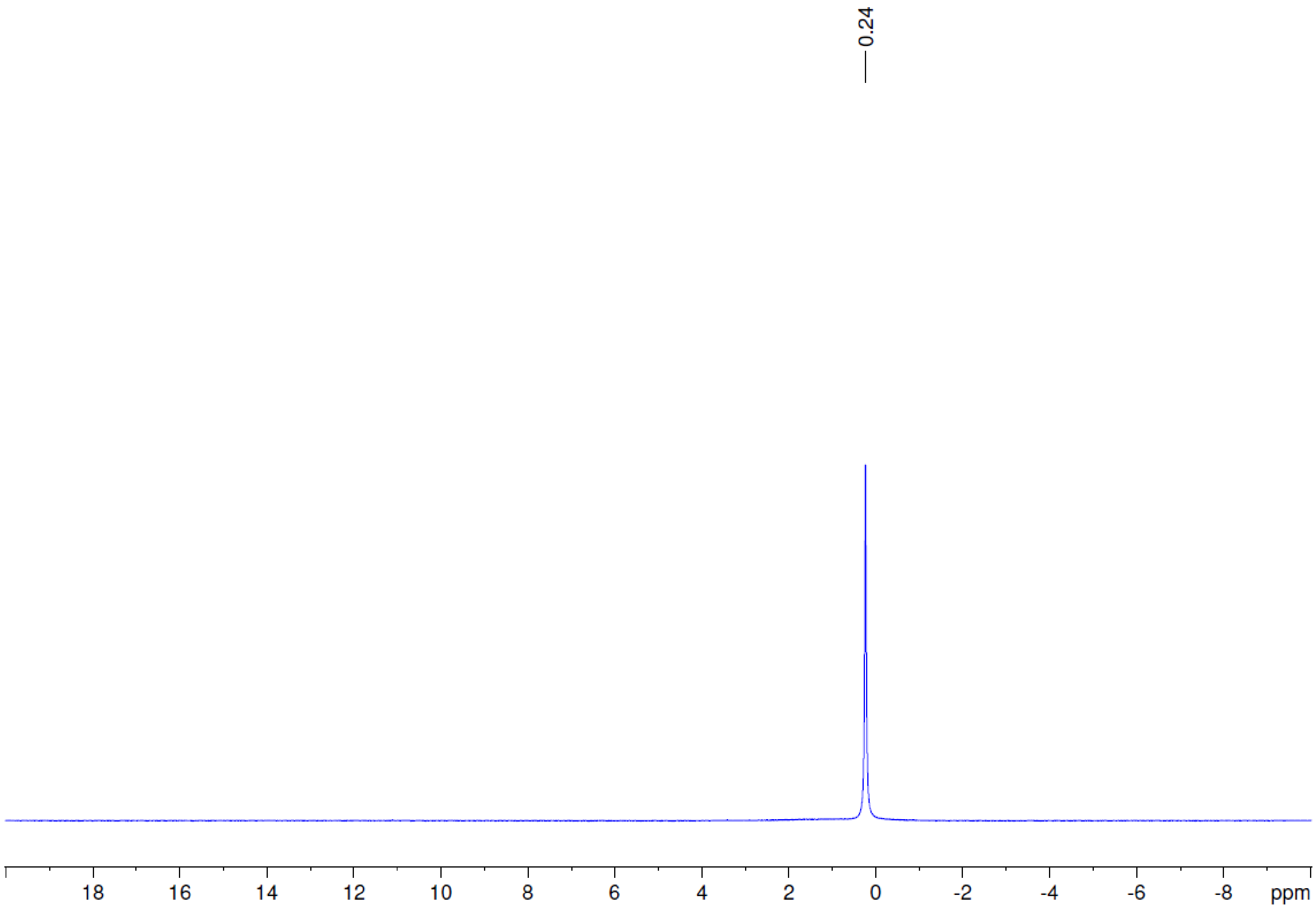

Figure S7. ^{7}Li NMR spectrum of **2b-TMEDA** in C_6D_6.

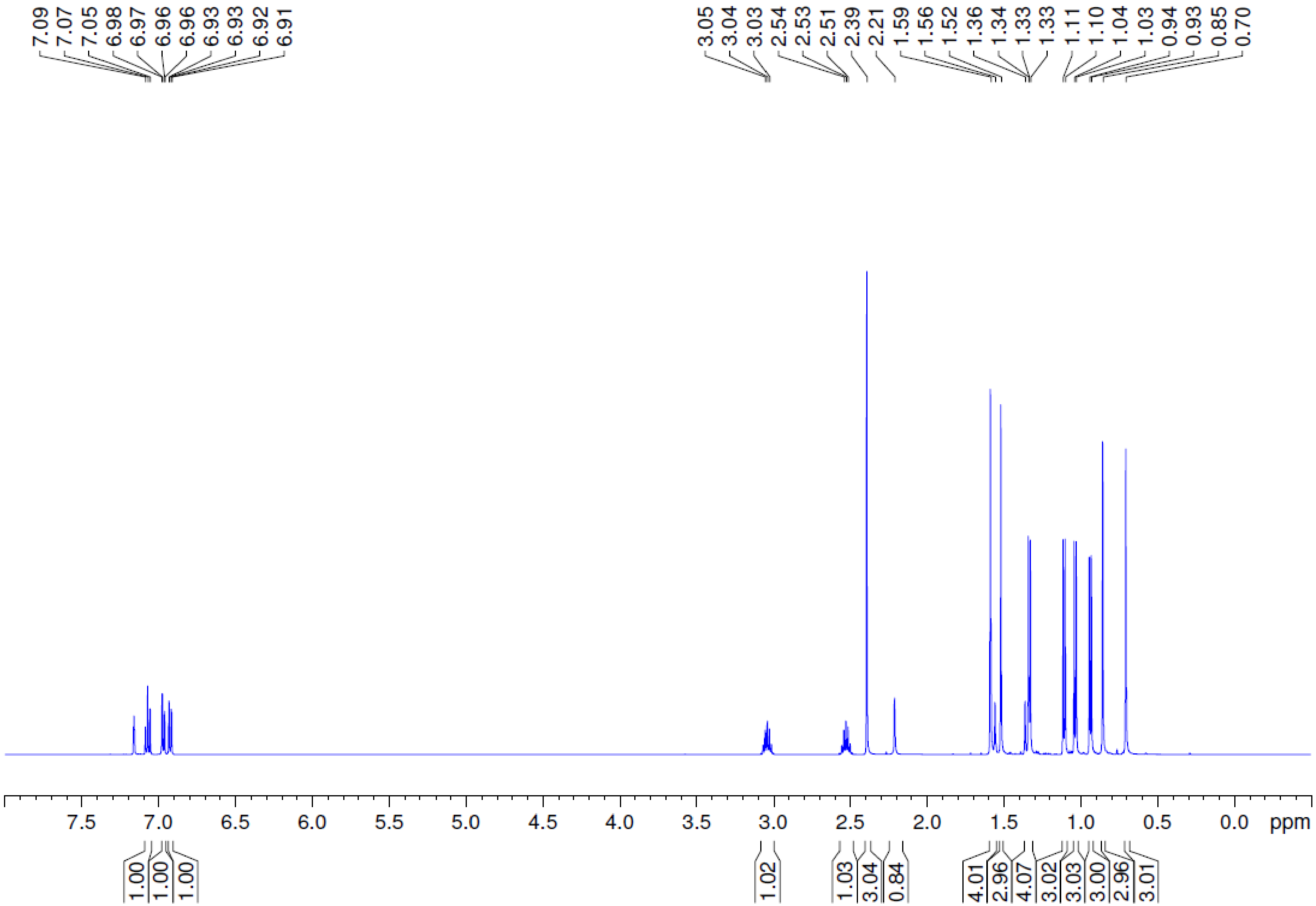

Figure S8. $^{1}H\{^{11}B\}$ NMR spectrum of **4a$^{\mathbf{COMe}}$** in C_6D_6.

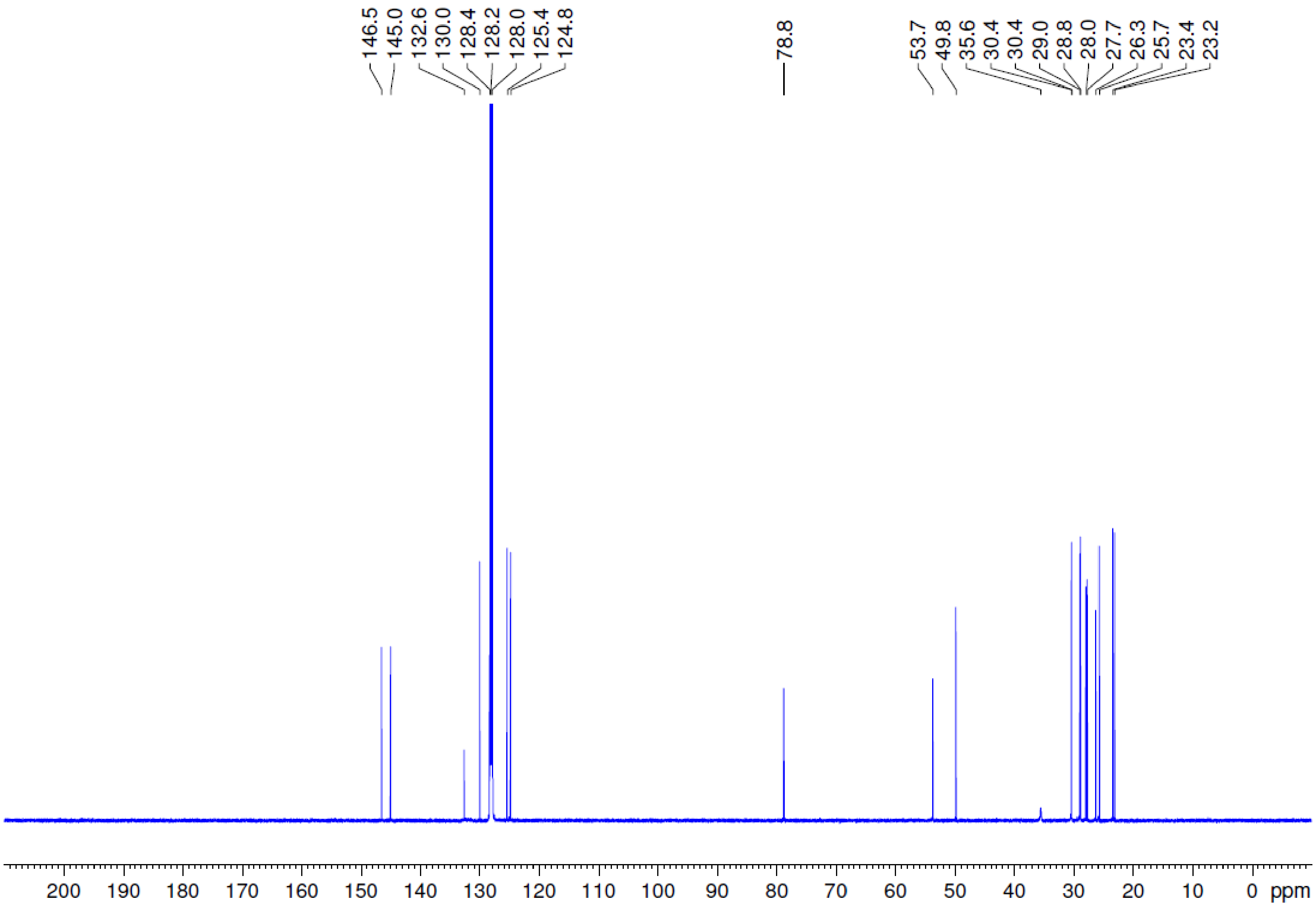

Figure S9. $^{13}C\{^{1}H\}$ NMR spectrum of **4a**$^{\mathbf{COMe}}$ in C_6D_6.

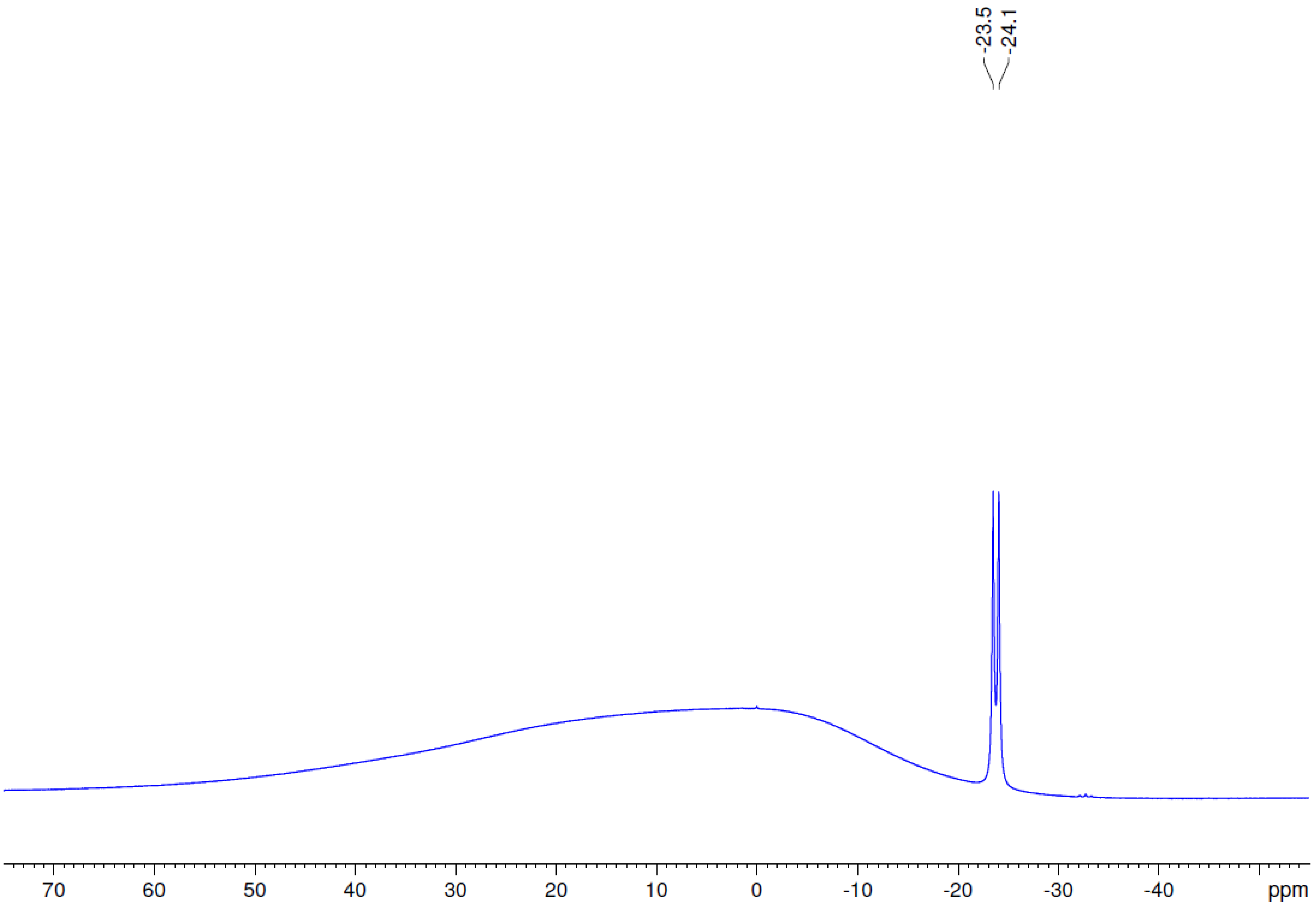

Figure S10. ^{11}B NMR spectrum of **4a**$^{\mathbf{COMe}}$ in C_6D_6.

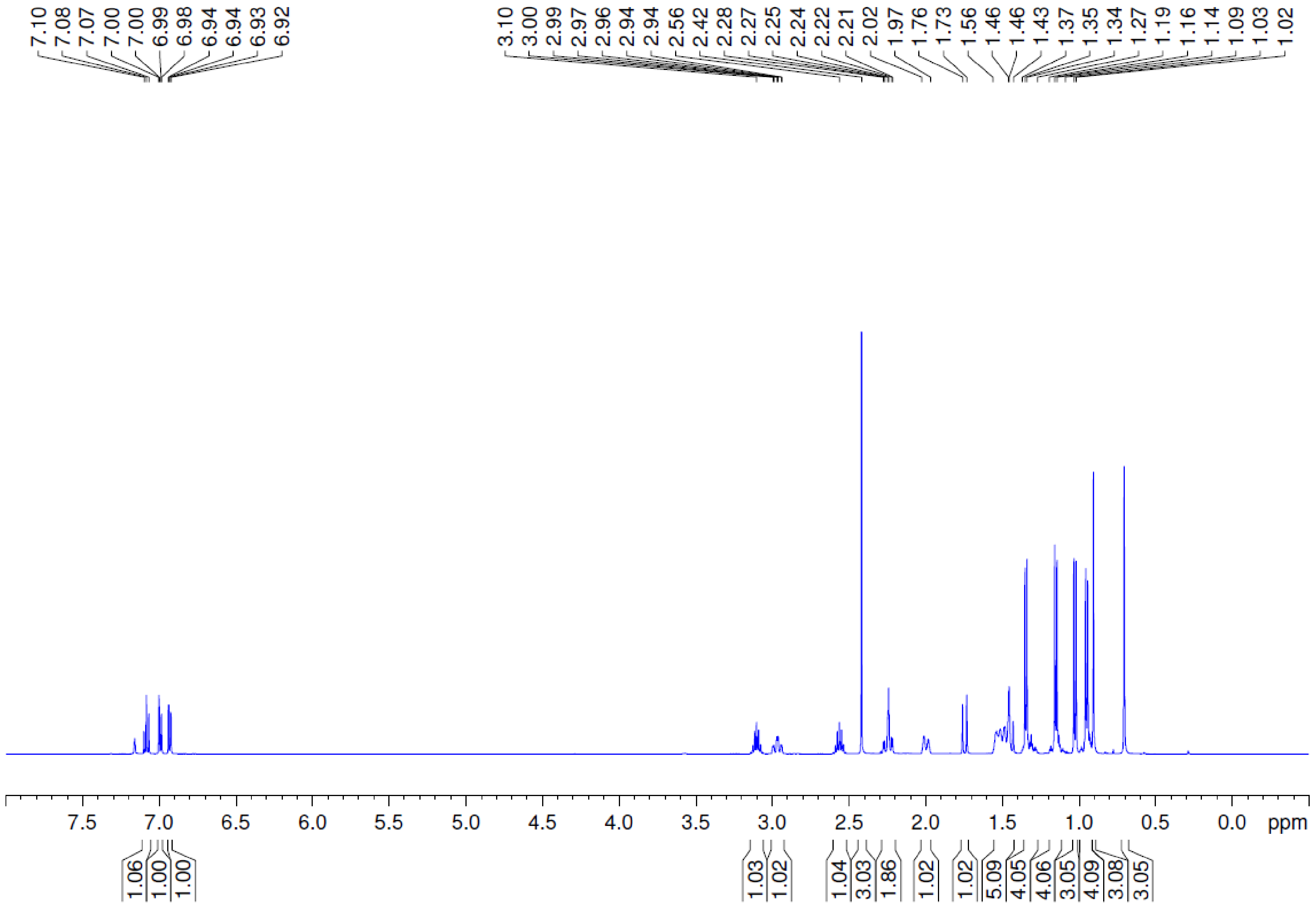

Figure S11. $^{1}H\{^{11}B\}$ NMR spectrum of **4b**$^{\mathbf{COMe}}$ in C_6D_6.

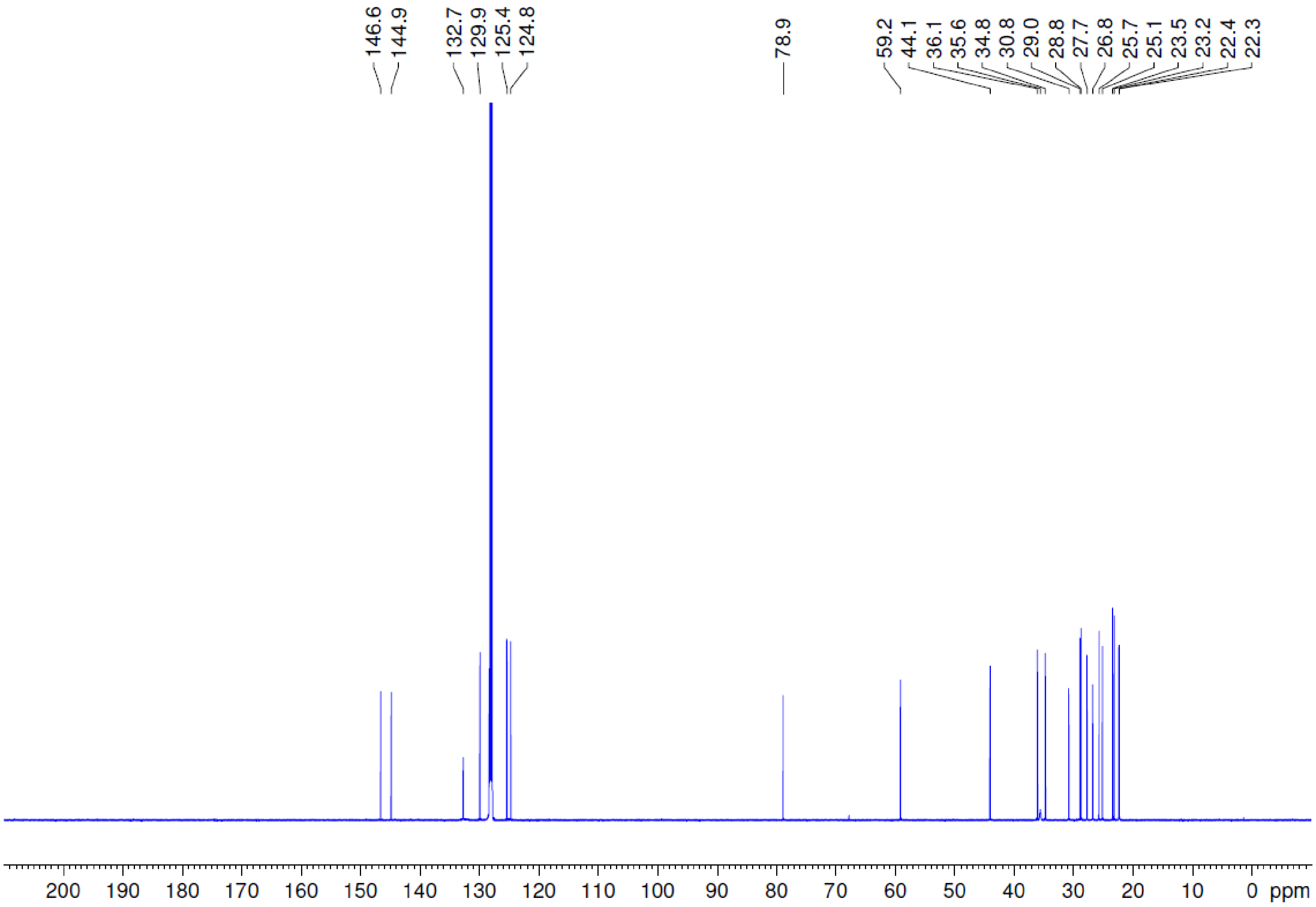

Figure S12. $^{13}C\{^1H\}$ NMR spectrum of **4b^{COMe}** in C_6D_6.

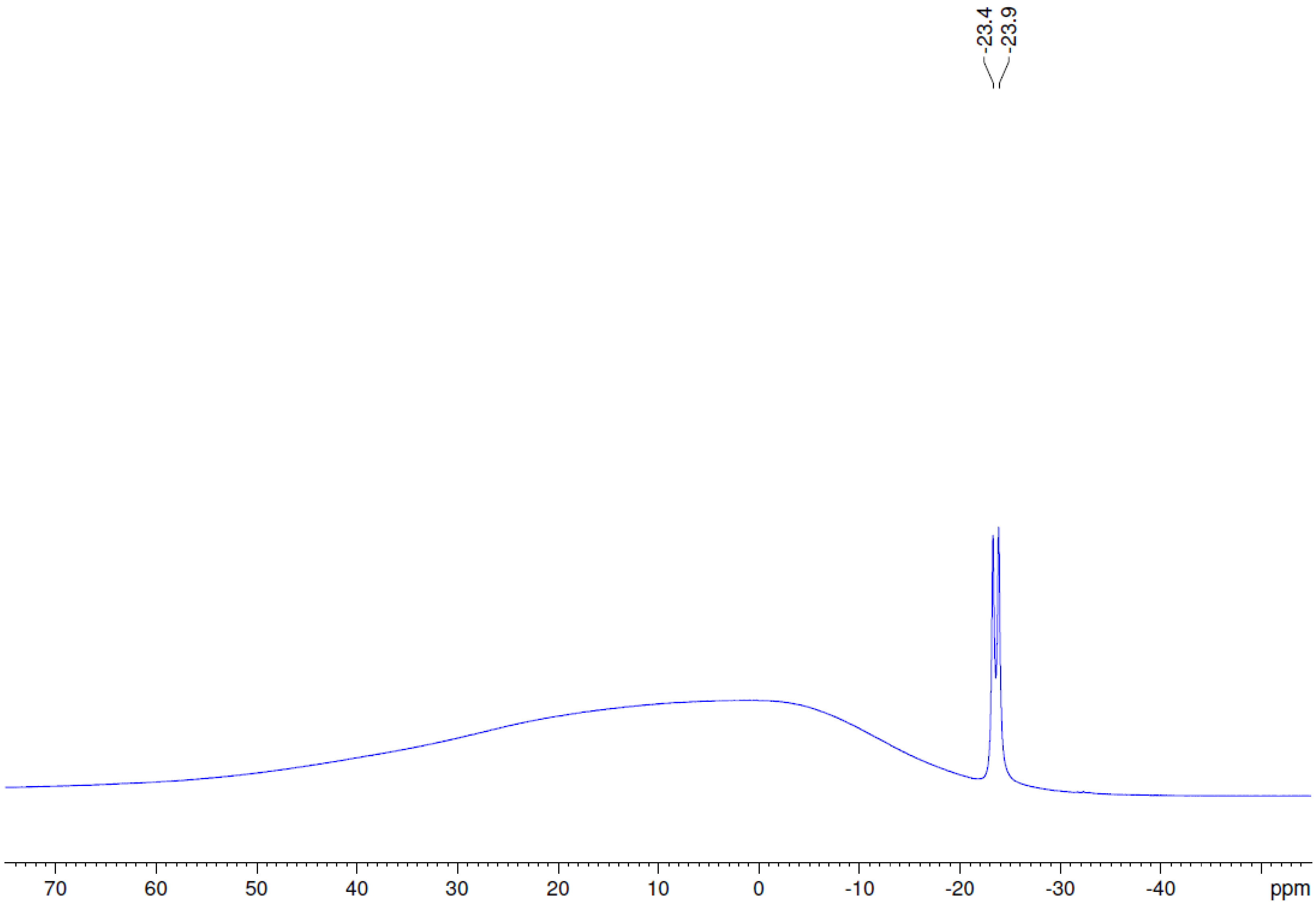

Figure S13. ^{11}B NMR spectrum of **4b^{COMe}** in C_6D_6.

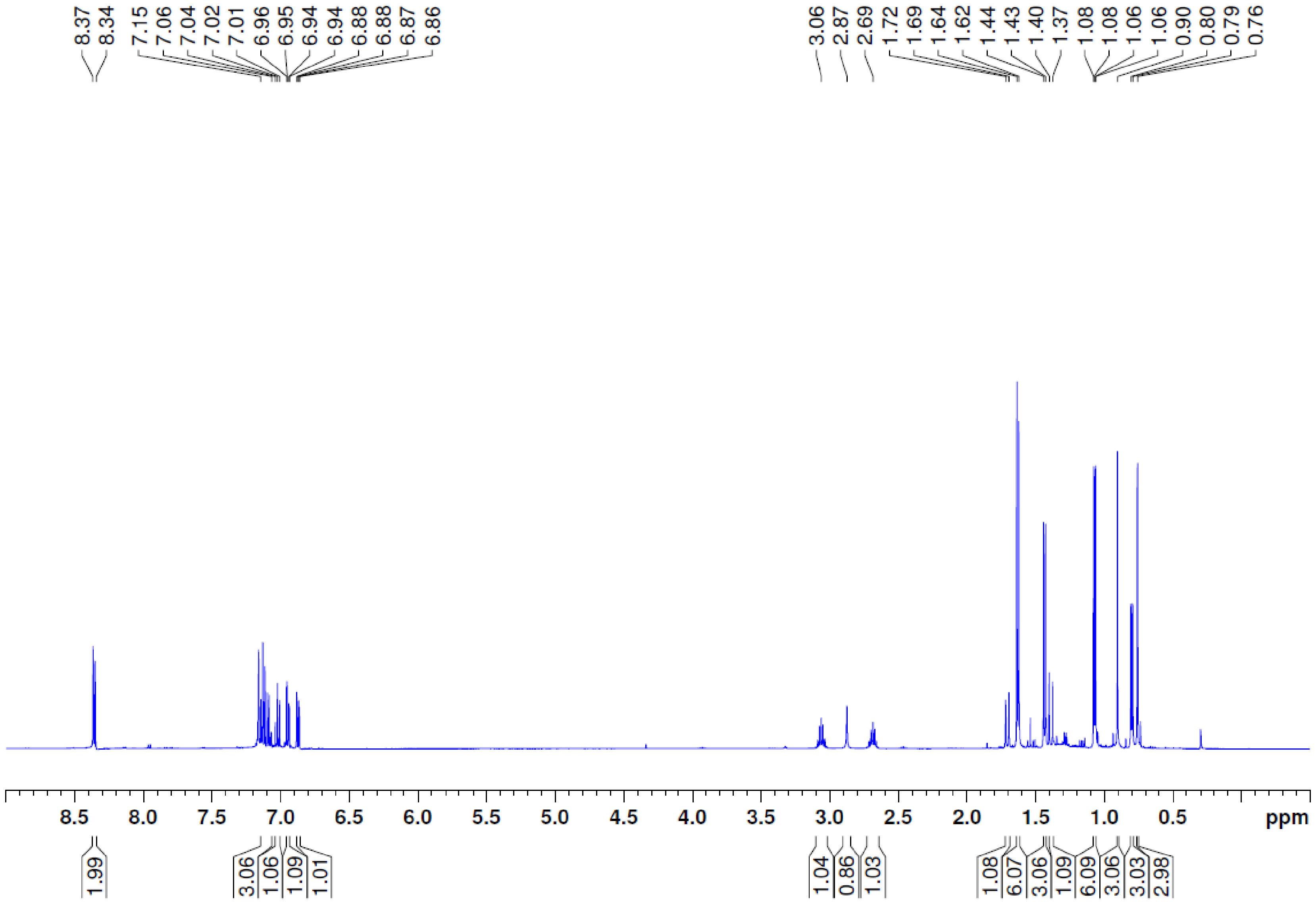

Figure S14. $^{1}H\{^{11}B\}$ NMR spectrum of **4a^{COPh}** in C_6D_6. The additional resonances correspond to **1a** (5%).

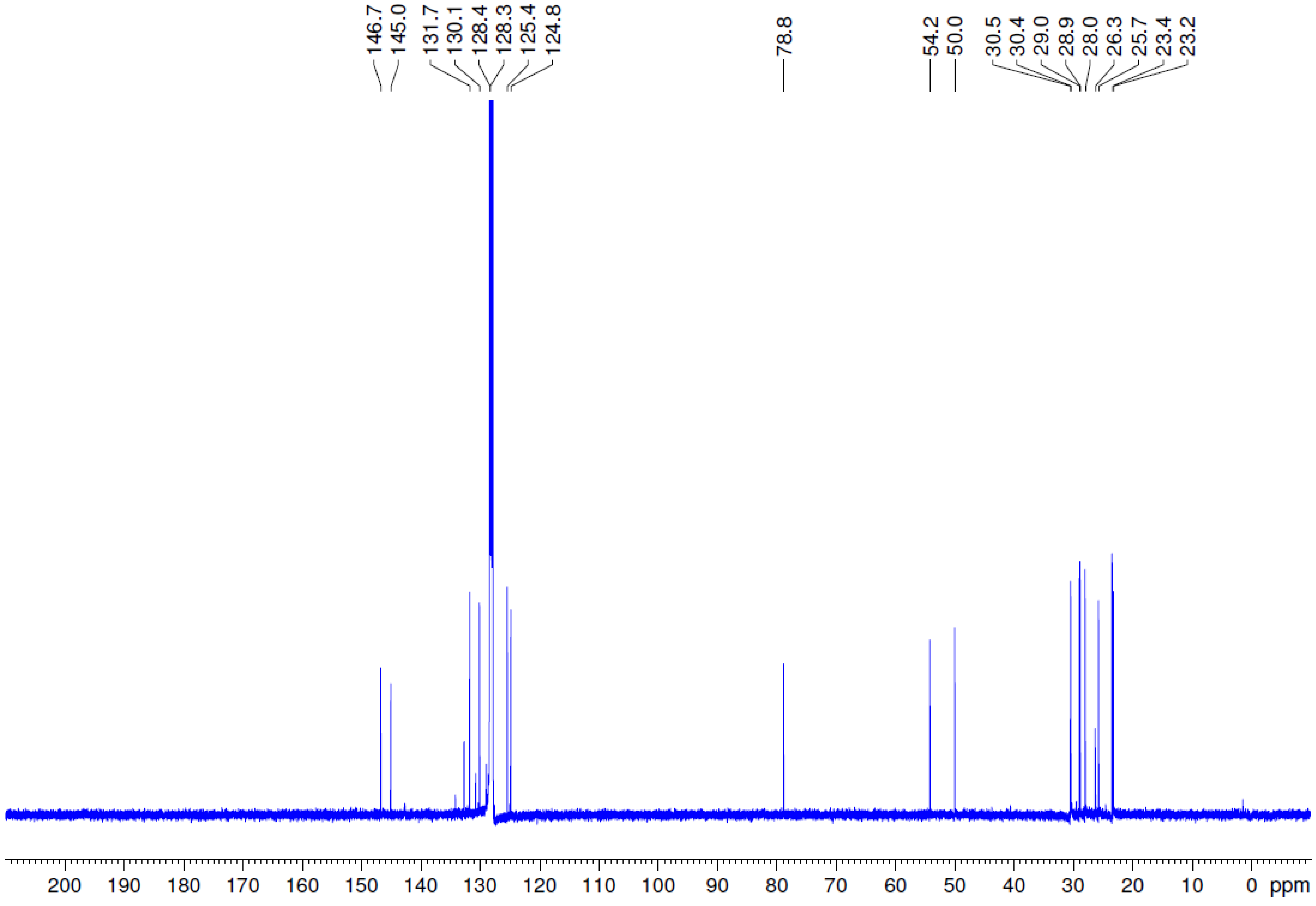

Figure S15. $^{13}C\{^1H\}$ NMR spectrum of $\mathbf{4a^{COPh}}$ in C_6D_6.

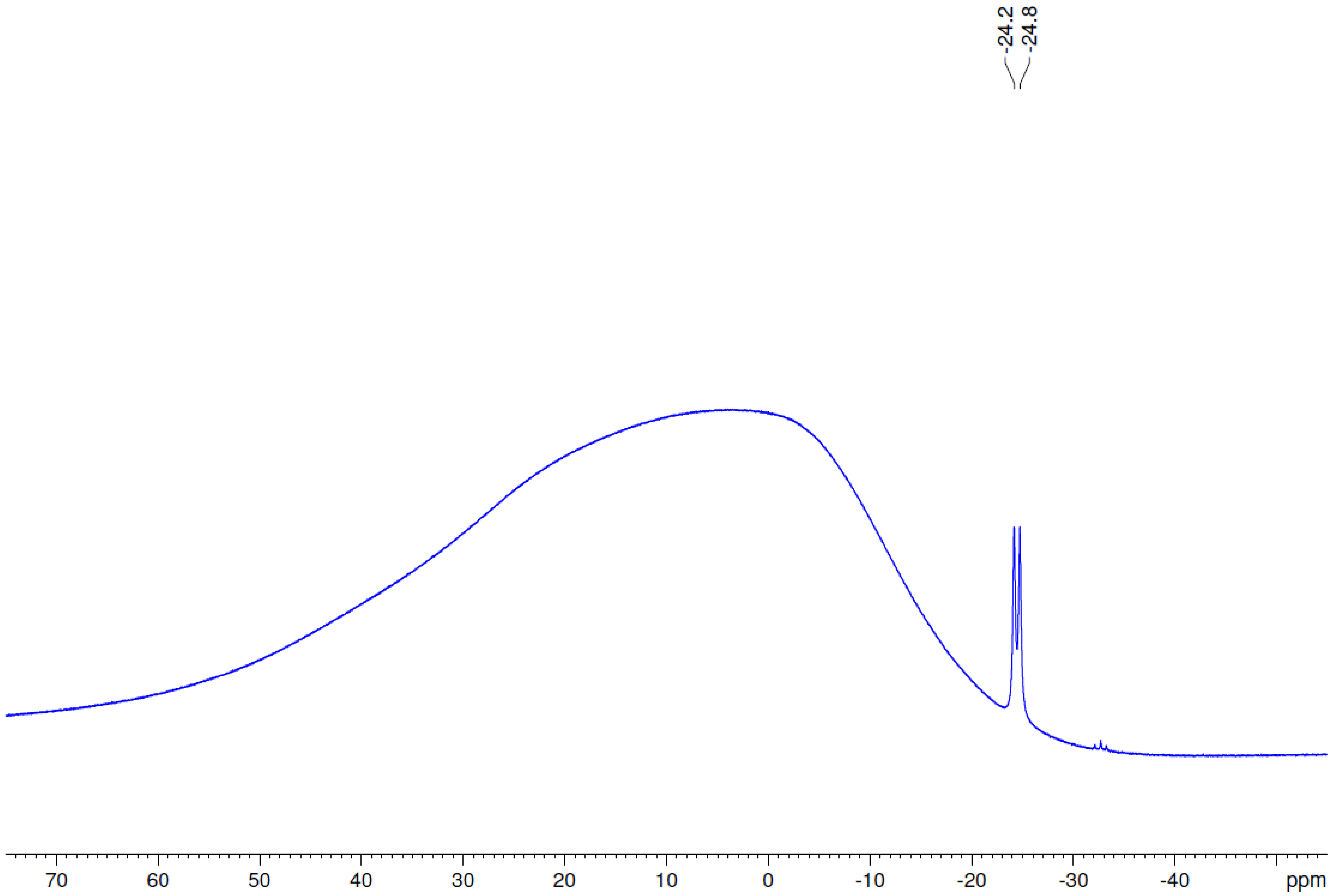

Figure S16. ^{11}B NMR spectrum of $\mathbf{4a^{COPh}}$ in C_6D_6. The additional resonance at –33.3 ppm corresponds to **1a** (5%).

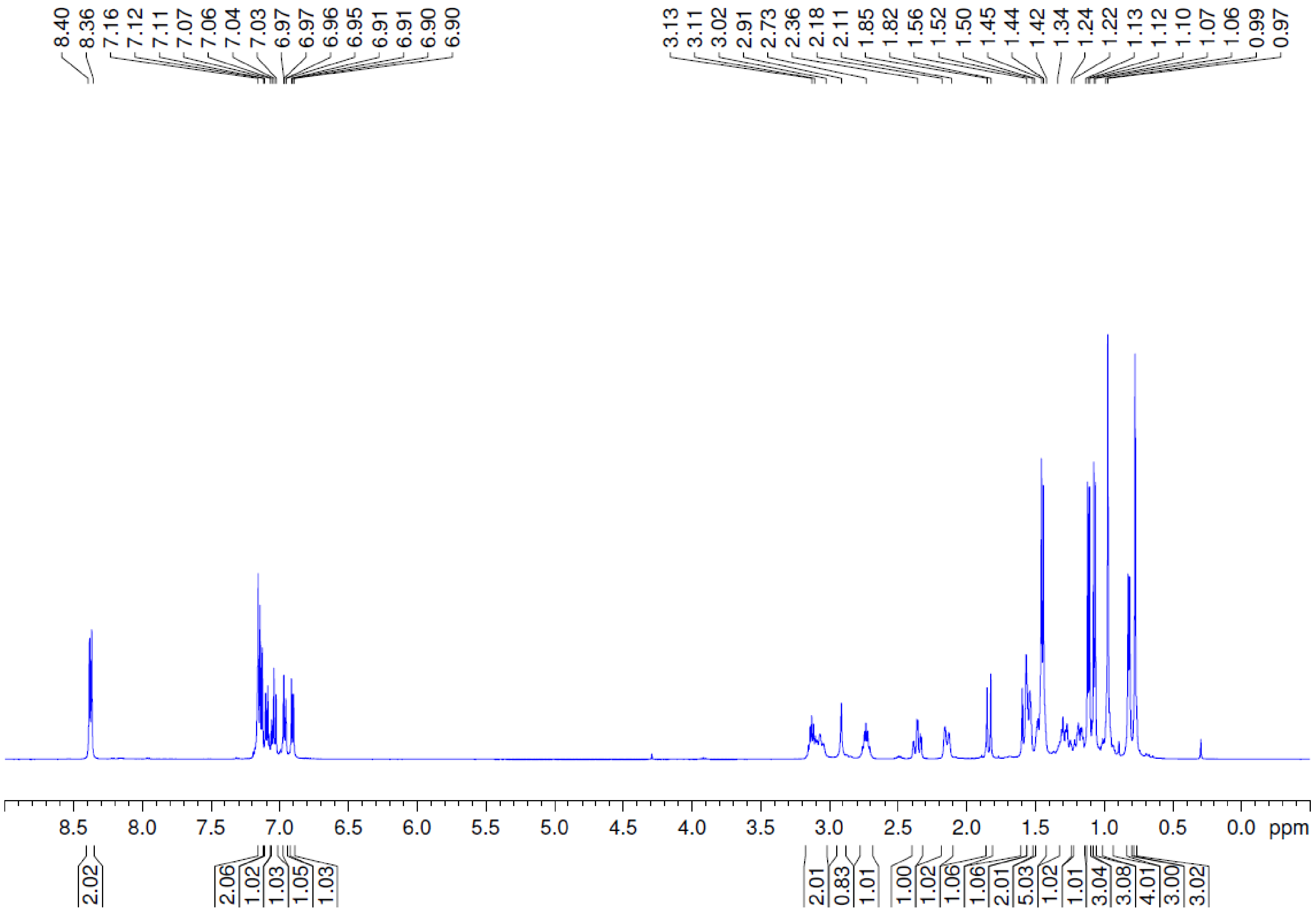

Figure S17. $^{1}H\{^{11}B\}$ NMR spectrum of **4b^{COPh}** in C_6D_6. The additional resonances correspond to **1b** (3%).

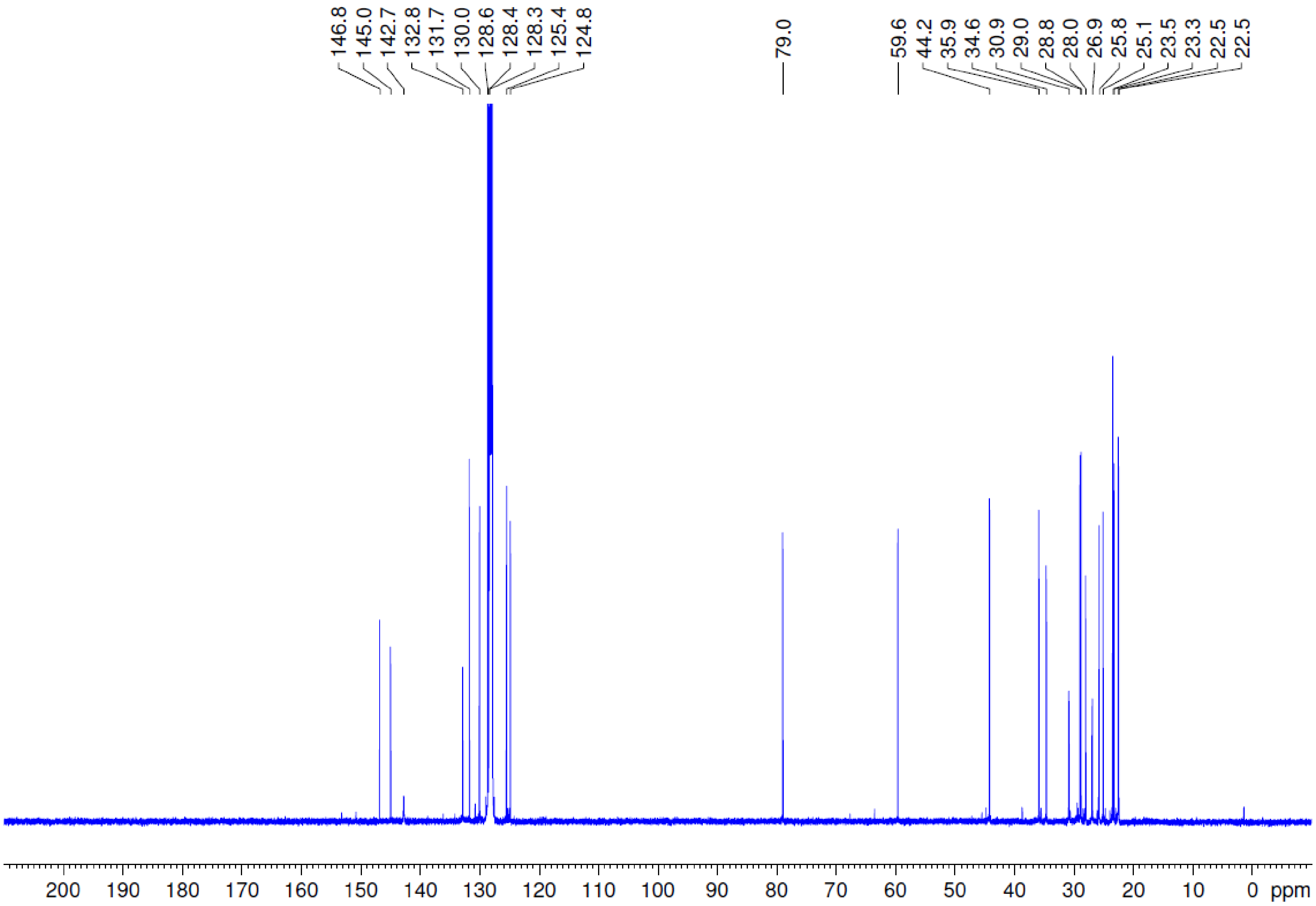

Figure S18. $^{13}C\{^{1}H\}$ NMR spectrum of $\mathbf{4b}^{\mathbf{COPh}}$ in C_6D_6.

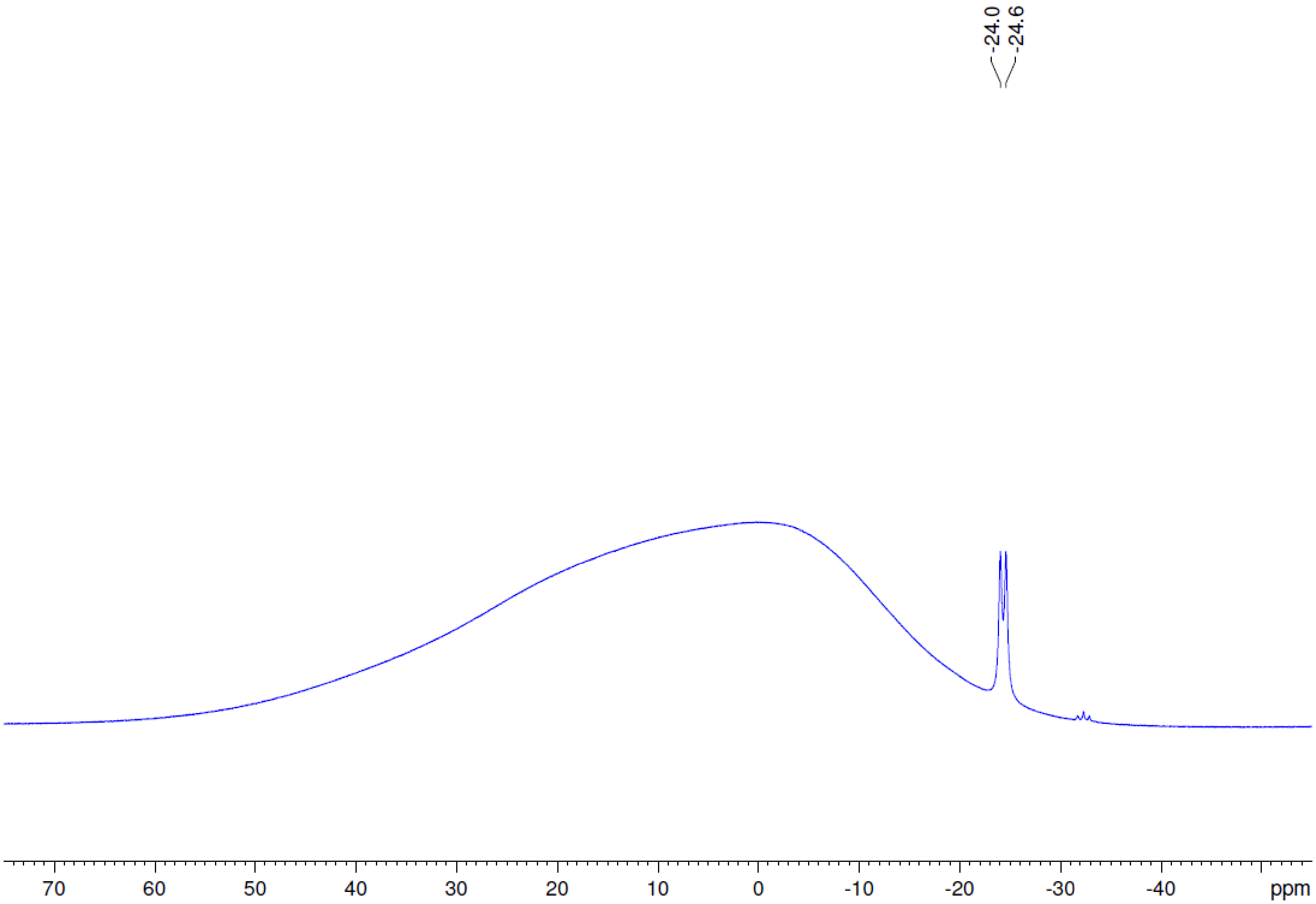

Figure S19. ^{11}B NMR spectrum of **4b^{COPh}** in C_6D_6. The additional resonance at –32.3 ppm corresponds to **1b** (3%).

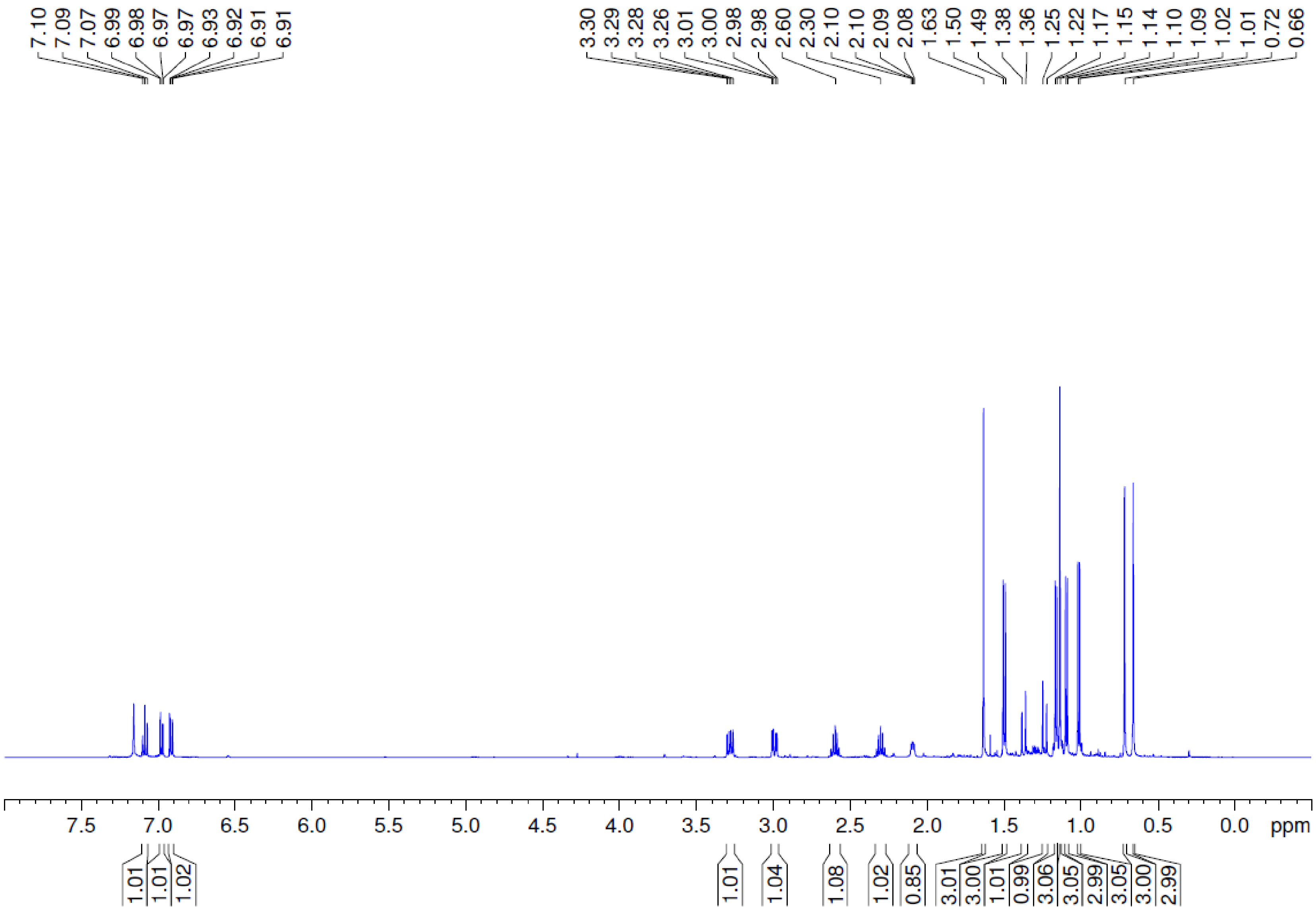

Figure S20. $^{1}H\{^{11}B\}$ NMR spectrum of **4a**$^{\mathbf{CH2Cl}}$ in C_6D_6. The additional resonances correspond to **1a** (1%).

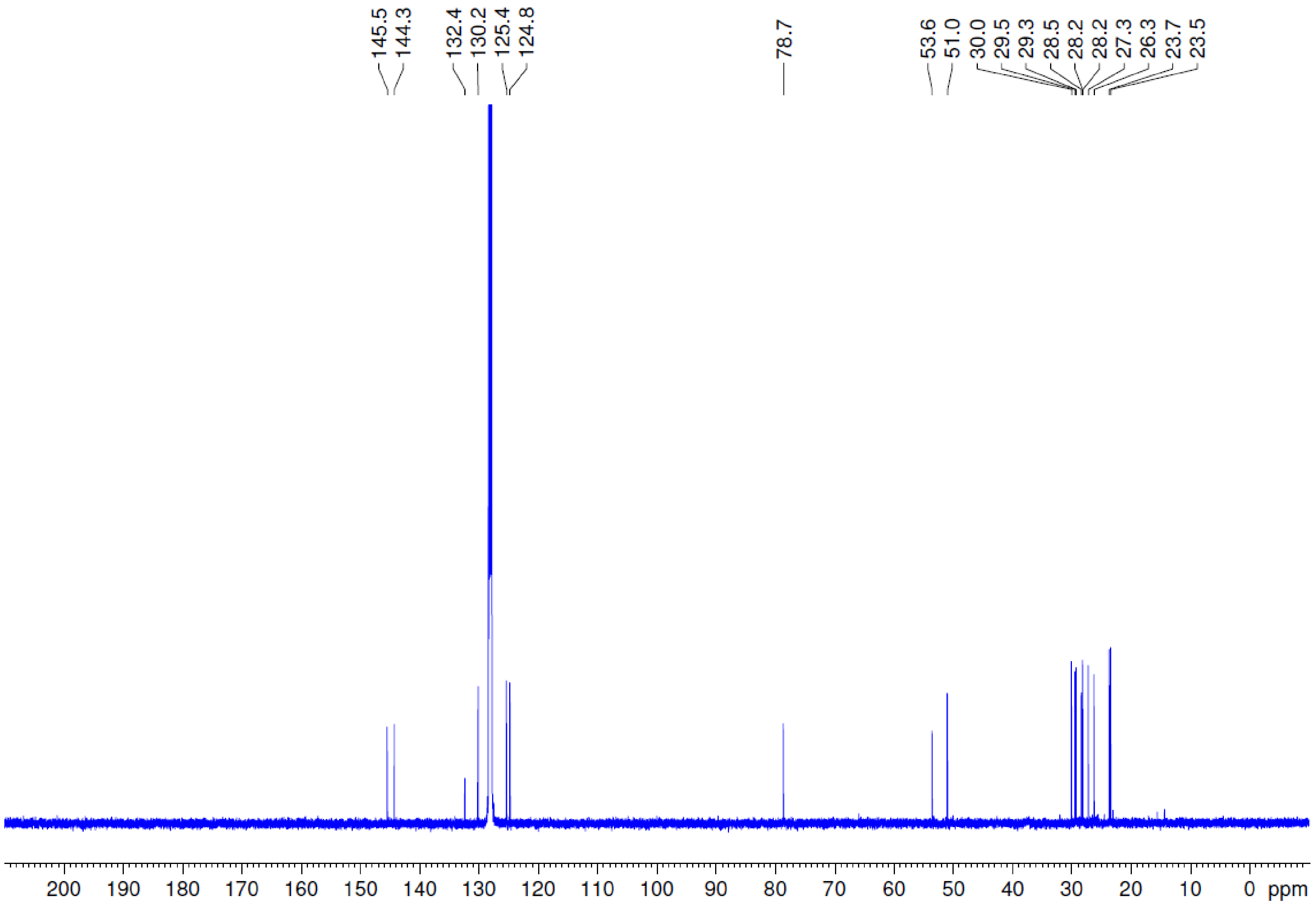

Figure S21. $^{13}C\{^{1}H\}$ NMR spectrum of $\mathbf{4a}^{\mathbf{CH2Cl}}$ in C_6D_6.

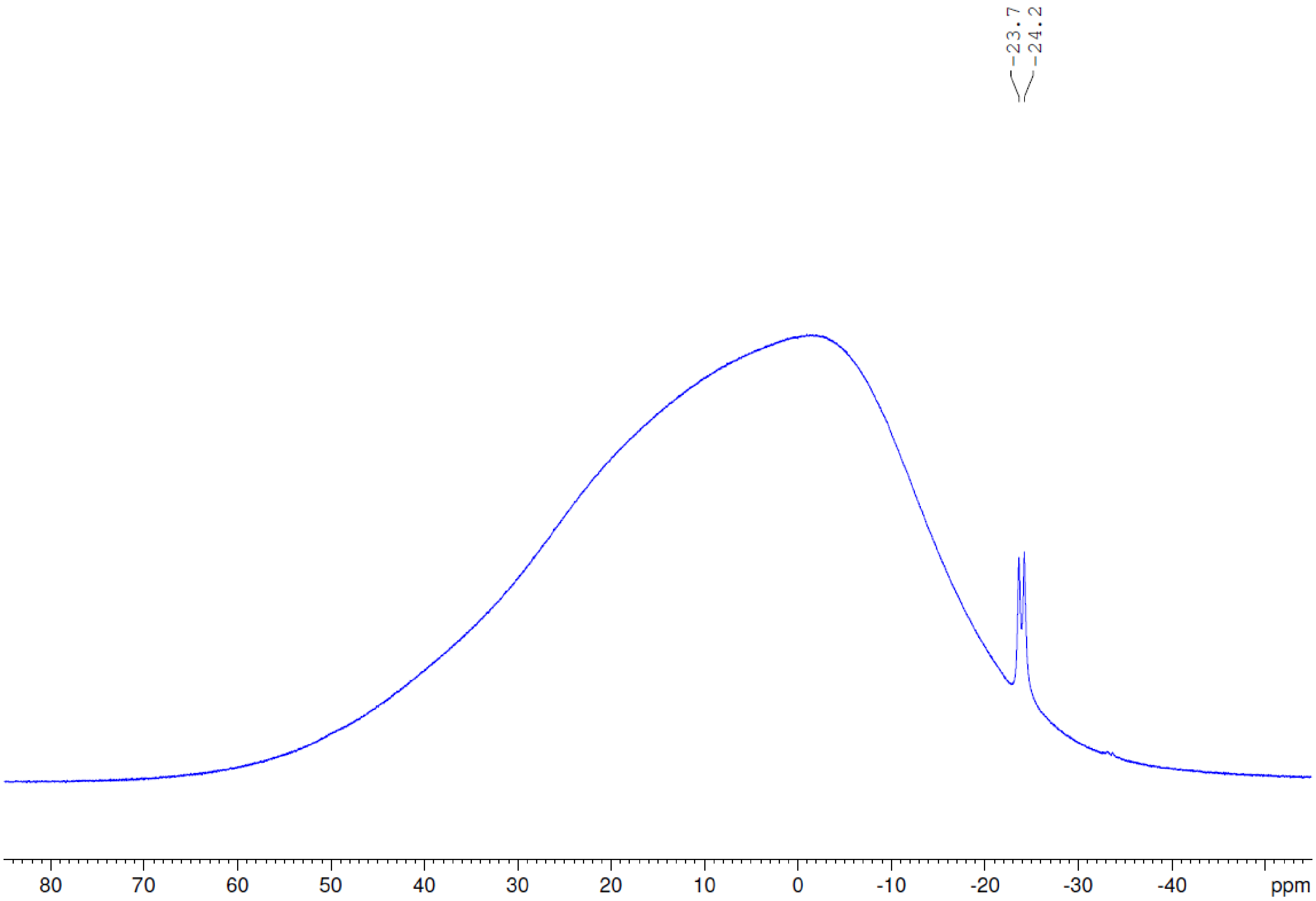

Figure S22. ^{11}B NMR spectrum of **4a^{CH2Cl}** in C_6D_6. The additional resonance at –33.3 ppm corresponds to **1a** (1%).

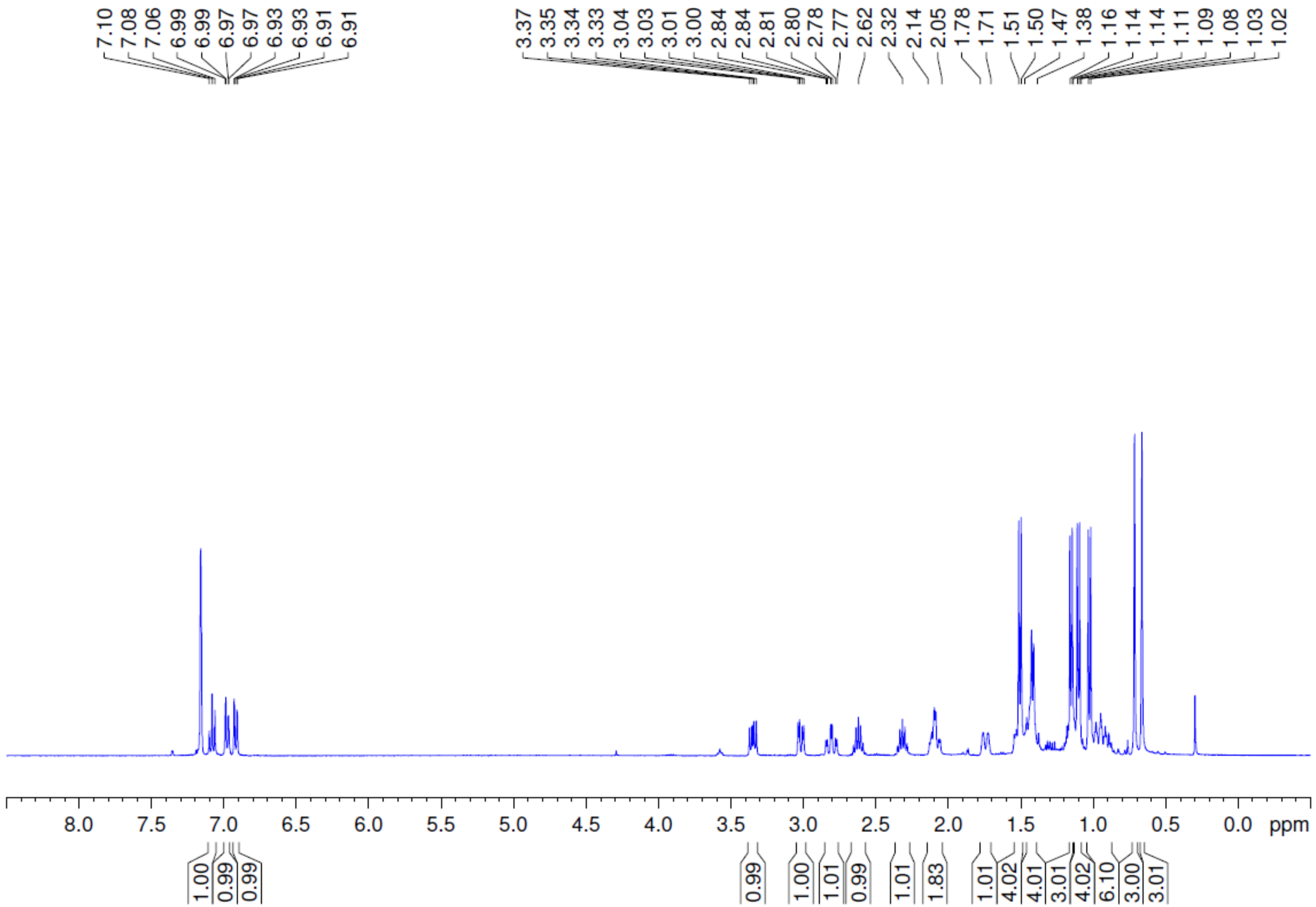

Figure S23. $^{1}H\{^{11}B\}$ NMR spectrum of **4b^{CH2Cl}** in C_6D_6. The additional resonance at 0.29 ppm corresponds to silicon grease and at 3.57 ppm to residual tetrahydrofuran. Other additional resonances correspond to **1b** (1%).

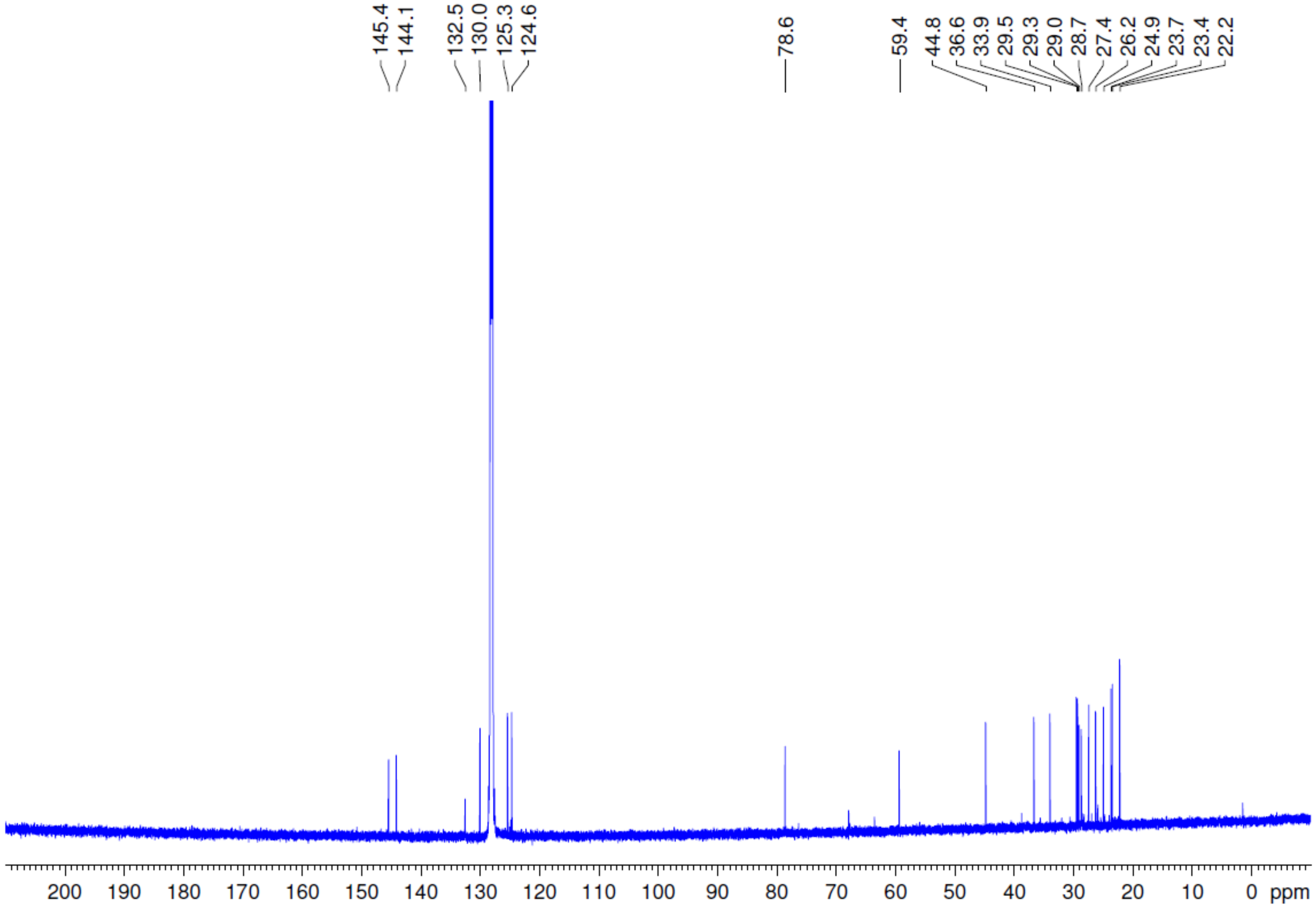

Figure S24. $^{13}C\{^{1}H\}$ NMR spectrum of $\mathbf{4b}^{\mathbf{CH2Cl}}$ in C_6D_6. The additional resonance at 1.38 ppm corresponds to silicon grease and at 21.1, 25.7 and 67.8 ppm to residual tetrahydrofuran. Other additional resonances correspond to **1b** (1%).

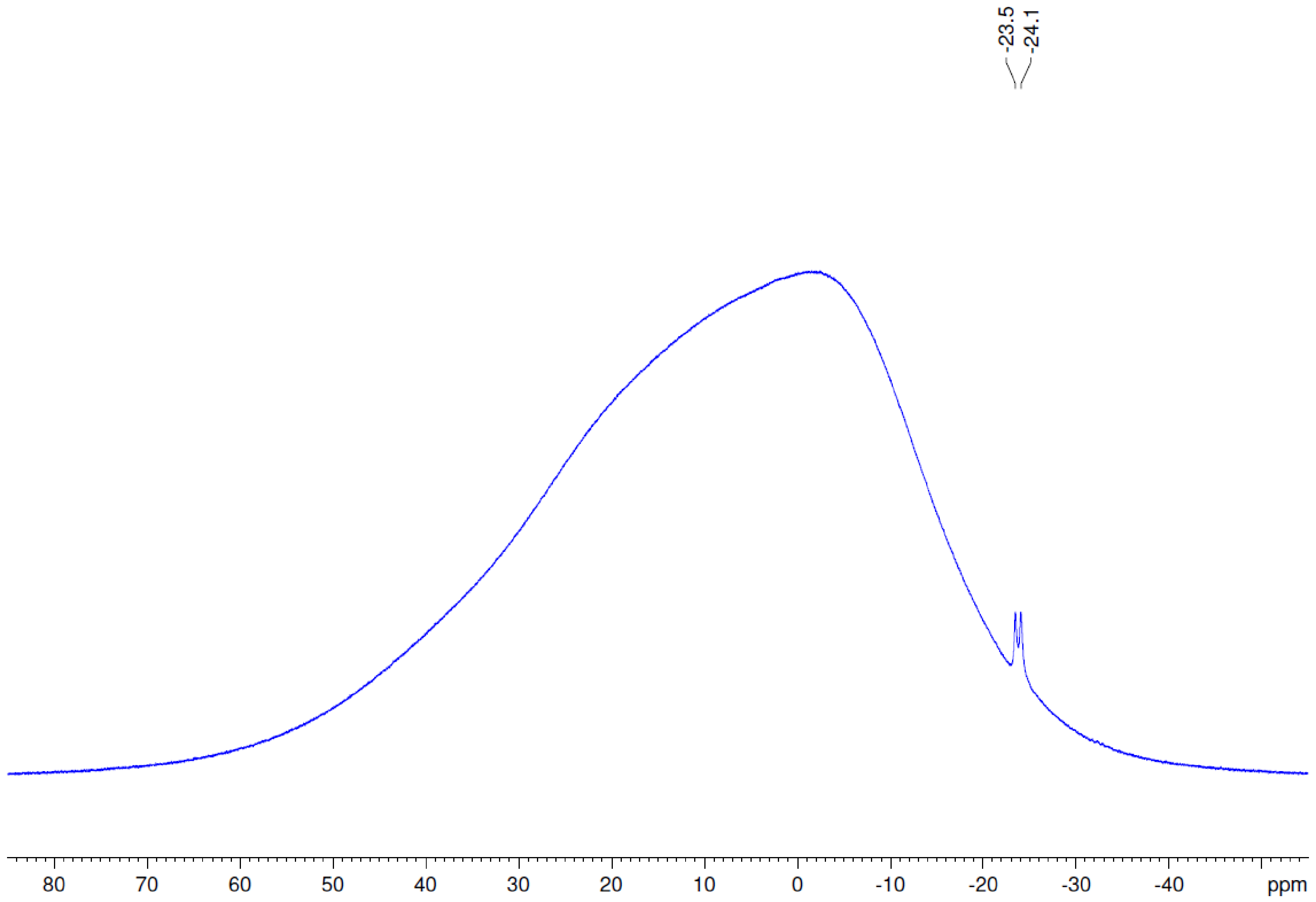

Figure S25. ^{11}B NMR spectrum of **4b^{CH2Cl}** in C_6D_6. The additional resonance at –32.3 ppm corresponds to **1b** (1%).

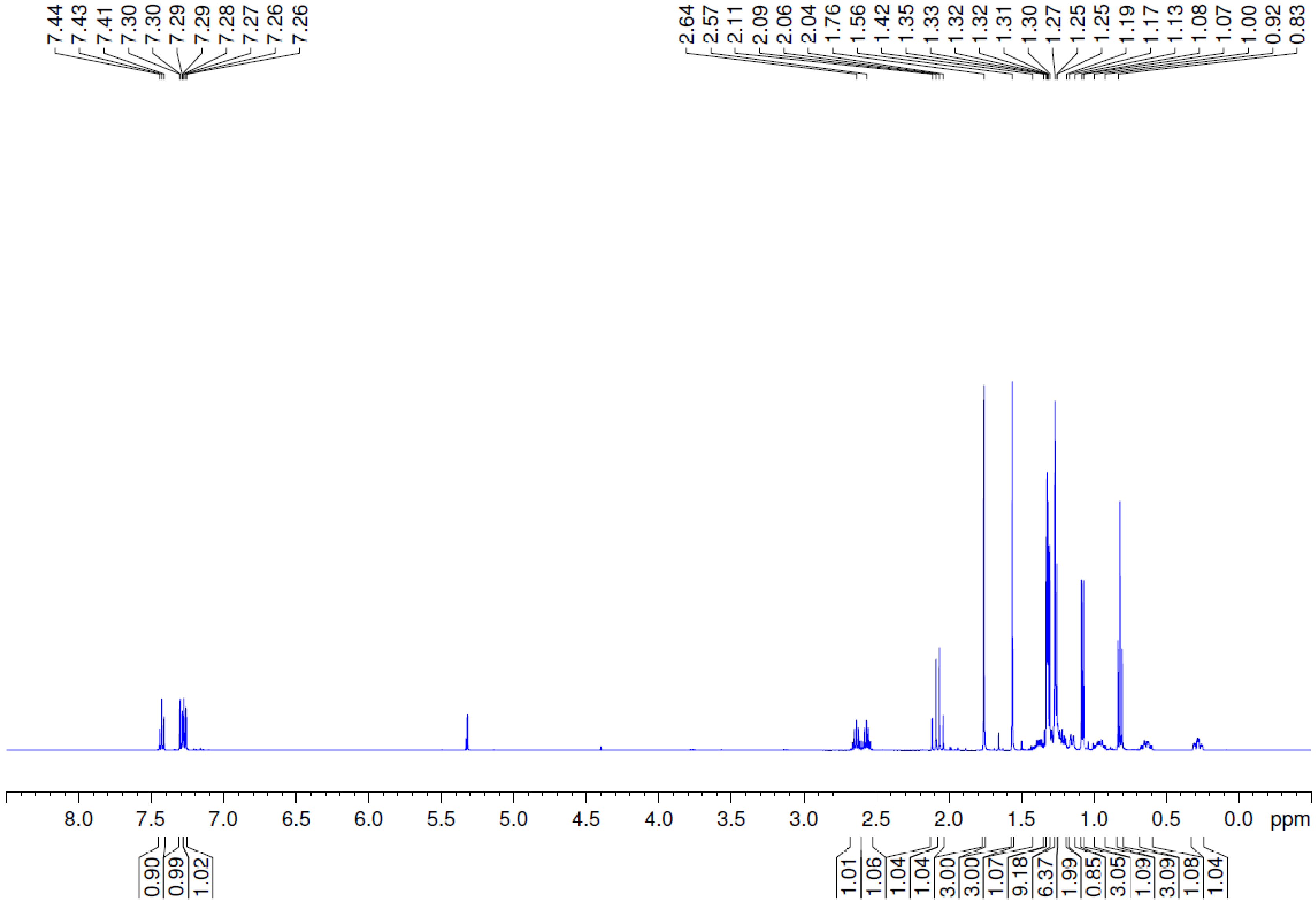

Figure S26. $^{1}H\{^{11}B\}$ NMR spectrum of **4a**$^{n\mathbf{Bu}}$ in CD_2Cl_2. The additional resonances correspond to **1a** (2%).

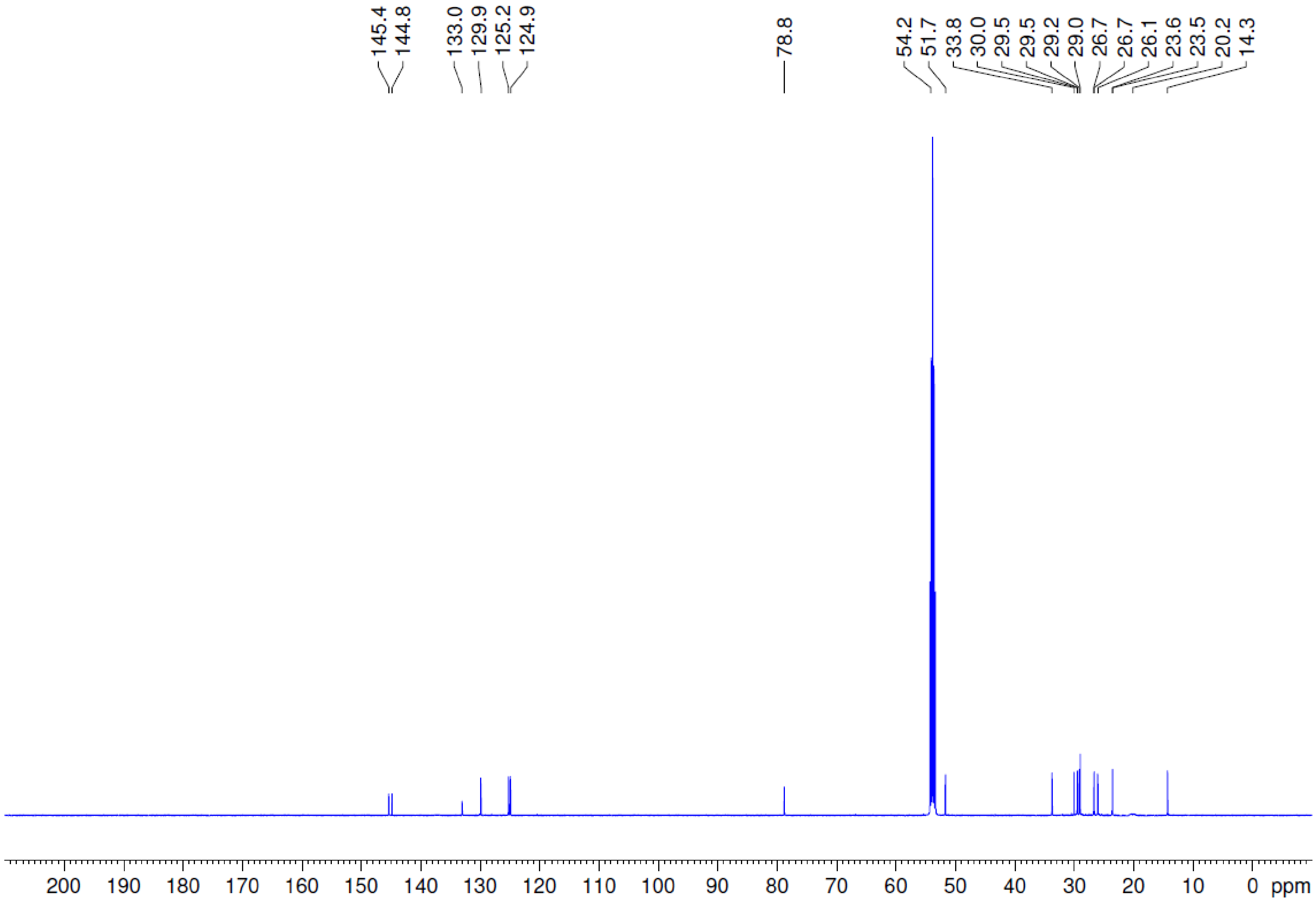

Figure S27. $^{13}C\{^{1}H\}$ NMR spectrum of **4a**^{nBu} in CD_2Cl_2.

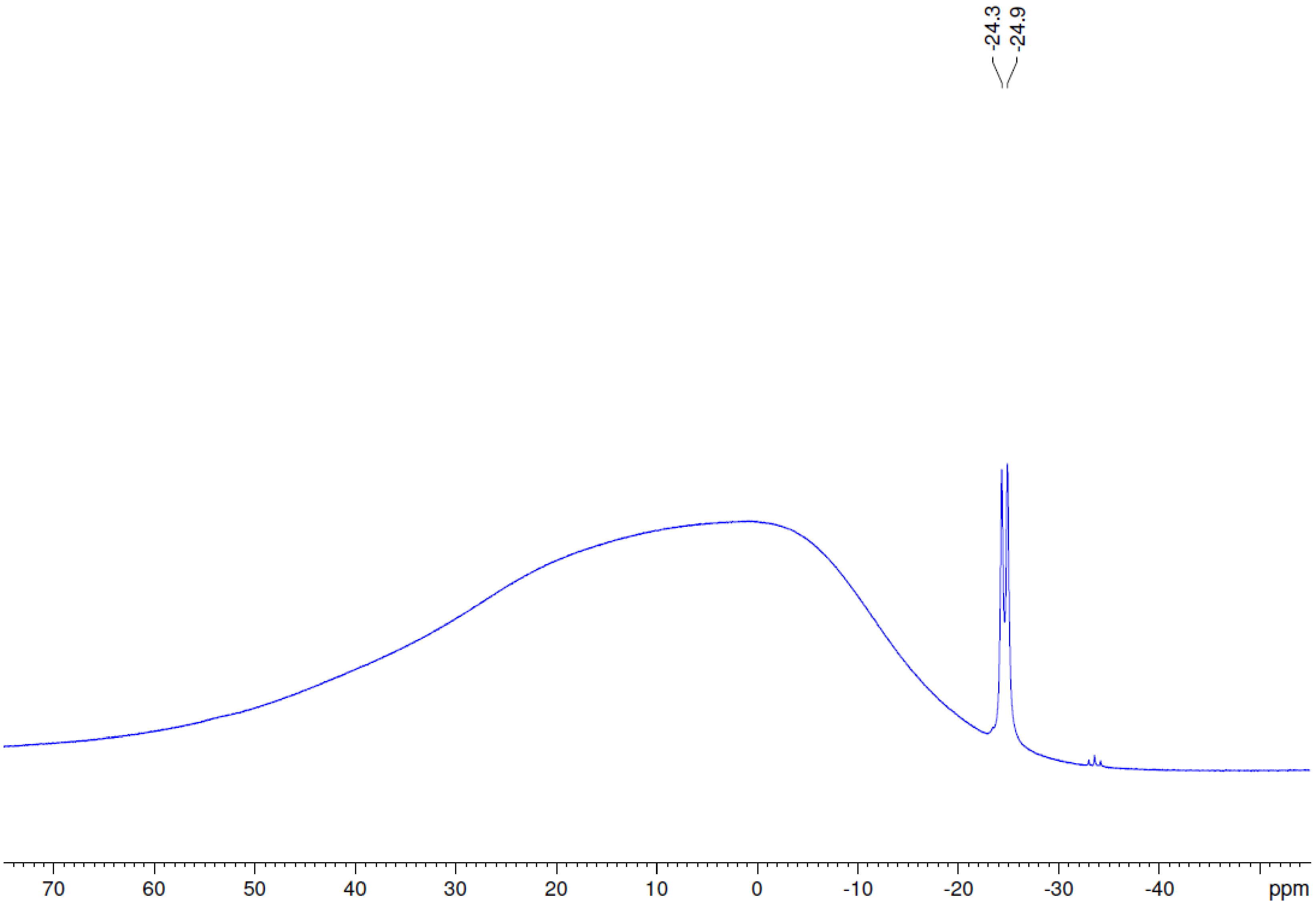

Figure S28. ^{11}B NMR spectrum of **4a*^{nBu}*** in CD_2Cl_2. The additional resonance at –33.6 ppm corresponds to **1a** (2%).

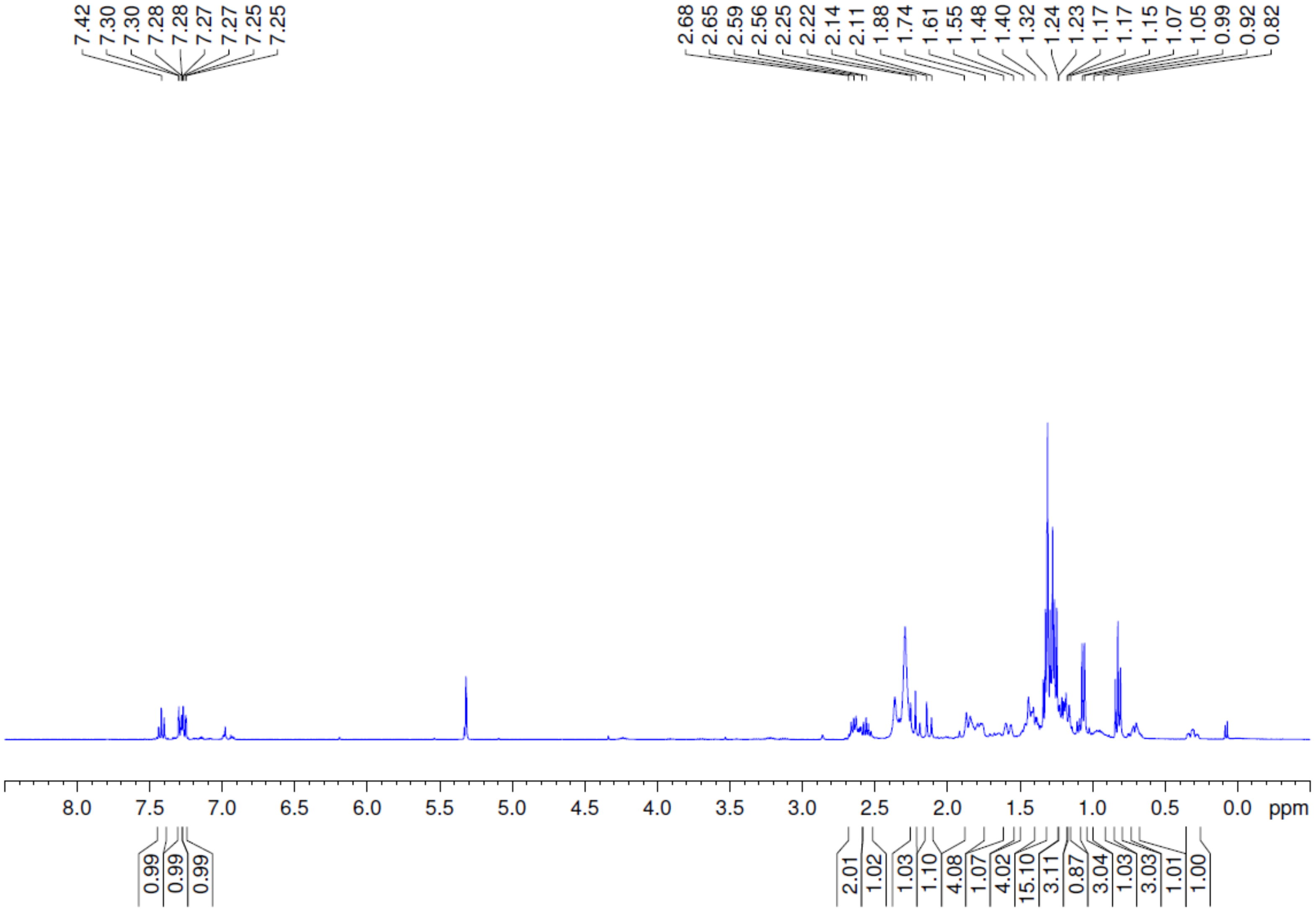

Figure S29. $^{1}H\{^{11}B\}$ NMR spectrum of **4b^{nBu}** in C_6D_6. The additional resonances at 2.29 and 2.36 ppm correspond to residual TMEDA. Other additional resonances correspond to **2b-TMEDA**.

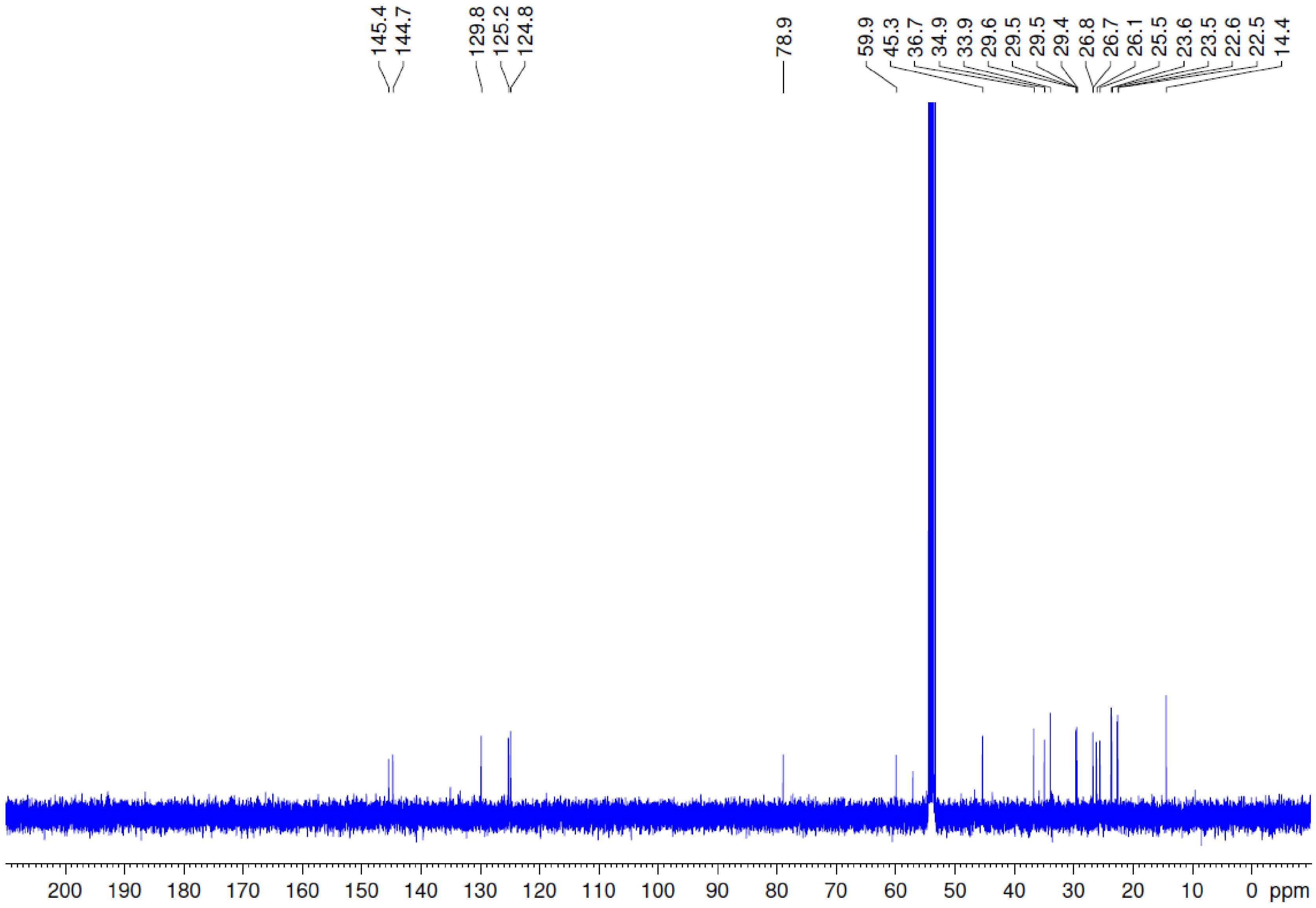

Figure S30. $^{13}C\{^{1}H\}$ NMR spectrum of **4b**^{nBu} in C_6D_6.

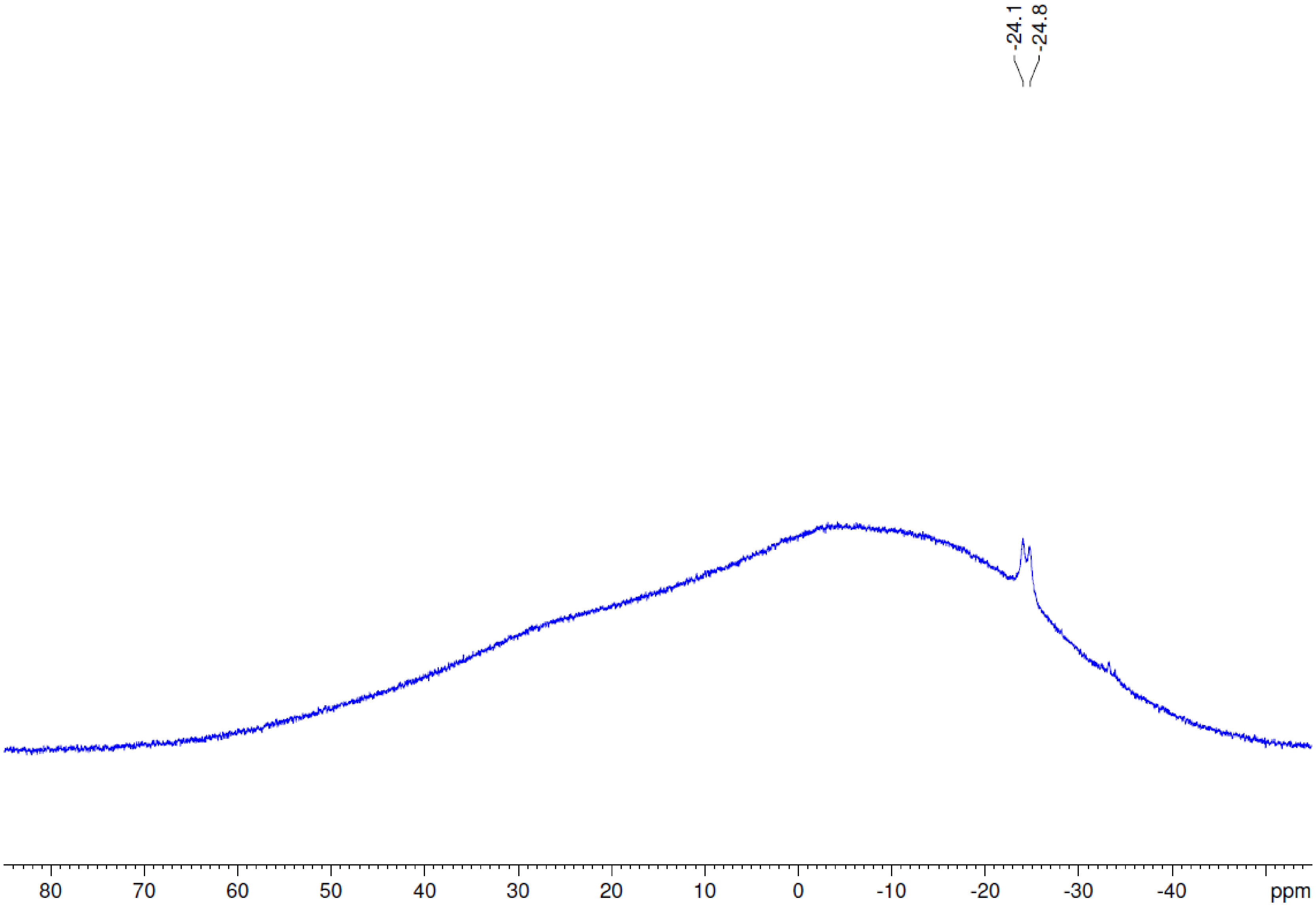

Figure S31. ^{11}B NMR spectrum of **4b**$^{\boldsymbol{n}\mathbf{Bu}}$ in CD_2Cl_2. The additional resonance at –33.2 ppm corresponds to **1b**.

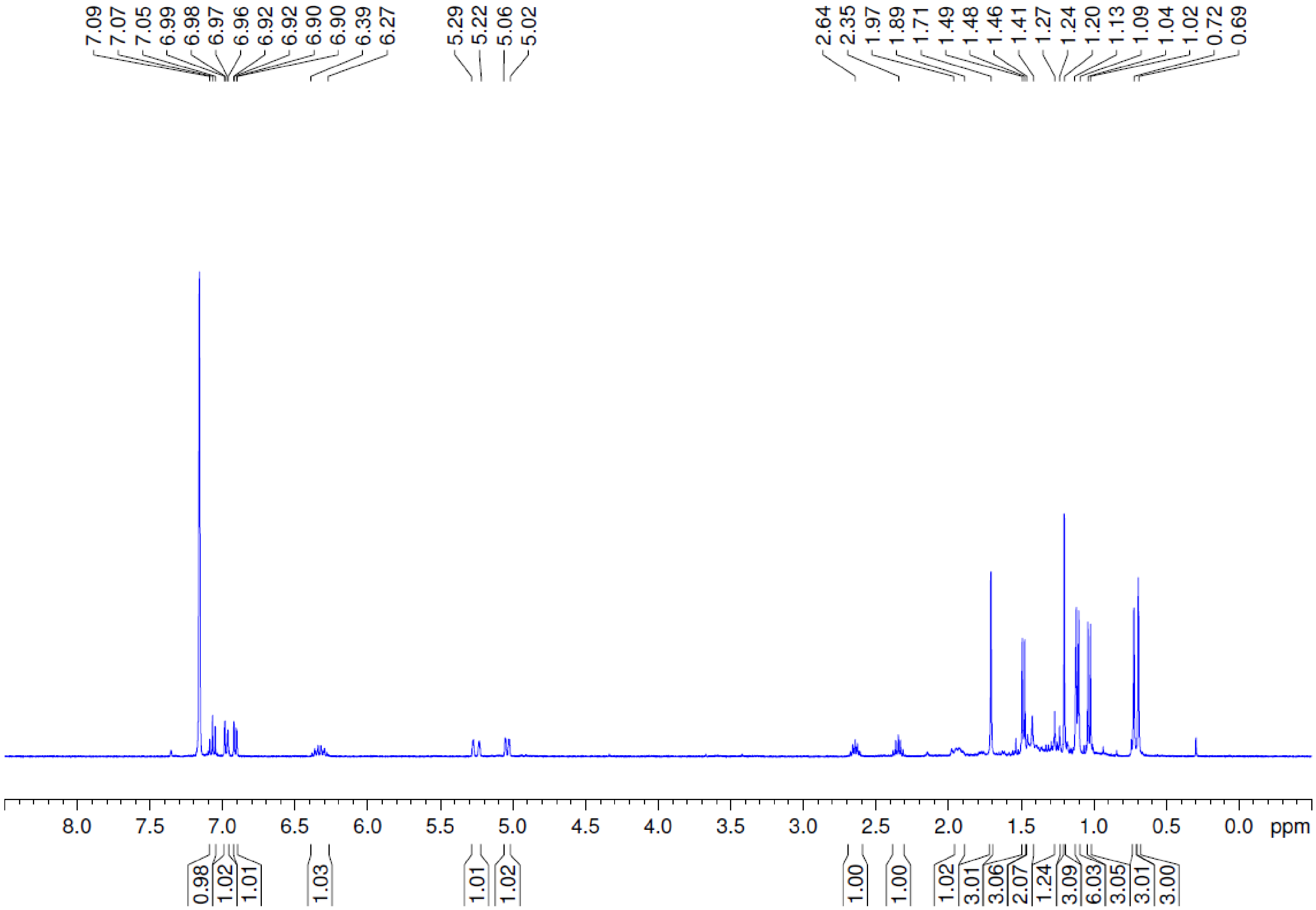

Figure S32. $^{1}H\{^{11}B\}$ NMR spectrum of **4a**$^{\mathbf{C3H5}}$ in C_6D_6. The additional resonances correspond to **1a** (1%).

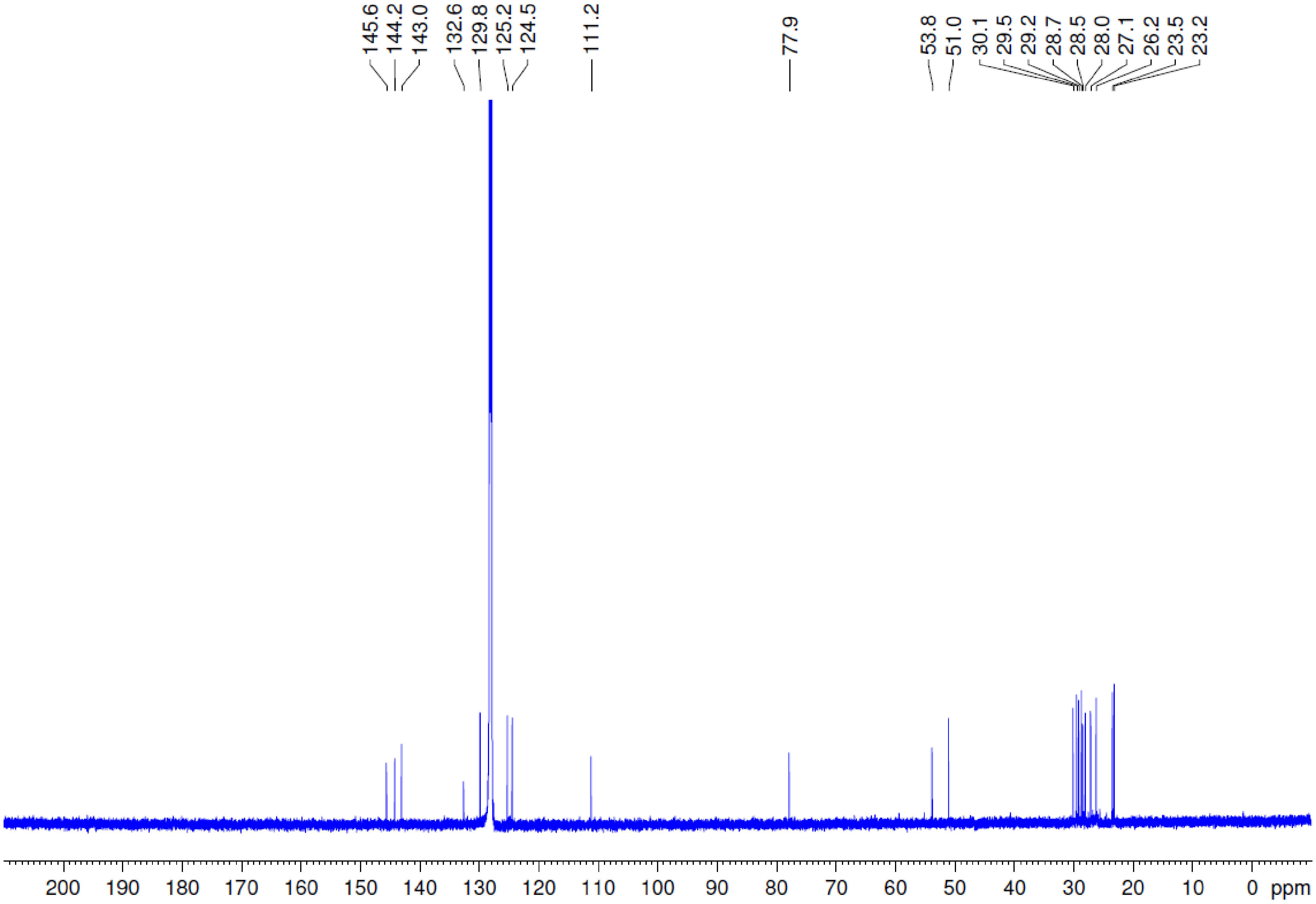

Figure S33. $^{13}C\{^1H\}$ NMR spectrum of **4a**$^{\mathbf{C3H5}}$ in C_6D_6.

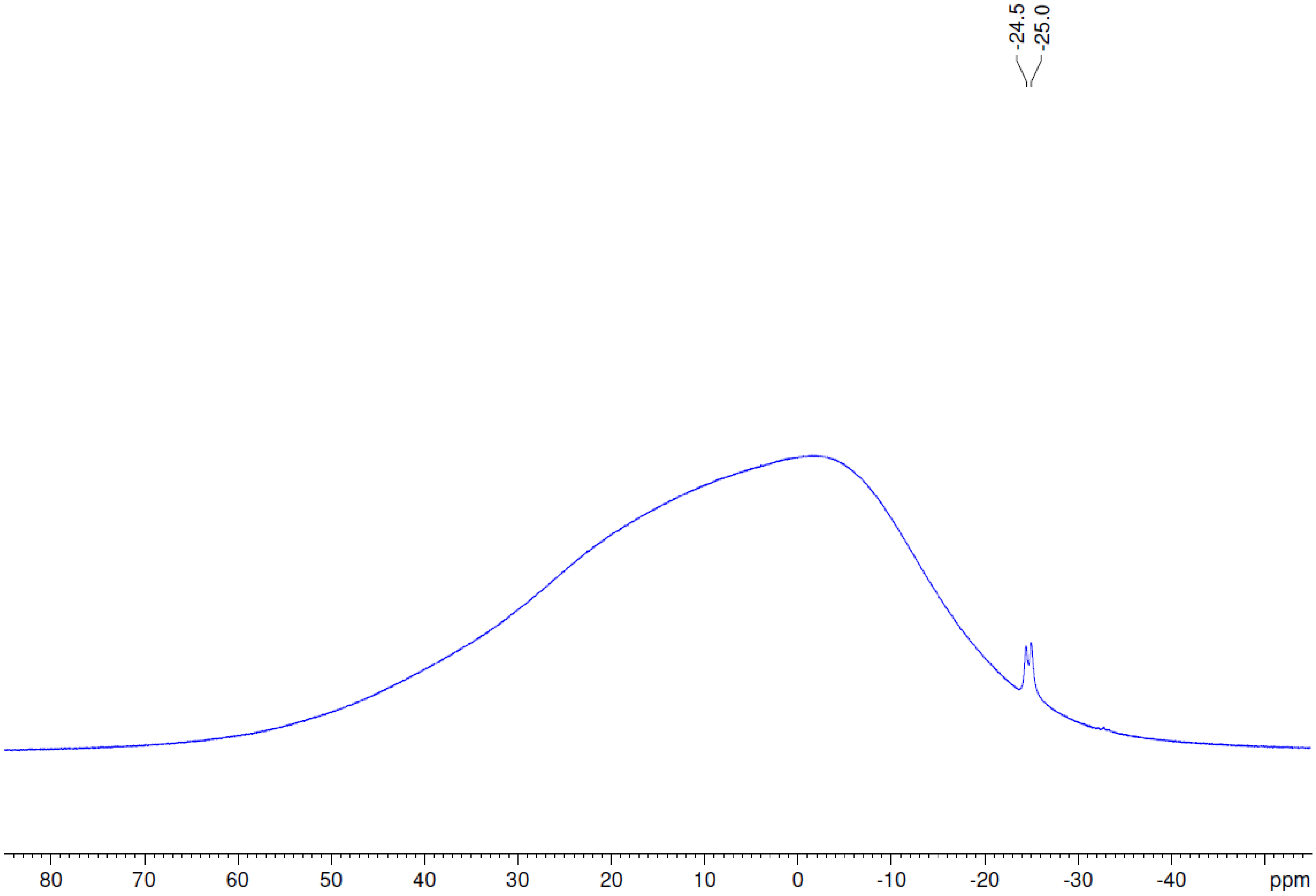

Figure S34. ^{11}B NMR spectrum of **4a**$^{\mathbf{C3H5}}$ in C_6D_6. The additional resonance at –32.8 ppm correspond to **1a** (1%).

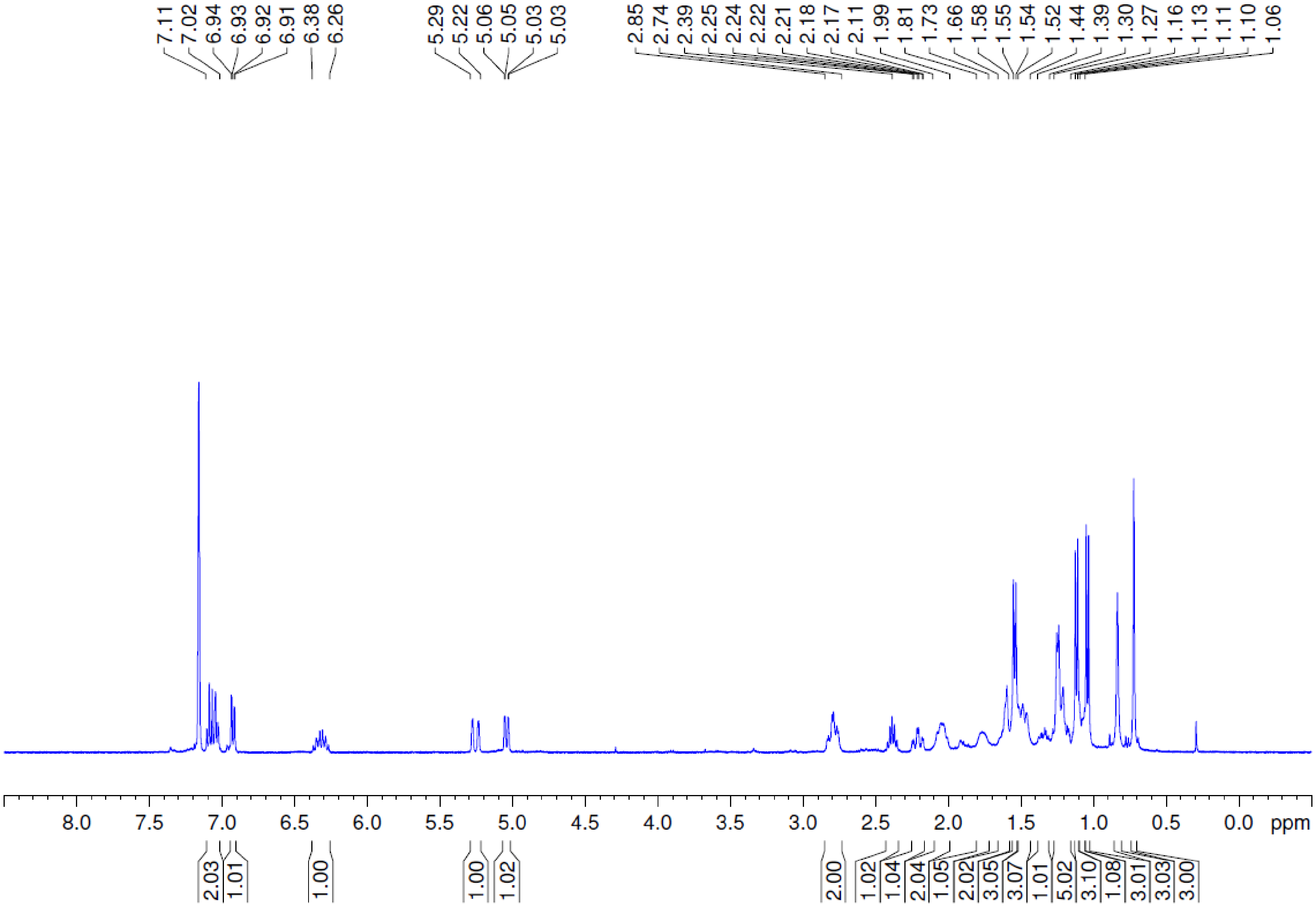

Figure S35. $^{1}H\{^{11}B\}$ NMR spectrum of **4b**$^{\mathbf{C3H5}}$ in C_6D_6. The additional resonances correspond to **1b** (3%).

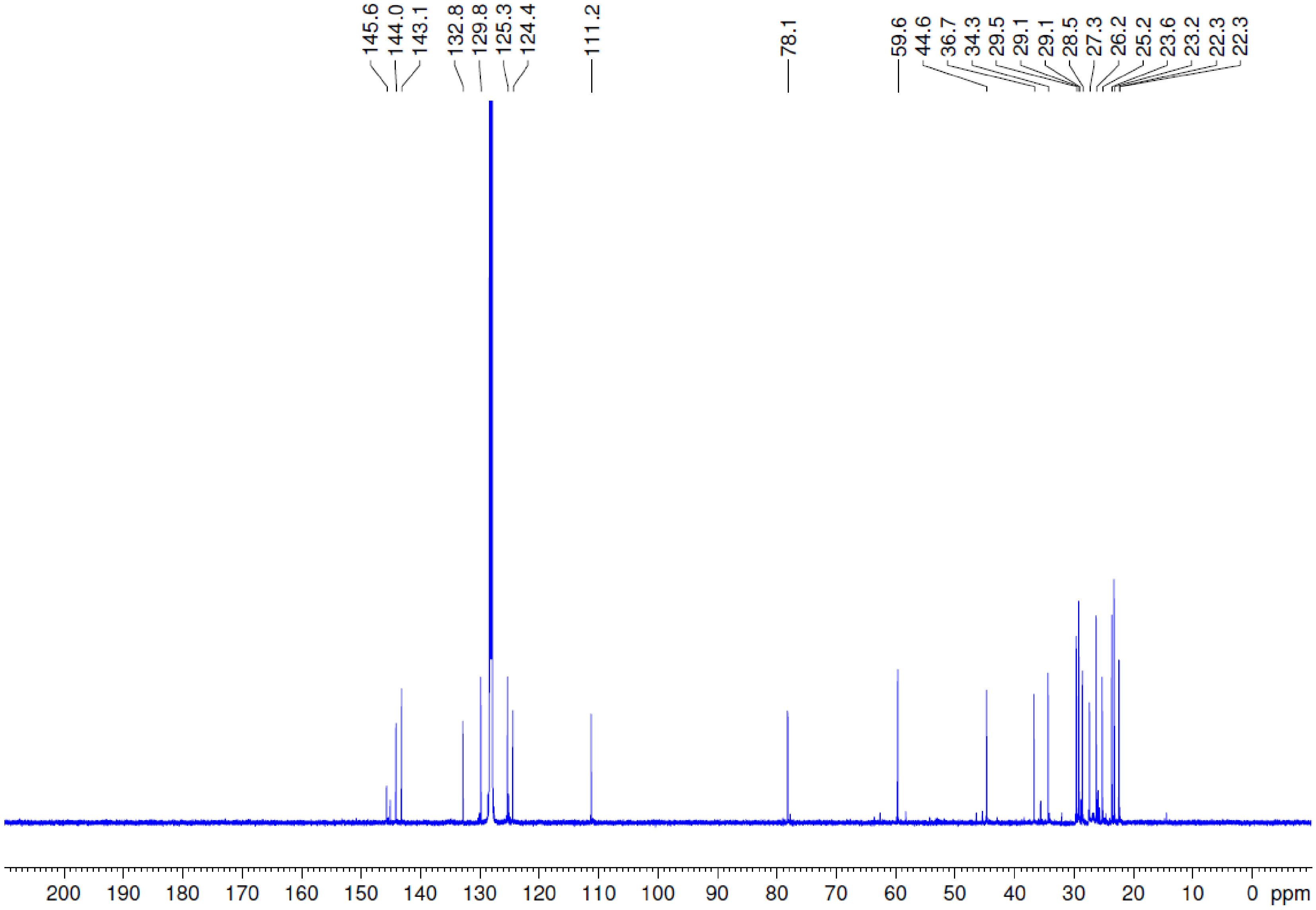

Figure S36. $^{13}C\{^{1}H\}$ NMR spectrum of **4b**$^{\mathbf{C3H5}}$ in C_6D_6. The additional resonances correspond to **1b** (3%).

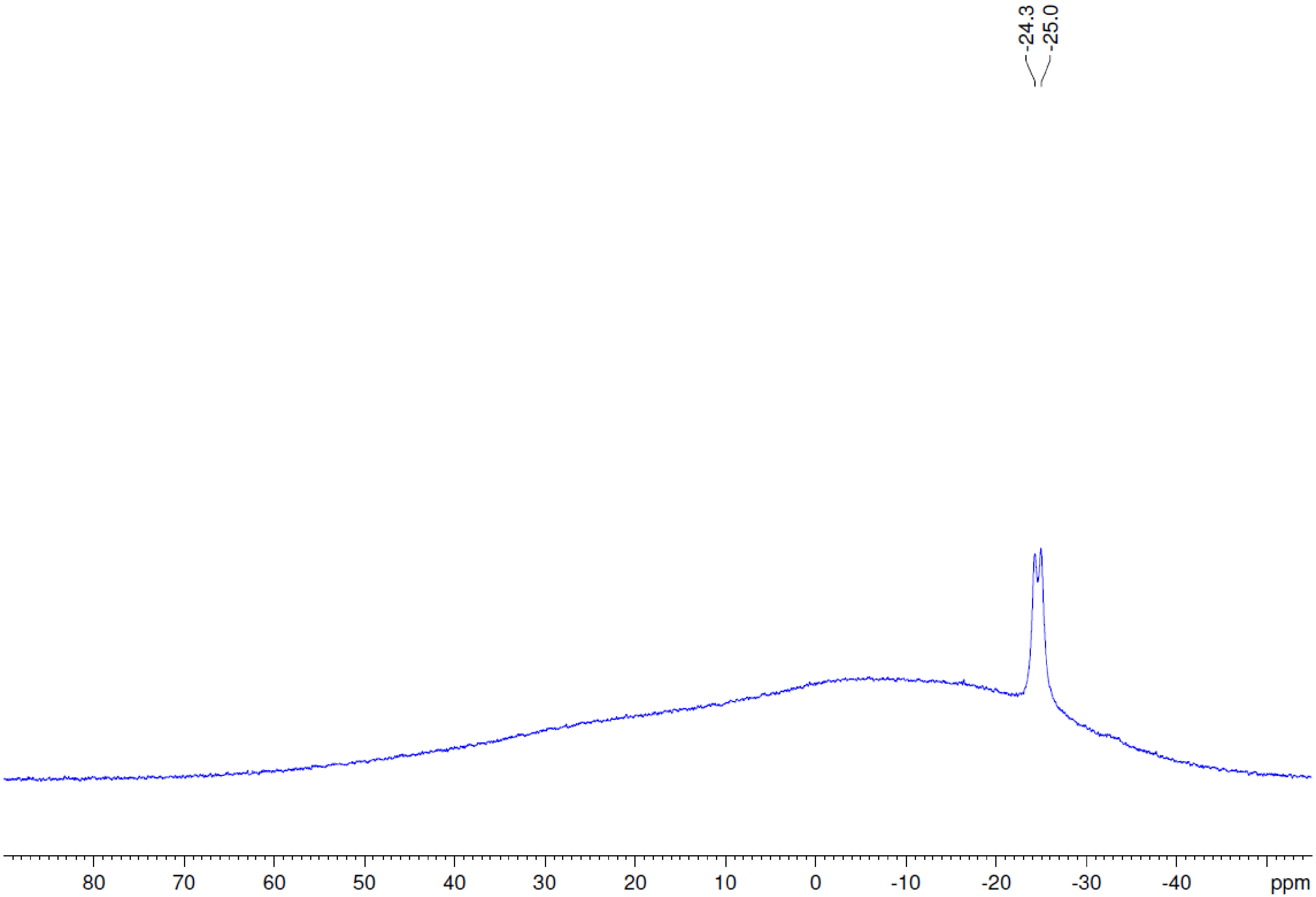

Figure S37. ^{11}B NMR spectrum of **4b^{C3H5}** in C_6D_6. The additional resonance at –32.8 ppm corresponds to **1b** (3%).

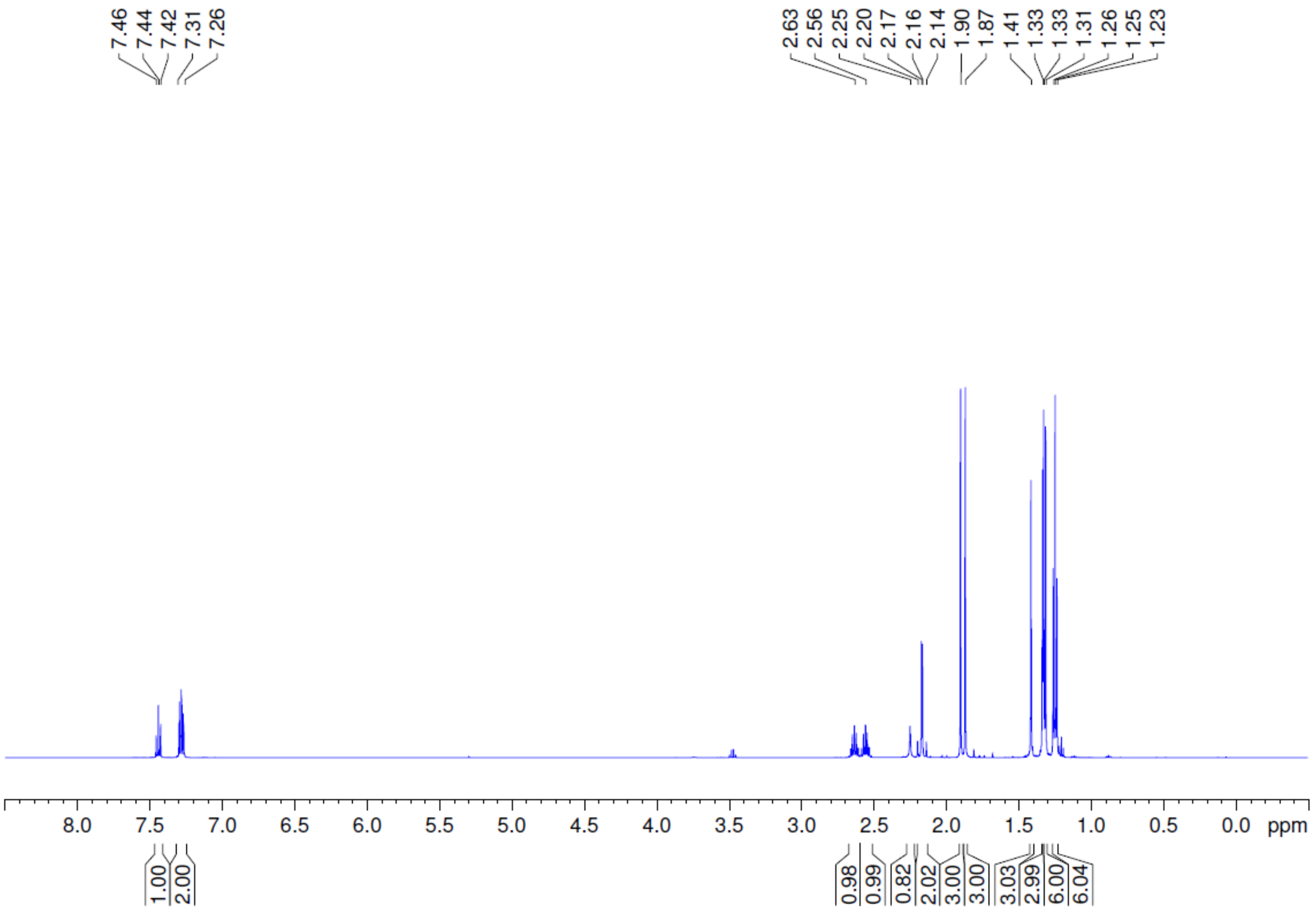

Figure S38. $^{1}H\{^{11}B\}$ NMR spectrum of $\mathbf{4a^{Br}}$ in $CDCl_3$. The resonances at 1.23 and 3.48 ppm correspond to residual diethyl ether. Other additional resonances correspond to **1a** and an unknown impurity.

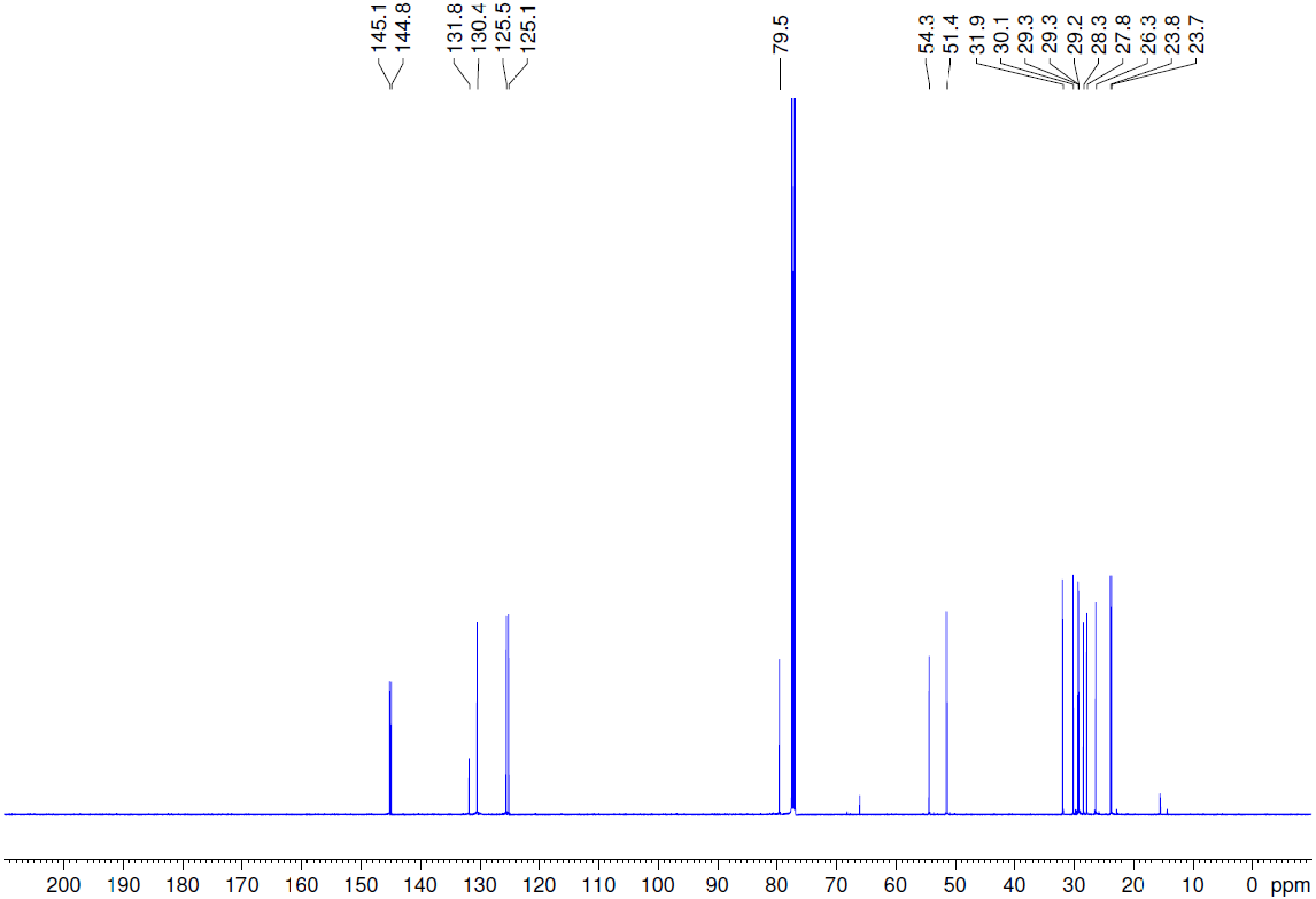

Figure S39. $^{13}C\{^{1}H\}$ NMR spectrum of **4a^{Br}** in $CDCl_3$. Additional resonances correspond to **1a** and an unknown impurity.

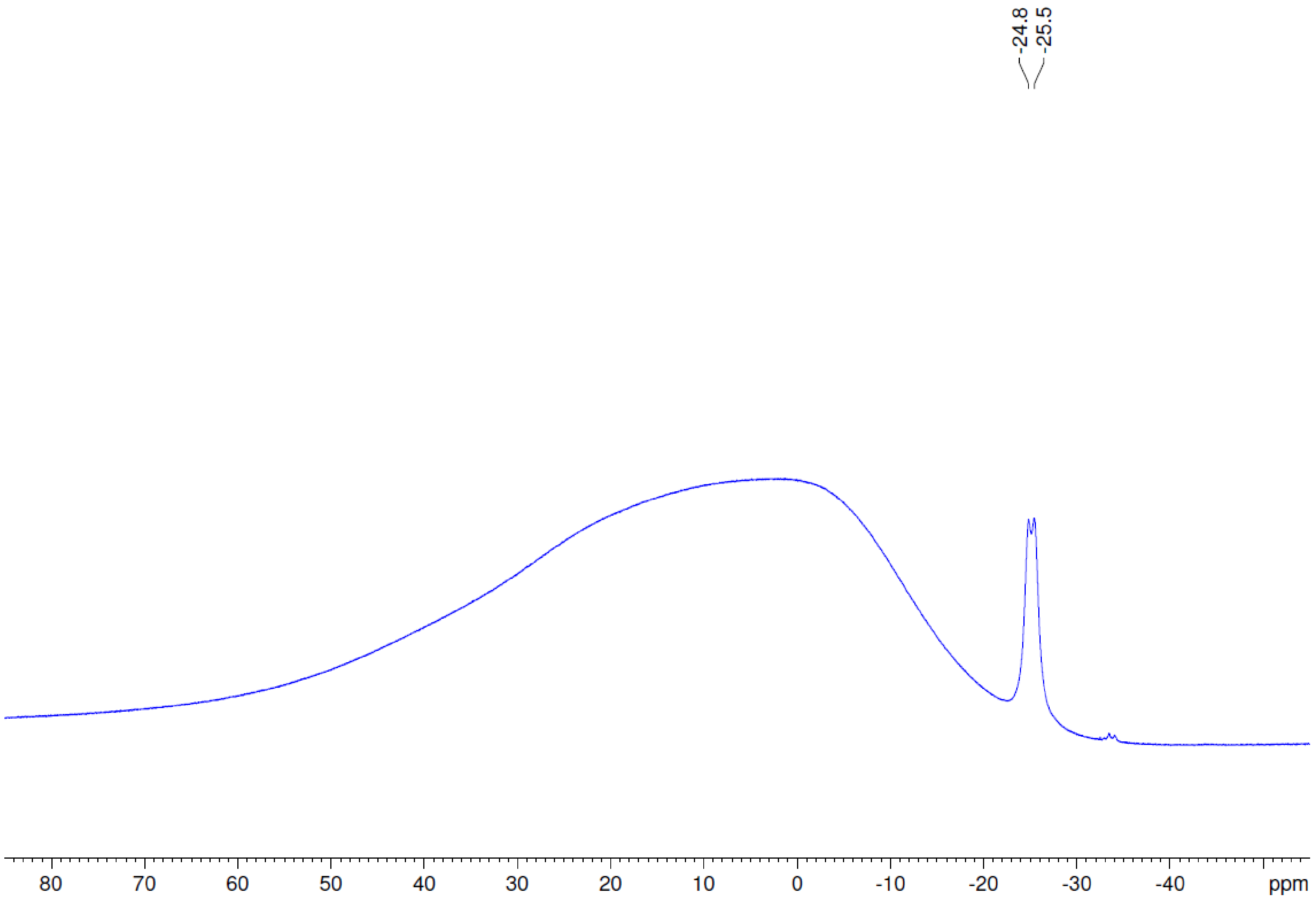

Figure S40. ^{11}B NMR spectrum of **4a$^{\mathbf{Br}}$** in $CDCl_3$. The additional resonances at –33.5 and –33.8 ppm belong to **1a** and an unknown impurity.

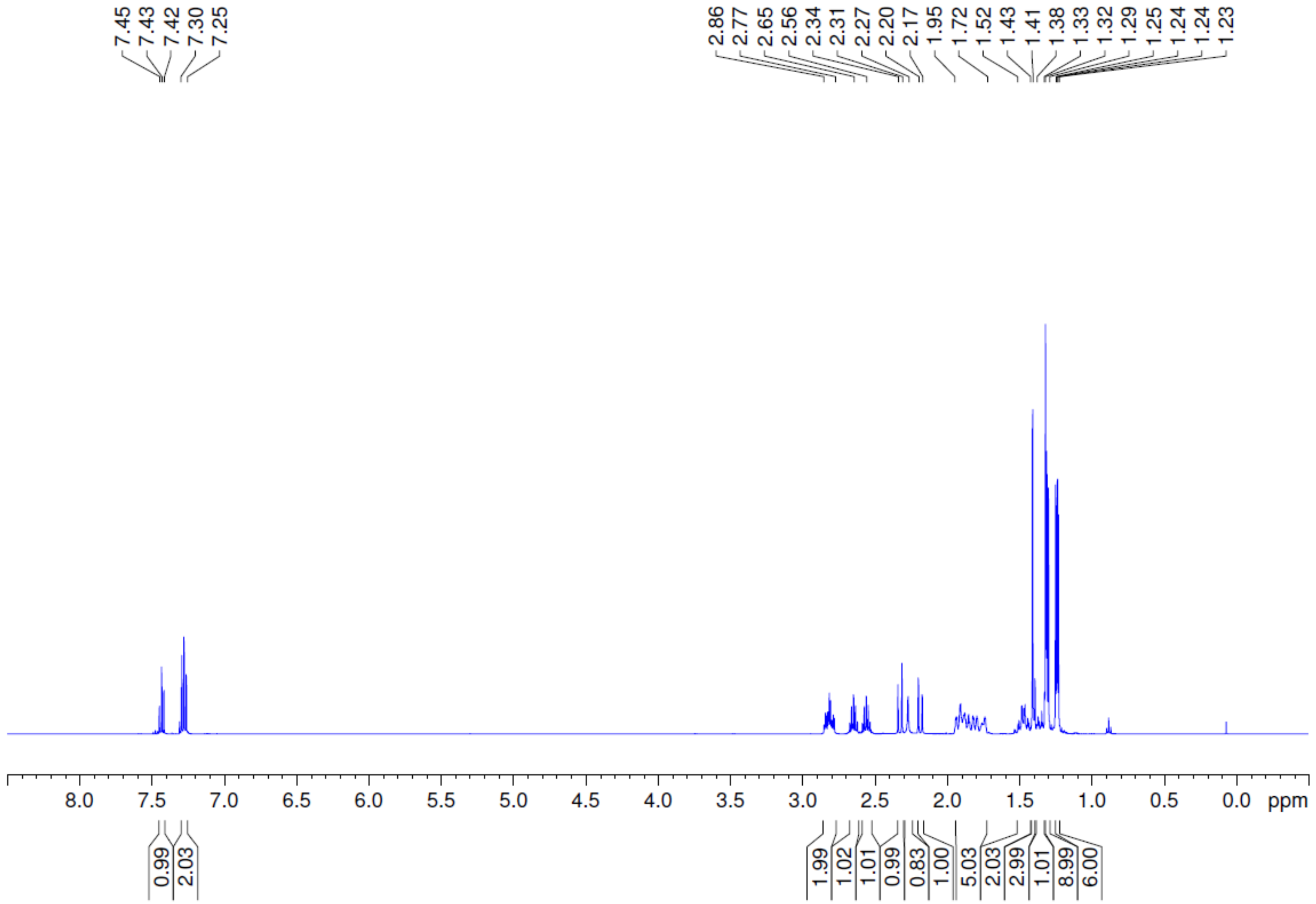

Figure S41. $^{1}H\{^{11}B\}$ NMR spectrum of **4b^{Br}** in $CDCl_3$. The additional resonance at 0.88 ppm corresponds to residual hexane. Other additional resonances correspond to **1b** and an unknown impurity.

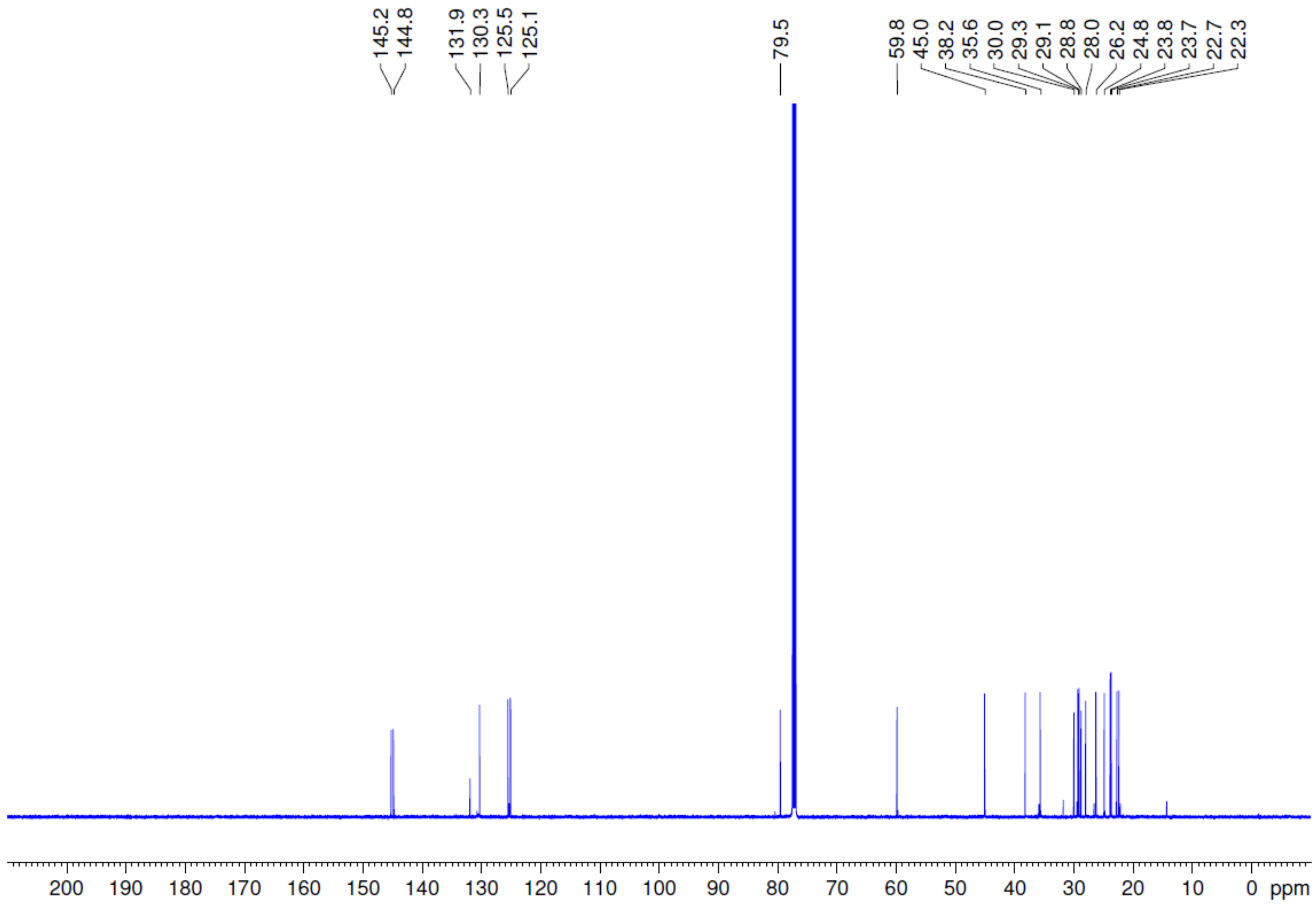

Figure S42. $^{13}C\{^{1}H\}$ NMR spectrum of **4b^{Br}** in $CDCl_3$. The additional resonances at 14.24, 22.80 and 31.74 ppm correspond to residual hexane. Other additional resonances correspond to **1b** and an unknown impurity.

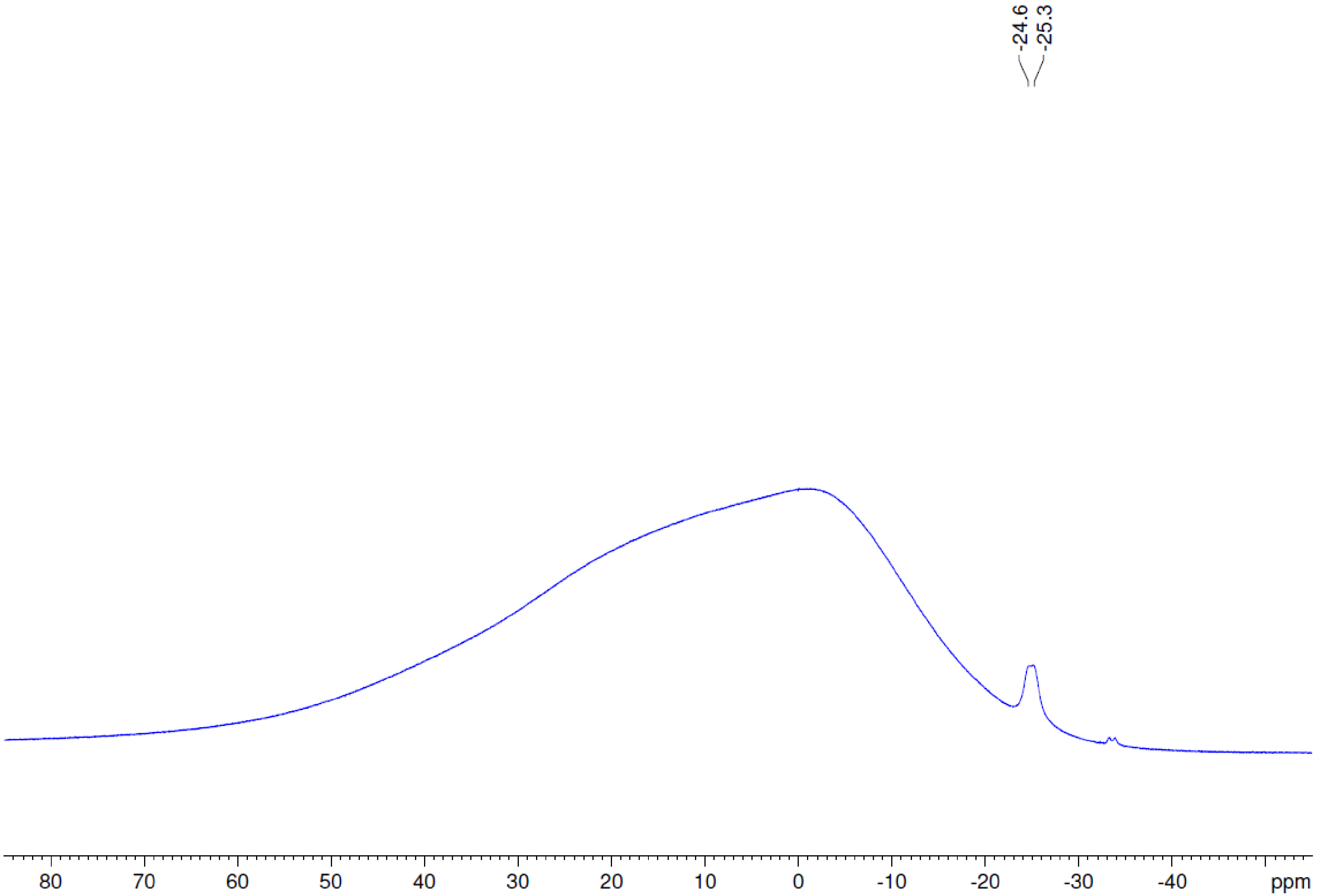

Figure S43. ^{11}B NMR spectrum of **4b^{Br}** in $CDCl_3$. The additional resonances at –33.5 and –33.8 ppm belong to **1b** and an unknown impurity.

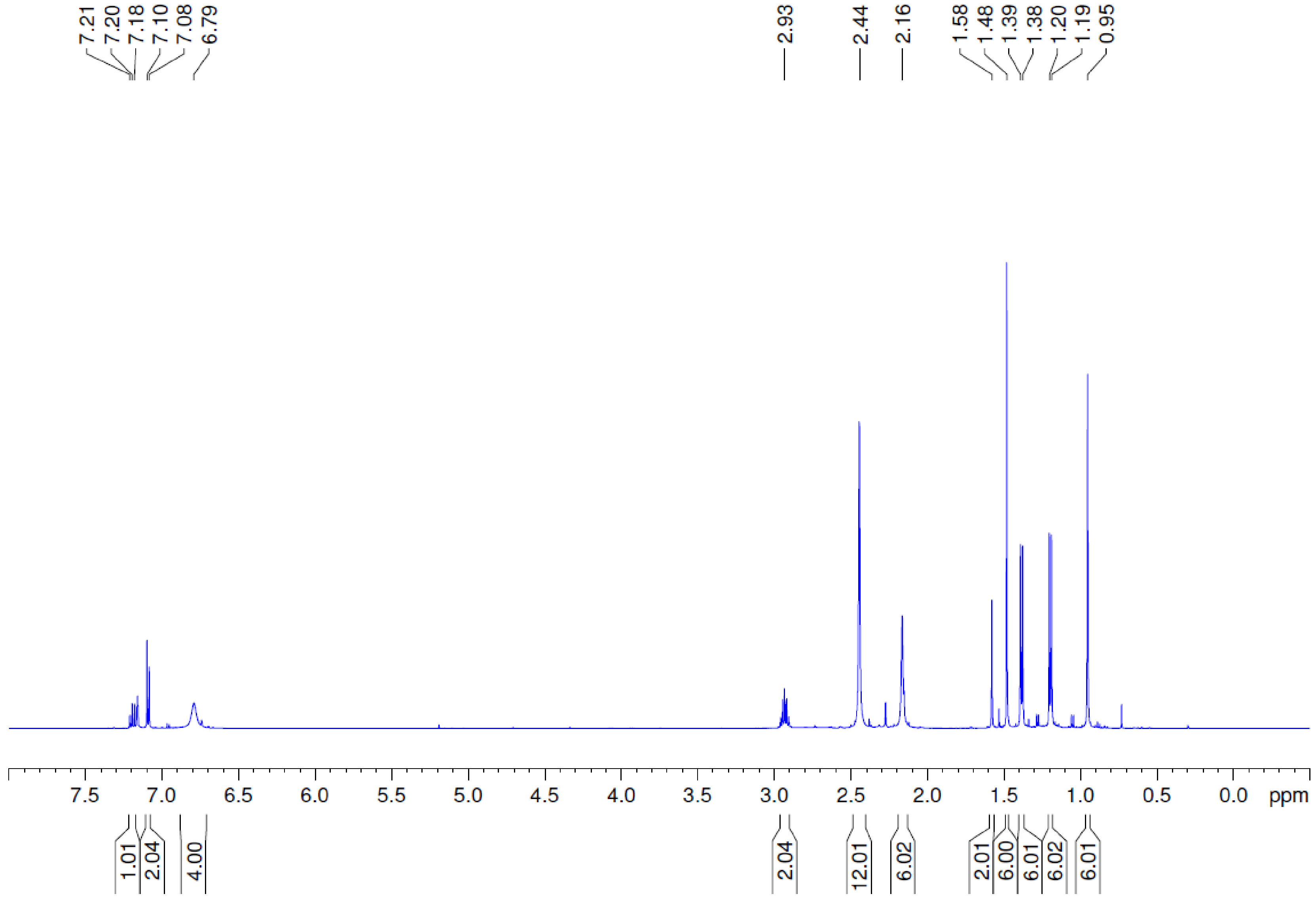

Figure S44. $^{1}H\{^{11}B\}$ NMR spectrum of **5a^{Mes}** in C_6D_6. The additional resonances correspond to **1a** (4%).

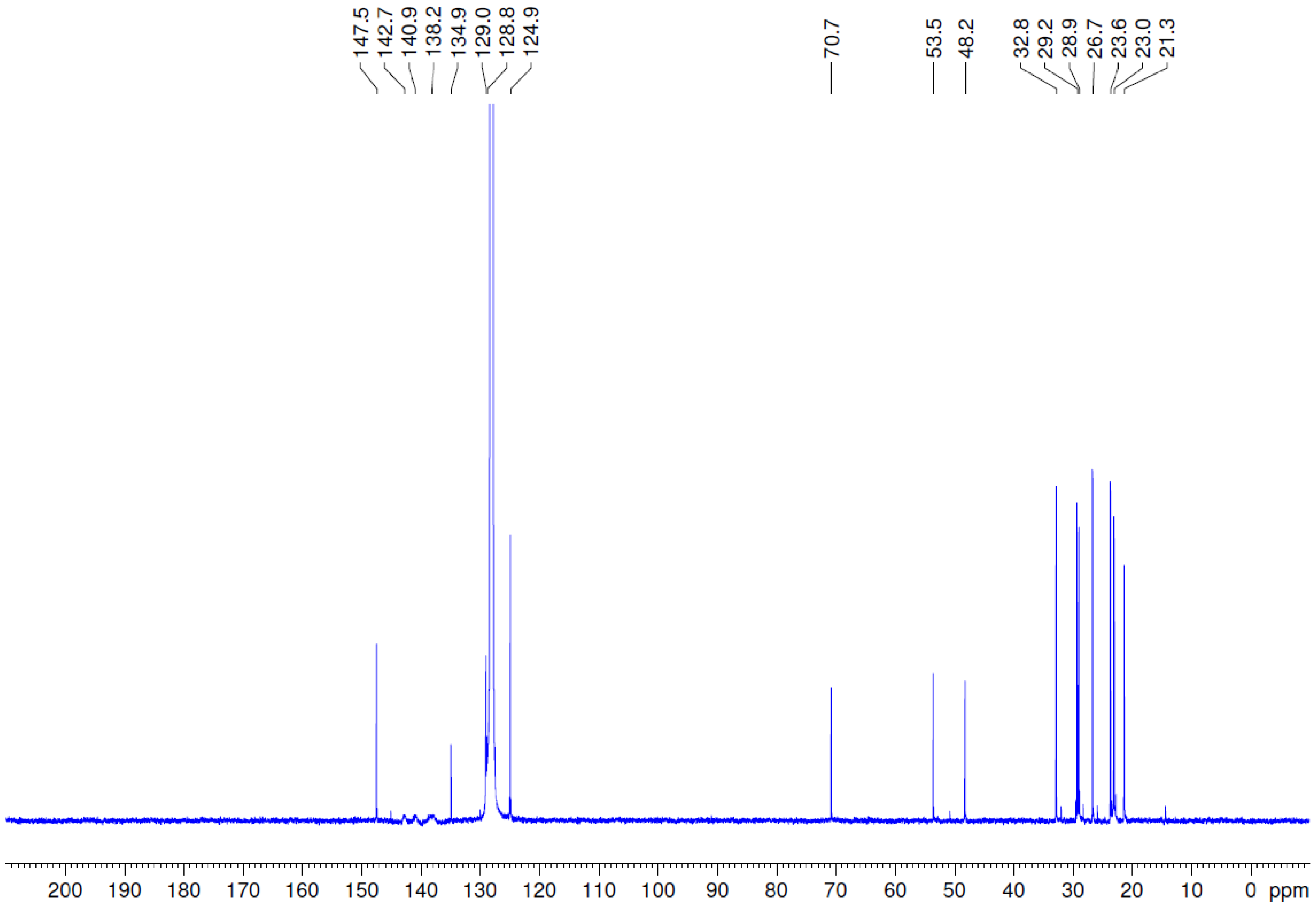

Figure S45. $^{13}C\{^{1}H\}$ NMR spectrum of **5a$^{\mathbf{Mes}}$** in C_6D_6.

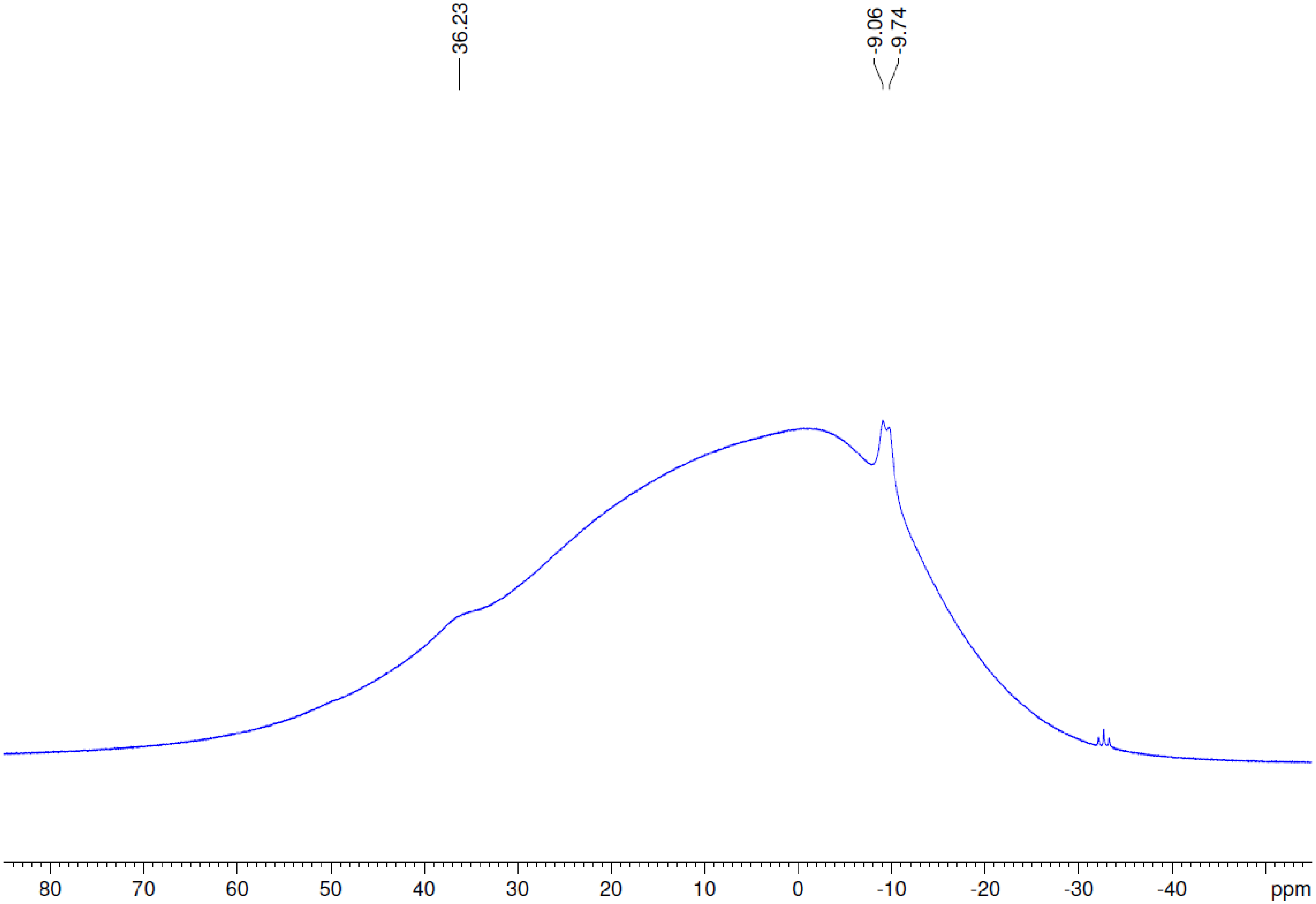

Figure S46. ^{11}B NMR spectrum of **5a^{Mes}** in C_6D_6. The additional resonance at –32.7 ppm correspond to **1a** (4%).

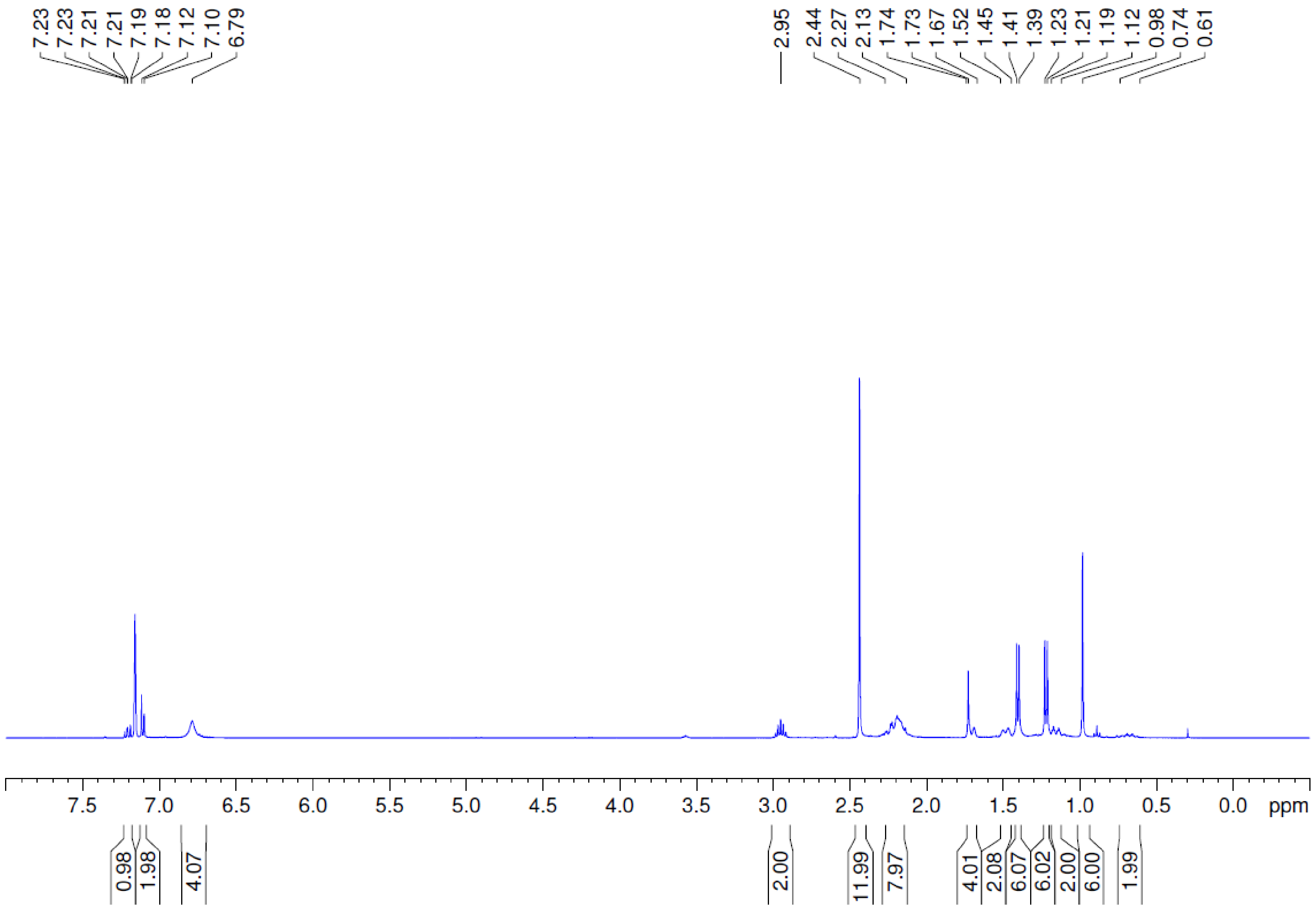

Figure S47. ^{1}H NMR spectrum of **5b$^{\mathbf{Mes}}$** in C_6D_6. The additional resonance at 0.89 ppm corresponds to residual hexane.

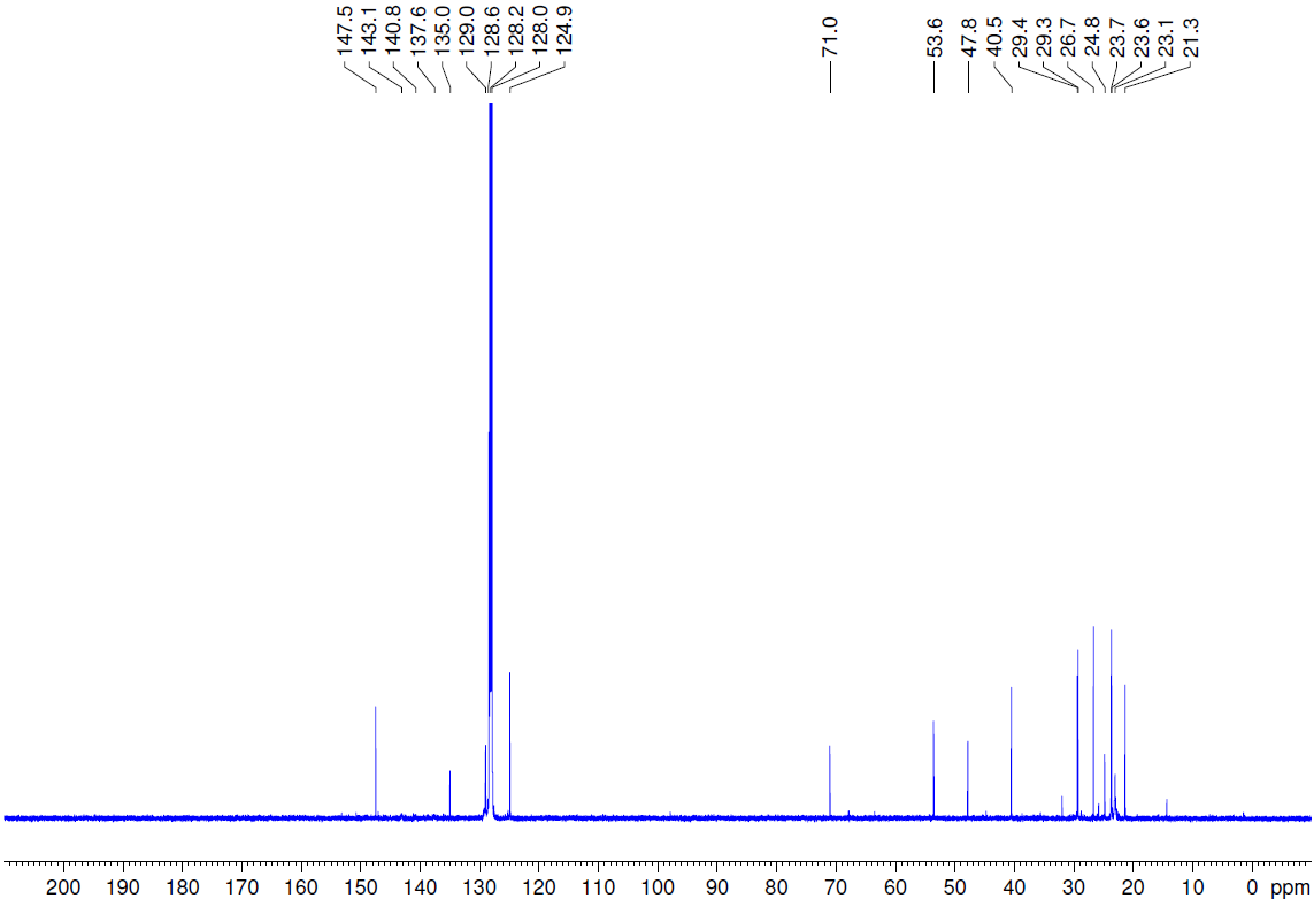

Figure S48. $^{13}C\{^{1}H\}$ NMR spectrum of $\mathbf{5b^{Mes}}$ in C_6D_6. The additional resonances at 14.4, 23.06 and 32.0 correspond to residual hexane.

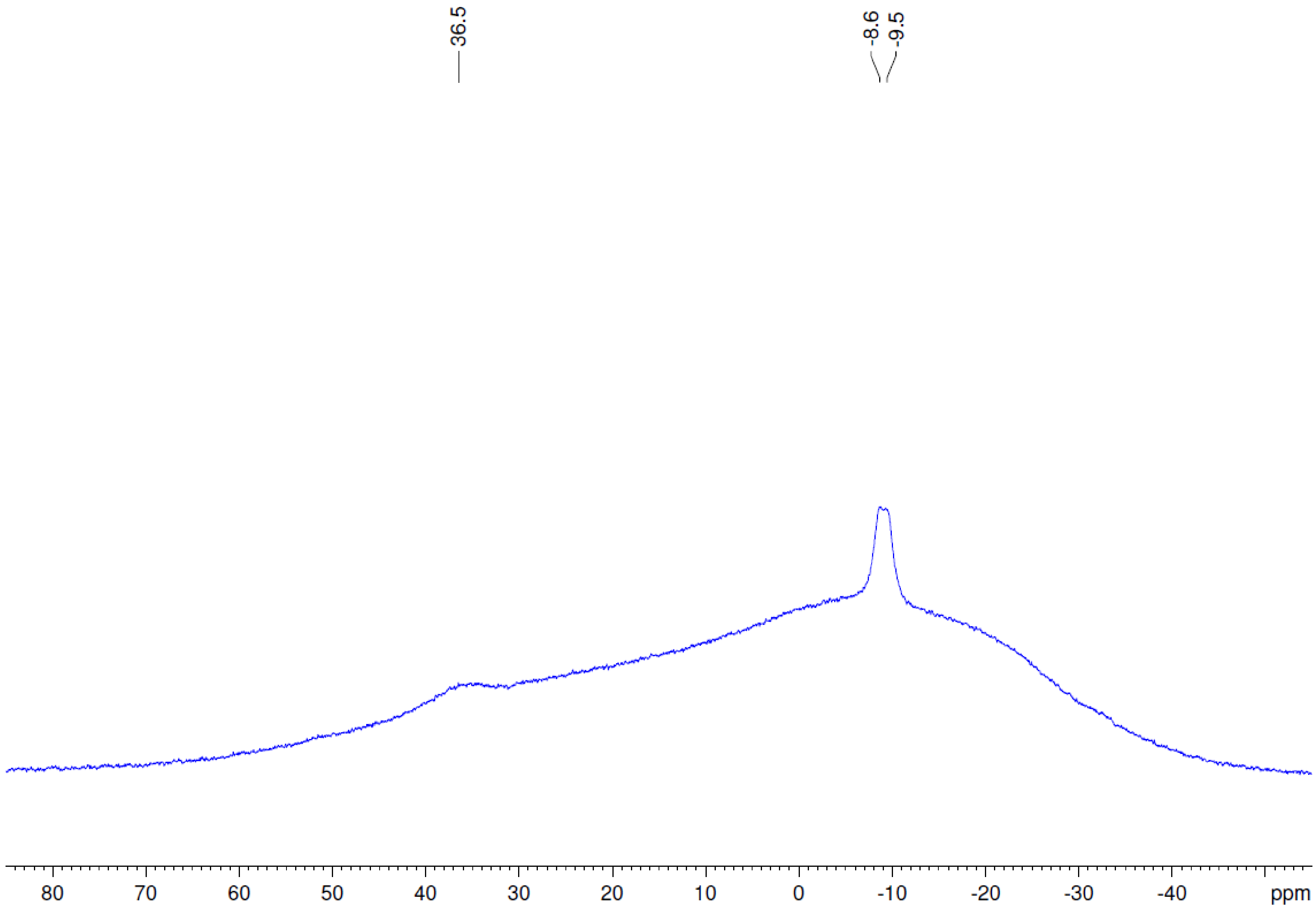

Figure S49. ^{11}B NMR spectrum of **5b^{Mes}** in C_6D_6.

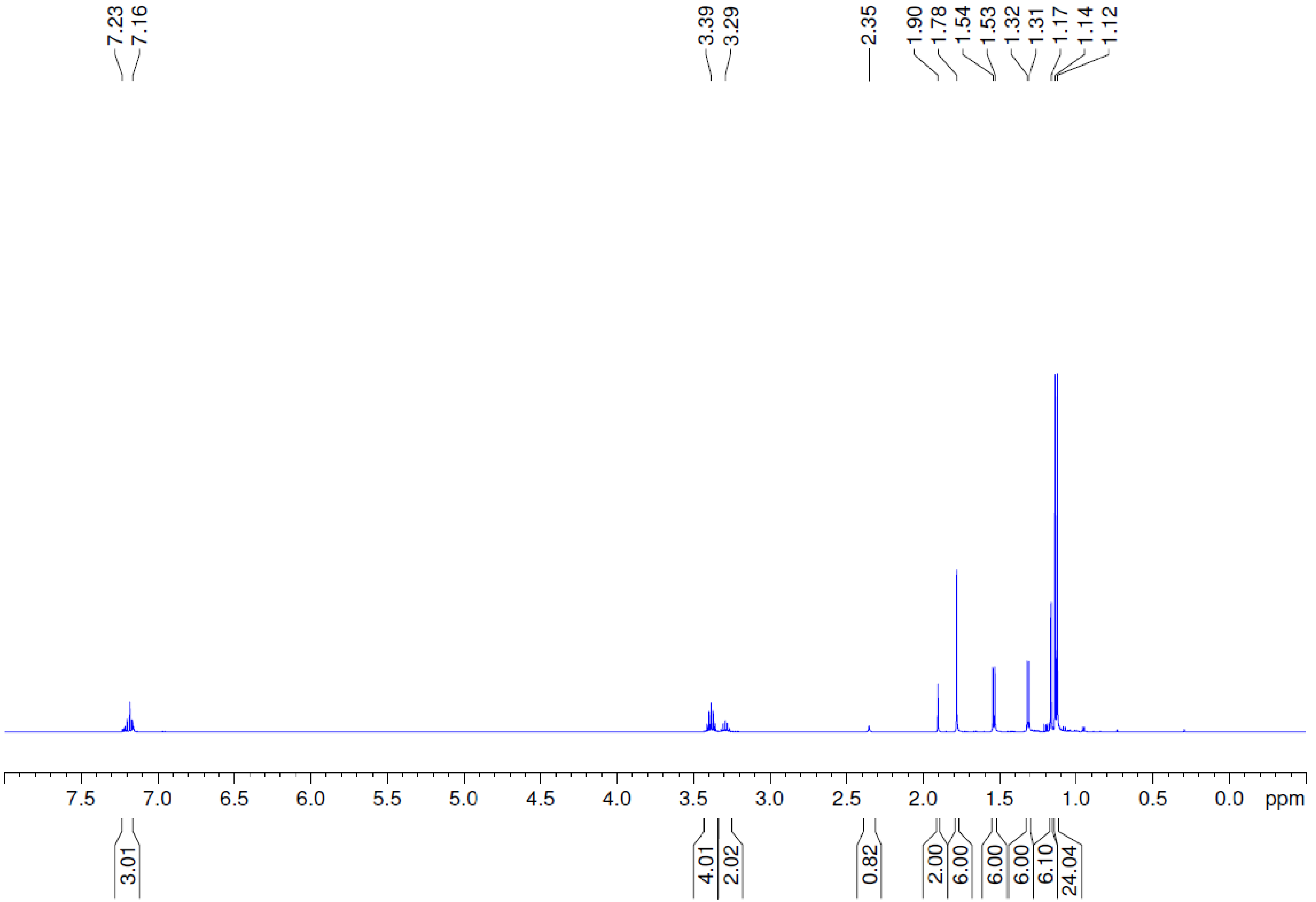

Figure S50. $^{1}H\{^{11}B\}$ NMR spectrum of **5a$^{\mathbf{N}\boldsymbol{i}\mathbf{Pr2}}$** in C_6D_6.

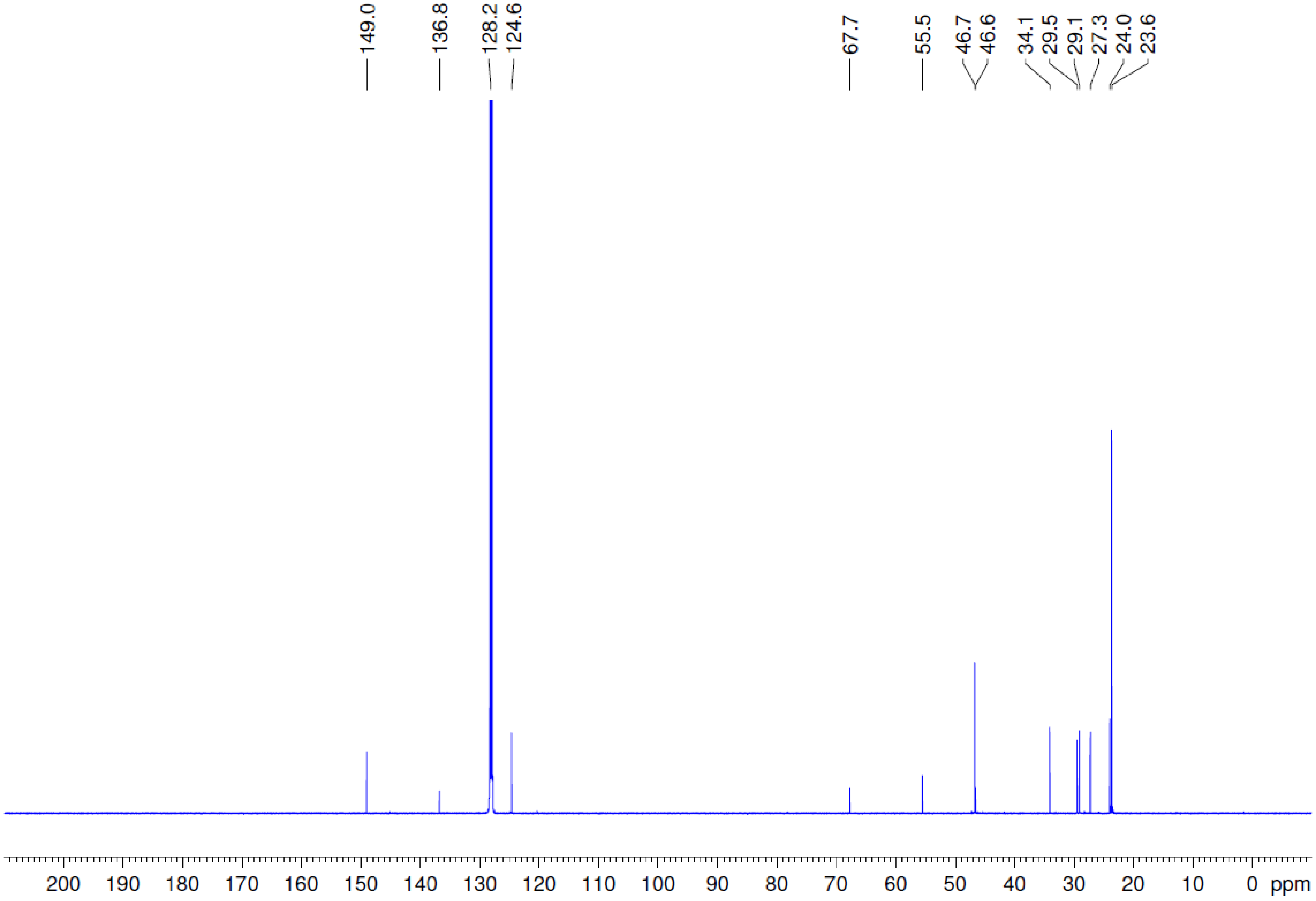

Figure S51. $^{13}C\{^{1}H\}$ NMR spectrum of **5a$^{\mathbf{N}\boldsymbol{i}\mathbf{Pr2}}$** in C_6D_6.

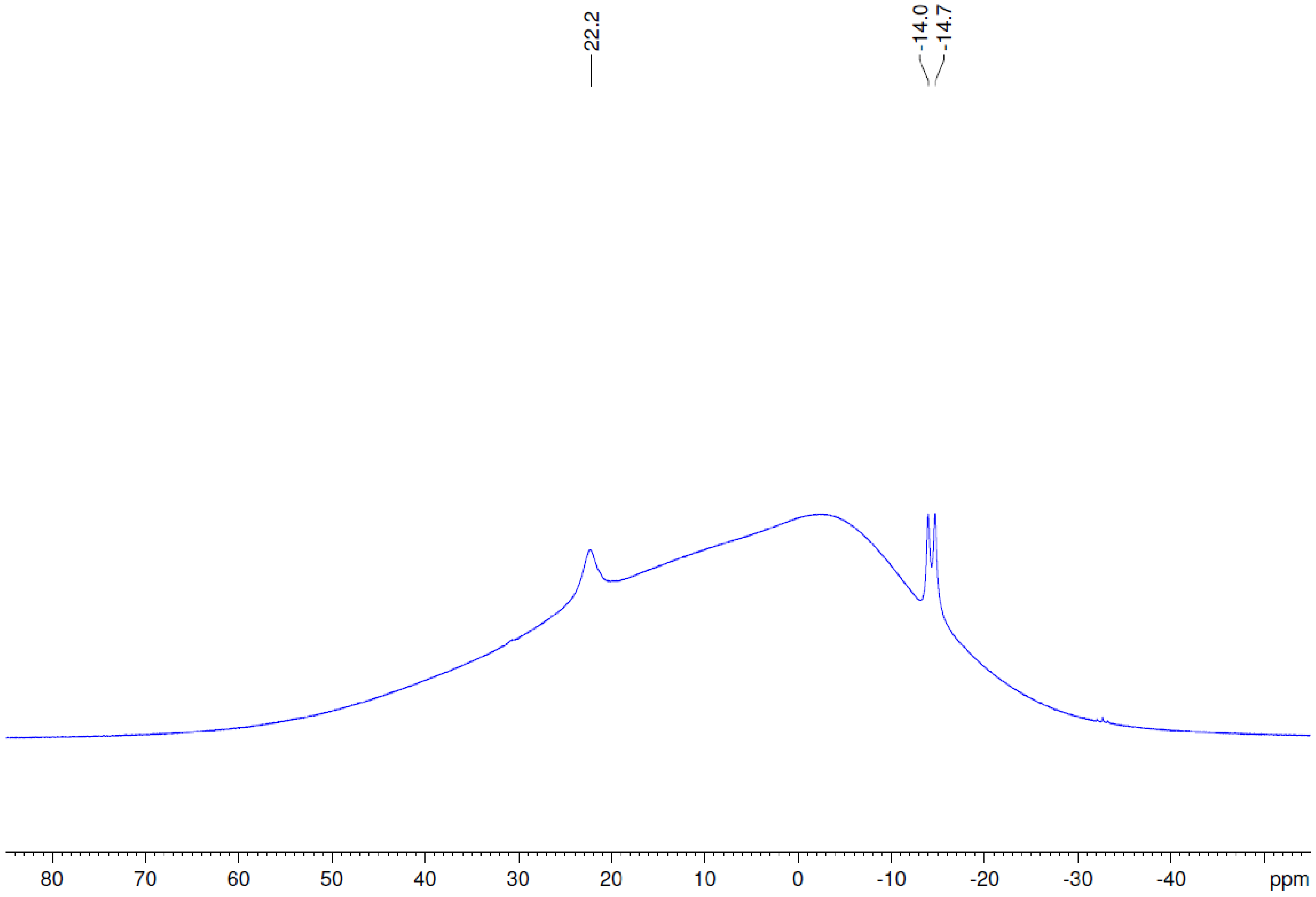

Figure S52. ^{11}B NMR spectrum of **5a^{NiPr2}** in C_6D_6.

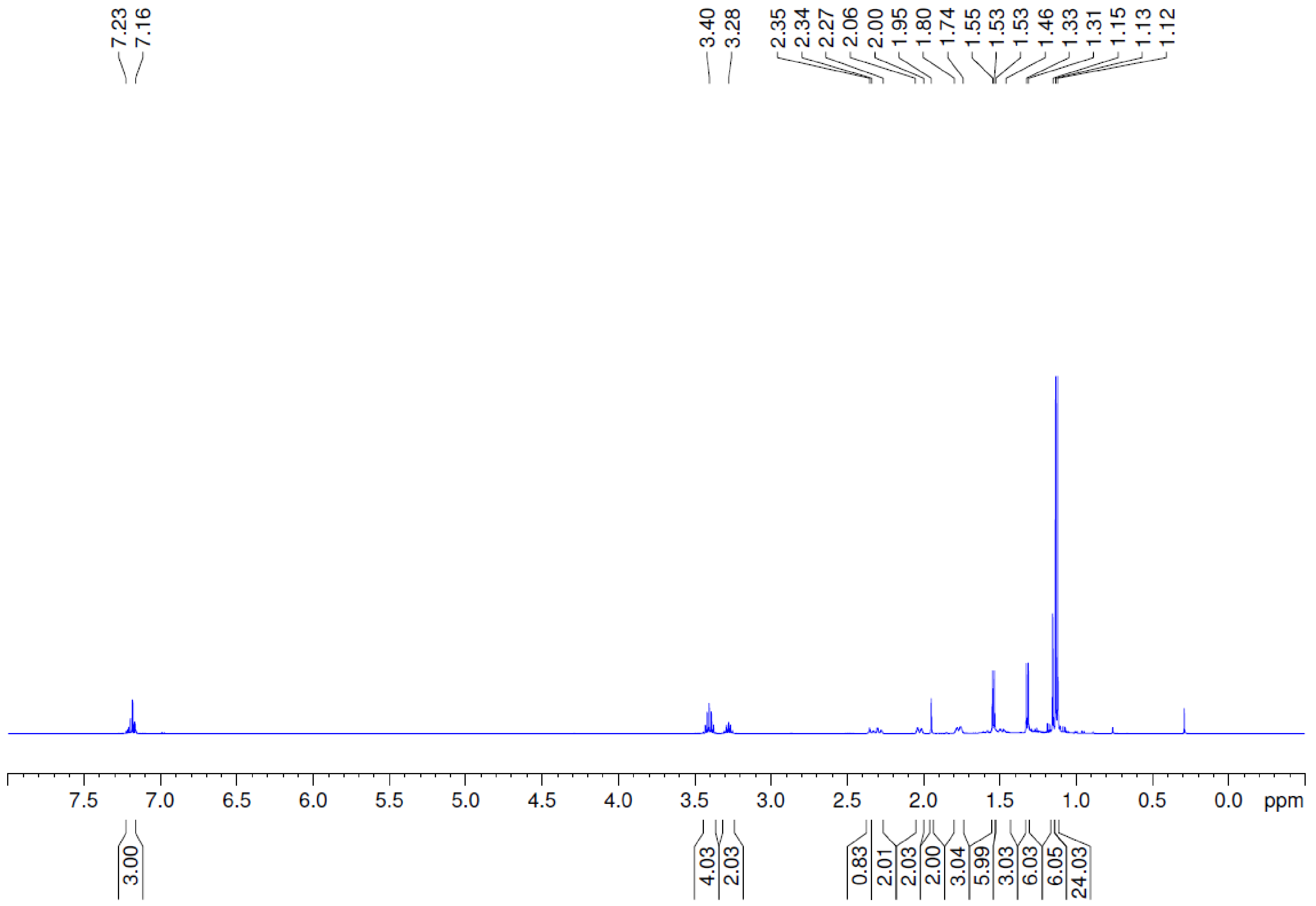

Figure S53. $^{1}H\{^{11}B\}$ NMR spectrum of **5b**$^{\mathbf{N}i\mathbf{Pr2}}$ in C_6D_6. The additional resonances correspond to **1b** (2%).

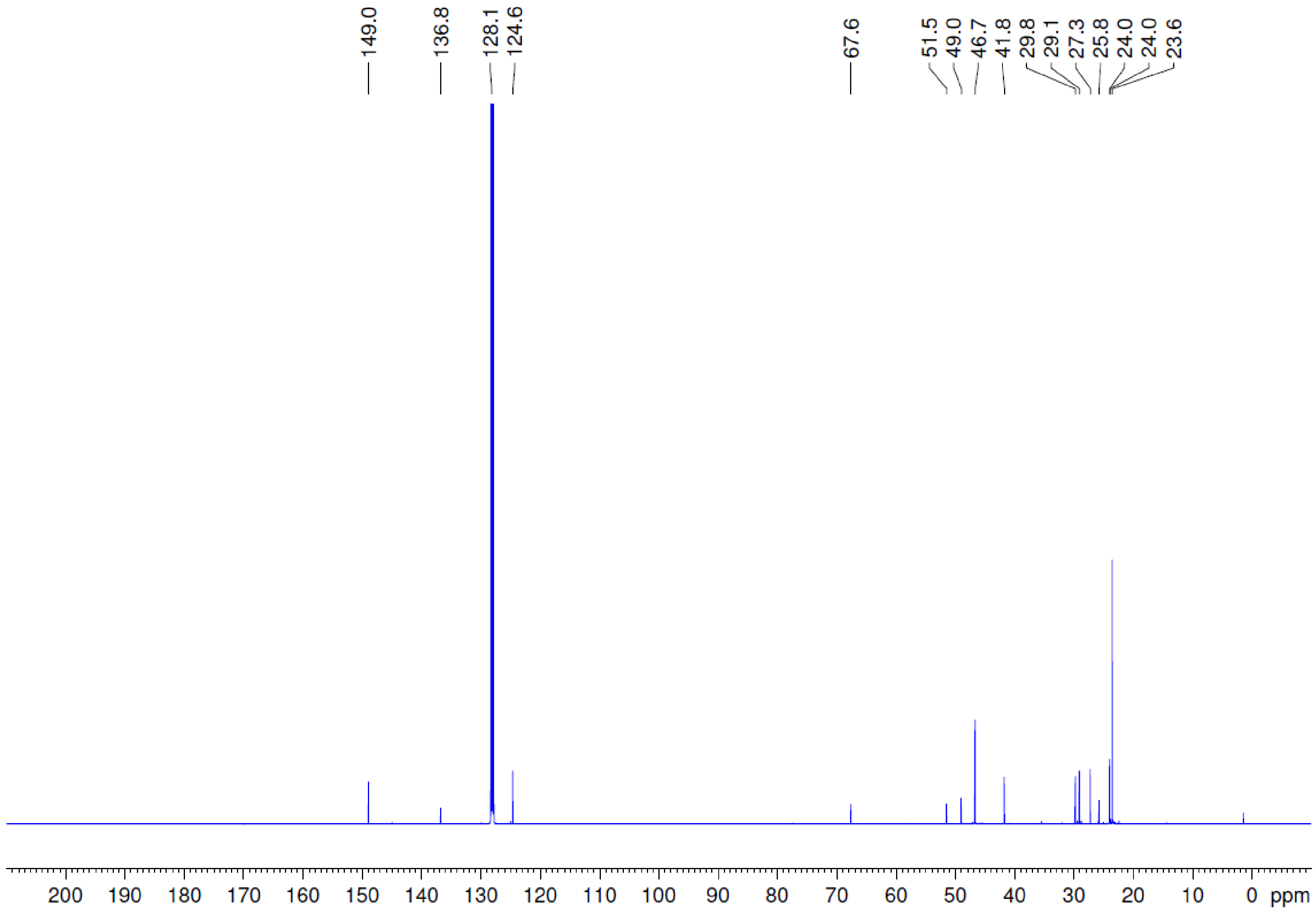

Figure S54. $^{13}C\{^{1}H\}$ NMR spectrum of **5b$^{\mathbf{N}\boldsymbol{i}\mathbf{Pr2}}$** in C_6D_6.

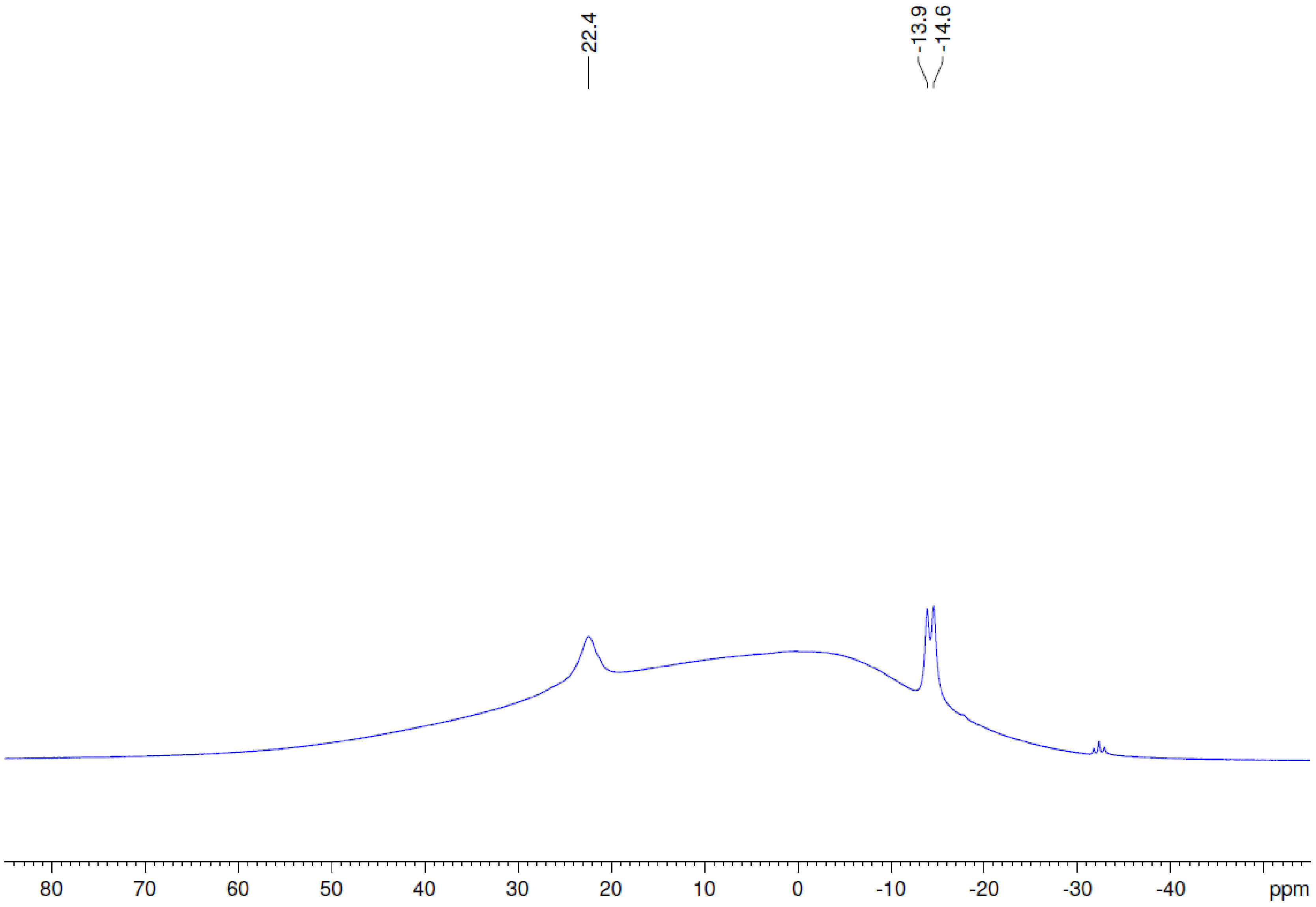

Figure S55. ^{11}B NMR spectrum of **5b$^{\mathbf{N}i\mathbf{Pr2}}$** in C_6D_6. The additional resonance at –32.4 ppm corresponds to **1b** (2%).

NMR spectrum of the reaction of 2a with 1,2-dibromopropane

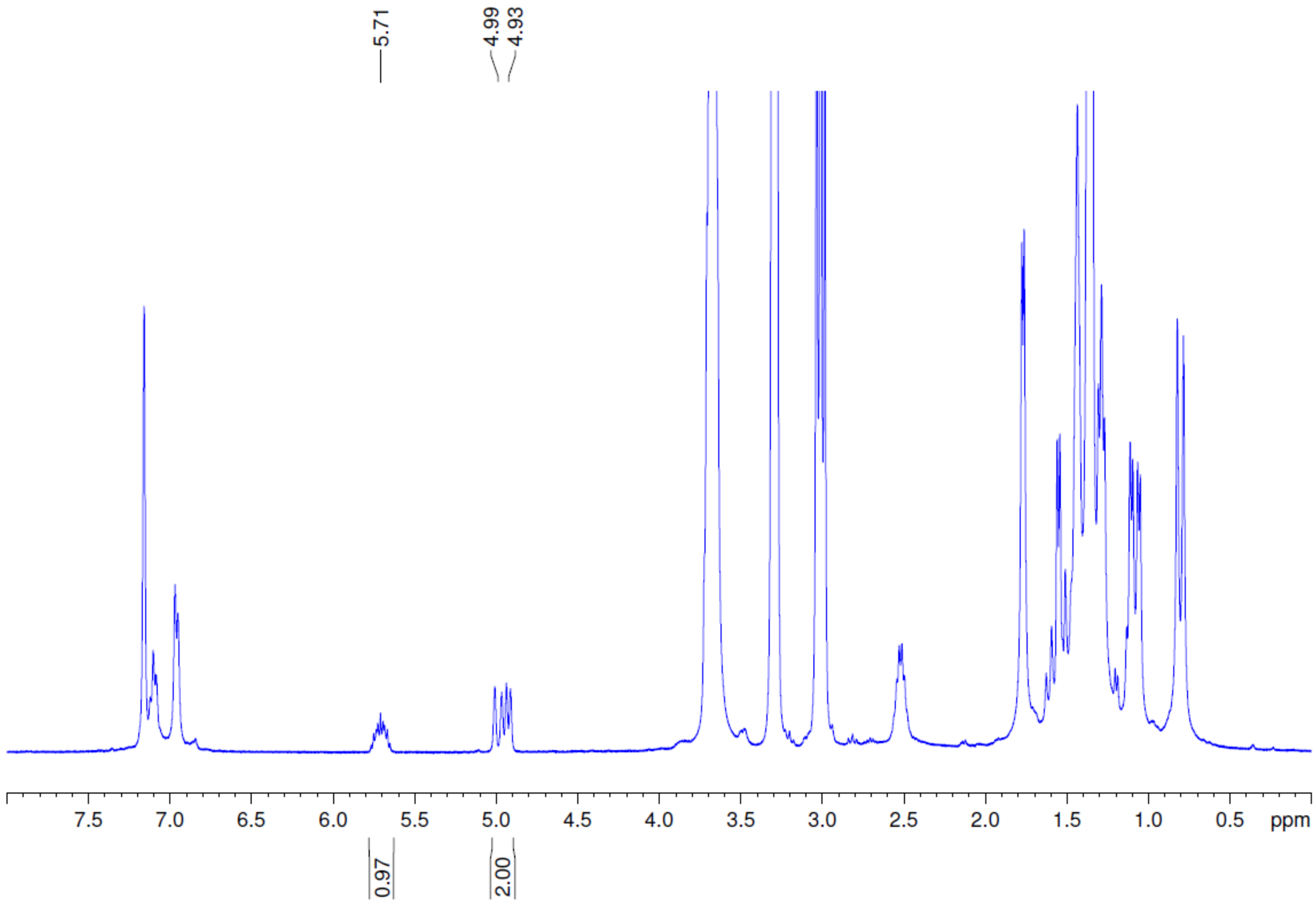

Figure S56. ^{1}H NMR spectrum of **2a** with 1,2-dibromopropane in C_6D_6.

UV-vis spectra

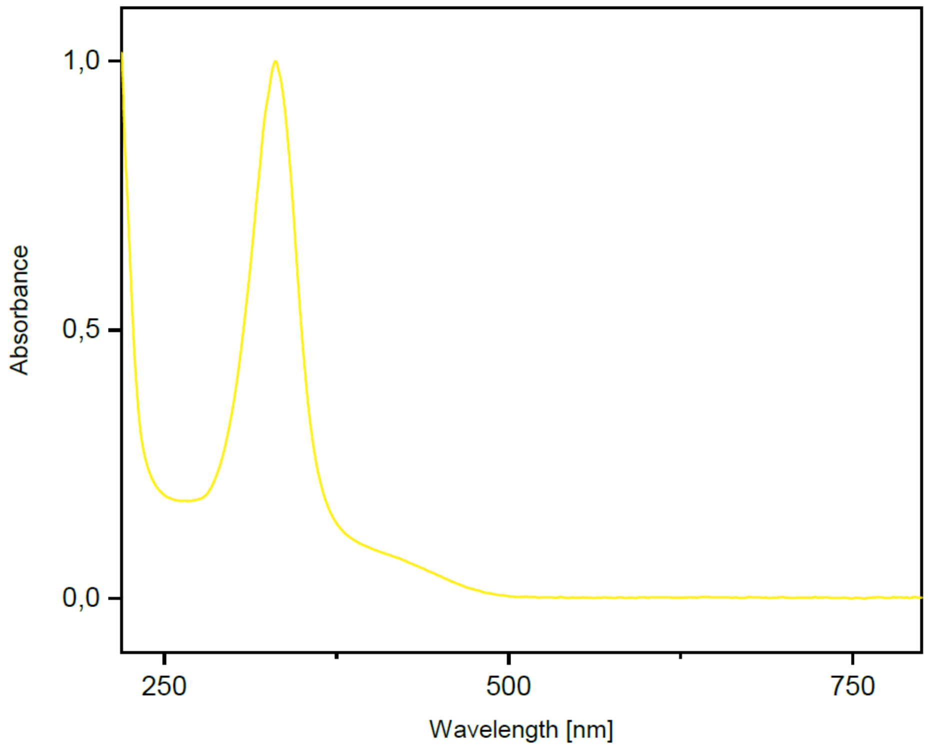

Figure S57. UV-vis absorption spectrum of compound **2b-TMEDA** in THF at 25 °C.

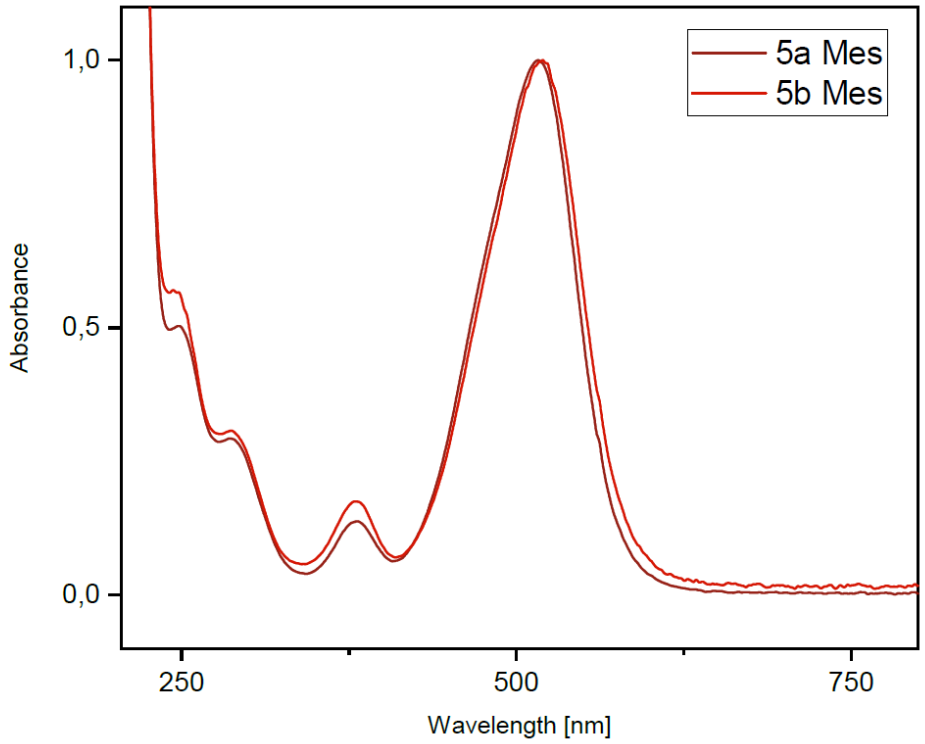

Figure S58. UV-vis absorption spectra of compounds **5a**$^{\mathbf{Mes}}$ (λ^1_{max} = 516 nm, λ^2_{max} = 381 nm) and **5b**$^{\mathbf{Mes}}$ (λ^1_{max} = 520 nm, λ^2_{max} = 380 nm) in hexane at 25 °C.

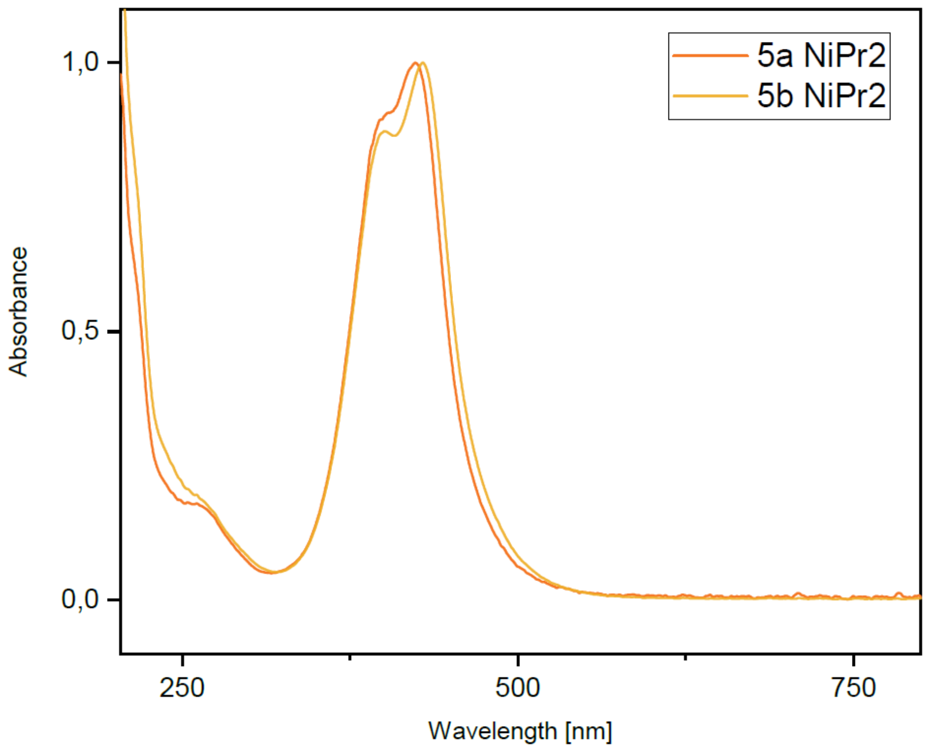

Figure S59. UV-vis absorption spectra of compounds **5a**$^{\mathbf{N}\boldsymbol{i}\mathbf{Pr2}}$ (λ^1_{max} = 423 nm, λ^2_{max} = 403 nm) and **5b**$^{\mathbf{N}\boldsymbol{i}\mathbf{Pr2}}$ (λ^1_{max} = 429 nm, λ^2_{max} = 401 nm) in hexane at 25 °C.

X-ray crystallographic data

The crystal data of **2b-TMEDA**, **3b**, **4a$^{\mathbf{COPh}}$**, **4b$^{\mathbf{COPh}}$**, **4a$^{\mathbf{CH2Cl}}$**, **4b$^{\mathbf{CH2Cl}}$**, **4a$^{\mathbf{C3H5}}$**, **4b$^{\mathbf{C3H5}}$**, **4a$^{\mathbf{Br}}$**, **4b$^{\mathbf{Br}}$**, **5b$^{\mathbf{Mes}}$**, **5a$^{\mathbf{N}i\mathbf{Pr2}}$** and **5b$^{\mathbf{N}i\mathbf{Pr2}}$** were collected on a *XtaLAB Synergy* diffractometer with a Hybrid Pixel Array detector and a multi-layer mirror monochromated $Cu_{K\alpha}$ radiation. The crystal data of **1b**, **2b** and **4a$^{\mathbf{COMe}}$** were collected on a BRUKER X8-APEX II diffractometer with a CCD area detector with a multi-layer mirror monochromated $Mo_{K\alpha}$ radiation. The crystal data of **4b$^{\mathbf{COMe}}$**, **4a$^{n\mathbf{Bu}}$** and **5a$^{\mathbf{Mes}}$** were collected on a BRUKER D8 QUEST diffractometer with a CMOS area detector with a multi-layer mirror monochromated $Mo_{K\alpha}$ radiation.

The structures were solved using the intrinsic phasing method,[5] refined with the SHELXL program,[6] and expanded using Fourier techniques. All non-hydrogen atoms were refined anisotropically. Hydrogen atoms were included in structure factor calculations. All hydrogen atoms except those attached to boron were assigned to idealized positions. The coordinates of the latter were refined freely.

Crystallographic data have been deposited with the Cambridge Crystallographic Data Centre as supplementary publication nos. CCDC 2065319-2065337. These data can be obtained free of charge from The Cambridge Crystallographic Data Centre *via* www.ccdc.cam.ac.uk/data_request/cif.

Refinement details for 1b: The CAAC backbone was modelled as twofold flip-disordered in C2, C3, C5, C6, in a 92:8 ratio. ADPs within this disorder were restrained to similarity with SIMU 0.005. The CN group was modelled as twofold disordered by rotation about the C1-B1 axis in a 38:64 ratio. ADPs within this disorder were restrained to similarity with SIMU 0.005. 1,2 and 1,3 distances for B1, C21, N2 were restrained to similarity with SAME.

Crystal data for 1b: $C_{24}H_{37}BN_2$, $M_r = 364.36$, colorless plate, 0.271×0.161×0.08 mm^3, monoclinic space group $P2_1/n$, $a = 9.5528(7)$ Å, $b = 21.3943(13)$ Å, $c = 10.8457(7)$ Å, $\beta = 96.131(3)°$, $V = 2203.9(3)$ Å^3, $Z = 4$, $\rho_{calcd} = 1.098$ g·cm^{-3}, $\mu = 0.063$ mm^{-1}, $F(000) = 800$, $T = 100(2)$ K, $R_1 = 0.0536$, $wR^2 = 0.1215$, 4934 independent reflections [$2\theta \leq 54.532°$] and 307 parameters.

Refinement details for 2b: Reflection data were very weak and had to be cut at 0.82 Å. The THF molecule centered on O1 presents a 69:31 disorder, the ADPs of which were restrained with SIMU 0.01. The cyano carbo atom C24 was oblong therefore its ADP was restrained with ISOR 0.001.

Crystal data for 2b: $C_{36}H_{60}BLiN_2O_3$, M_r = 586.61, yellow plate, 0.249×0.137×0.074 mm^3, triclinic space group $P\bar{1}$, a = 10.9375(16) Å, b = 11.4247(17) Å, c = 14.794(2) Å, α = 79.934(4)°, β = 87.130(4)°, γ = 76.707(4)°, V = 1771.3(4) Å^3, Z = 2, ρ_{calcd} = 1.100 g·cm^{-3}, μ = 0.067 mm^{-1}, F(000) = 644, T = 103(2) K, R_1 = 0.0989, wR^2 = 0.1601, 6638 independent reflections [$2\theta \leq 51.358°$] and 444 parameters.

Refinement details for 2b-TMEDA: The TMEDA ligand showed a twofold disorder refined to a 75:25 ratio, except in N4. The atomic displacement parameters of the TMEDA carbon and nitrogen atoms (residues 2 and 12) were restrained to the same value with similarity restraint SIMU 0.005.

Crystal data for 2b-TMEDA: $[C_{30}H_{52}BLiN_4]_2$, M_r = 973.01, yellow plate, 0.246×0.139×0.094 mm^3, monoclinic space group $P2_1/c$, a = 12.39117(17) Å, b = 17.1388(2) Å, c = 15.3466(2) Å, β = 107.1486(16)°, V = 3114.26(9) Å^3, Z = 2, ρ_{calcd} = 1.038 g·cm^{-3}, μ = 0.446 mm^{-1}, F(000) = 1072, T = 99.9(4) K, R_1 = 0.0496, wR^2 = 0.1250, 6135 independent reflections [$2\theta \leq 144.218°$] and 415 parameters.

Refinement details for 3b: The unit cell contains highly disordered and partially occupied solvent molecules (hexane) which have been treated as a diffuse contribution to the overall scattering without specific atom positions by SQUEEZE/PLATON.[7] 569 electrons were thus squeezed from the unit cell, corresponding to 11.4 hexane molecules. Three reflections affected by the beamstop were omitted. The CAAC ligand at B2 was modelled as twofold disordered in both the Dip (RESI 21 and 22) and Cy (RESI 11 and 12) residues in a 63:37 and 69:31 ratio, respectively. ADPs in RESI 11 and 12 Cy were restrained with ISOR 0.005. ADPs in RESI 21 and 22 were restrained with SIMU 0.005 and those of C39_22 and C40_22 additionally with ISOR 0.001. The N3-C39_21 and N3-C39_22 bond lengths were restrained to similarity with SADI 0.005. The backbone of the CAAC ligand (RESI 31 and 32 CAAC) and one adjacent *i*Pr group (RESI 41 and 42 *i*Pr) were modelled as twofold disordered in a 39:61 ratio. 1,2 and 1,3

distances in RESI 21 and 22 were restrained to similarity with SAME. ADPs in RESI 31 and 32 were restrained with SIMU 0.01 and ISOR 0.005, those in RESI 41 and 42 with SIMU 0.01.

Crystal data for 3b: $C_{144}H_{218}B_6Li_4N_{12}$, M_r = 2209.91, orange block, 0.309×0.192×0.175 mm^3, space group *C*2/*c*, *a* = 29.61598(19) Å, *b* = 18.26402(11) Å, *c* = 29.16023(19) Å, β = 100.8608(6) °, *V* = 15490.41(18) Å^3, *Z* = 4, ρ_{calcd} = 0.948 g·cm^{-3}, μ = 0.399 mm^{-1}, *F*(000) = 4832, *T* = 102(2) K, R_1 = 0.0523, wR^2 = 0.1471, 15228 independent reflections [2θ≤144.258°] and 985 parameters.

Crystal data for 4a$^{\mathbf{COMe}}$: $C_{23}H_{35}BN_2O$, M_r = 366.34, colorless block, 0.384×0.33×0.252 mm^3, triclinic space group $P\bar{1}$, *a* = 9.696(5) Å, *b* = 9.9096(15) Å, *c* = 13.612(2) Å, α = 95.654(9)°, β = 104.299(7)°, γ = 114.996(18)°, *V* = 1117.6(6) Å^3, *Z* = 2, ρ_{calcd} = 1.089 g·cm^{-3}, μ = 0.065 mm^{-1}, *F*(000) = 400, *T* = 100 K, R_1 = 0.0487, wR^2 = 0.1275, 4392 independent reflections [2θ≤52.042°] and 256 parameters.

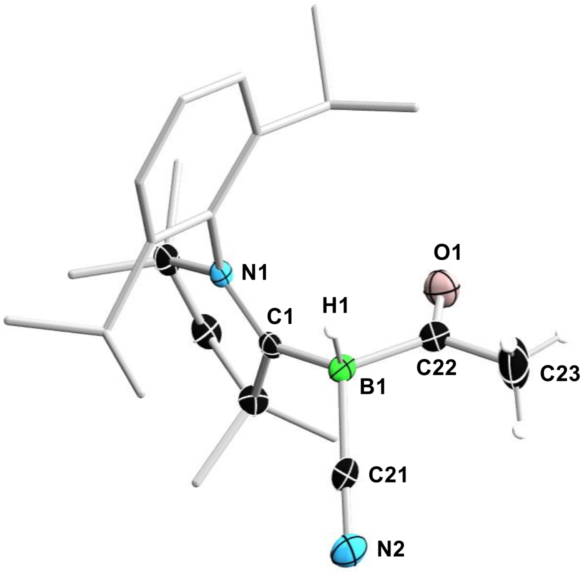

Figure S60. Crystallographically-derived solid-state structure of **4a$^{\mathbf{COMe}}$**. Atomic displacement ellipsoids drawn at 50% probability. Ellipsoids of the carbon atoms on ligand periphery and hydrogen atoms omitted for clarity, except for the boron-bound hydride and those of the acetyl group.

Crystal data for 4b$^{\mathbf{COMe}}$: $C_{26}H_{39}BN_2O$, M_r = 406.40, colorless block, 0.553×0.385×0.241 mm^3, monoclinic space group *P*2$_1$/*n*, *a* = 9.495(4) Å, *b* = 15.068(7) Å, *c* = 17.244(3) Å, β = 91.601(19)°, *V* = 2466.0(15) Å^3, *Z* = 4, ρ_{calcd} = 1.095 g·cm^{-3},

$\mu = 0.065\ mm^{-1}$, $F(000) = 888$, $T = 100(2)$ K, $R_1 = 0.0418$, $wR^2 = 0.1032$, 4845 independent reflections [$2\theta \leq 52.044°$] and 282 parameters.

Crystal data for 4a^{COPh}: $C_{60}H_{84}B_2N_4O_3$, $M_r = 930.93$, colorless block, 0.293x0.227x0.215 mm^3, monoclinic space group $P2_1$, $a = 10.34309(5)$ Å, $b = 14.60042(7)$ Å, $c = 18.24313(9)$ Å, $\beta = 95.5560(4)$ °, $V = 2742.01(2)$ Å^3, $Z = 2$, $\rho_{calcd} = 1.128$ g·cm^{-3}, $\mu = 0.520\ mm^{-1}$, $F(000) = 1012$, $T = 100.00(10)$ K, $R_1 = 0.0322$, $wR^2 = 0.0822$, 11603 independent reflections [$2\theta \leq 155.418°$] and 647 parameters.

Refinement details for 4b^{COPh}: The unit cell contains solvent molecules which have been treated as a diffuse contribution to the overall scattering without specific atom positions by Squeeze/Platon.[7] 108 electrons were thus squeezed from the unit cell, i.e. ca. two molecules of hexane.

Crystal data for 4b^{COPh}: $C_{31}H_{41}BN_2O$, $M_r = 468.47$, colorless plate, 0.216×0.096×0.033 mm^3, orthorhombic space group $P2_12_12_1$, $a = 9.2711(9)$ Å, $b = 16.3277(17)$ Å, $c = 20.1460(14)$ Å, $V = 3049.6(5)$ Å^3, $Z = 4$, $\rho_{calcd} = 1.020$ g·cm^{-3}, $\mu = 0.460$ mm^{-1}, $F(000) = 1016$, $T = 100.00(10)$ K, $R_1 = 0.0836$, $wR^2 = 0.2303$, 5785 independent reflections [$2\theta \leq 144.230°$] and 326 parameters.

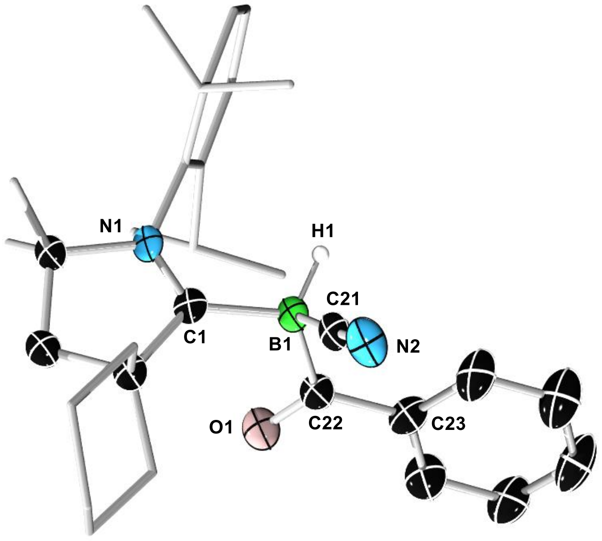

Figure S61. Crystallographically-derived solid-state structure of **4b^{COPh}**. Atomic displacement ellipsoids drawn at 50% probability. Ellipsoids of the carbon atoms on the ligand periphery and hydrogen atoms omitted for clarity, except for the boron-bound hydride.

Crystal data for 4a^{CH2Cl}: $C_{22}H_{34}BClN_2$, M_r = 372.77, colorless block, 0.220x0.156×0.098 mm^3, monoclinic space group $P2_1/n$, a = 9.54287(9) Å, b = 17.40295(18) Å, c = 13.13043(15) Å, β = 102.6097(10)°, V = 2128.02(4) Å^3, Z = 4, ρ_{calcd} = 1.164 g·cm^{-3}, μ = 1.622 mm^{-1}, F(000) = 808, T = 99.98(16) K, R_1 = 0.0413, wR^2 = 0.1157, 4190 independent reflections [$2\theta \leq 144.25°$] and 246 parameters.

Refinement details for 4b^{CH2Cl}: All hydrogen atoms except H1 were assigned to idealized positions. The coordinates of H1 were refined freely. The asymmetric unit contains half a benzene molecule on an inversion centre, which was modelled as twofold disordered in a 79:21 ratio. The benzene rings were idealized with AFIX 66 and ADPs restrained with ISOR 0.005 and SIMU 0.005.

Crystal data for 4b^{CH2Cl}: $C_{28}H_{41}BClN_2$, M_r = 451.89, colorless block, 0.158×0.115×0.067 mm^3, triclinic space group $P\bar{1}$, a = 9.68680(10) Å, b = 9.8863(2) Å, c = 15.4954(2) Å, α = 96.7660(10)°, β = 92.7870(10)°, γ = 116.289(2)°, V = 1312.70(4) Å^3, Z = 2, ρ_{calcd} = 1.143 g·cm^{-3}, μ = 1.397 mm^{-1}, F(000) = 490, T = 105(5) K, R_1 = 0.0621, wR^2 = 0.1763, 5148 independent reflections [$2\theta \leq 144.254°$] and 356 parameters.

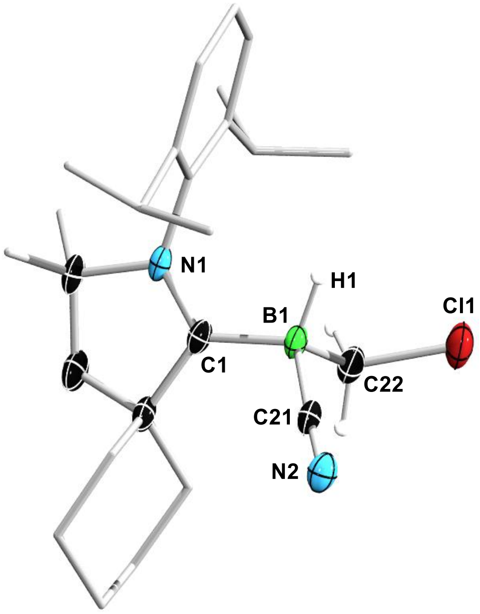

Figure S62. Crystallographically-derived solid-state structure of **4b^{CH2Cl}**. Atomic displacement ellipsoids drawn at 50% probability. Ellipsoids of the carbon atoms on ligand periphery and hydrogen atoms omitted for clarity, except for the boron-bound hydride and those of the allyl group.

Refinement details for 4a^{nBu}: All non-hydrogen atoms were refined anisotropically. Hydrogen atoms were included in the structure factor calculations. All hydrogen atoms except H1, H2, H3 and H4 were assigned to idealized positions. The coordinates of H1, H2, H3 and H4 bound to the boron atoms were refined freely. The crystal was a non-pseudo-merohedral twin with domains rotated by 180.0° around real axis [0.048 1.000 0.011]. The BASF parameter was refined to 28%. The most disagreeable reflections (4 11 10; -1 -11 24) were omitted.

Crystal data for 4a^{nBu}: $C_{25}H_{41}BN_2$, M_r = 380.41, colorless block, 0.517×0.158×0.154 mm^3, triclinic space group $P\bar{1}$, a = 9.973(3) Å, b = 17.902(4) Å, c = 26.609(5) Å, α = 91.068(7)°, β = 91.141(7)°, γ = 91.495(6)°, V = 4747.0(19) Å^3, Z = 8, ρ_{calcd} = 1.065 g·cm^{-3}, μ = 0.060 mm^{-1}, F(000) = 1680, T = 100(2) K, R_1 = 0.0597, wR^2 = 0.1403, 17546 independent reflections [$2\theta \leq 50.504°$] and 1062 parameters.

Crystal data for 4a^{C3H5}: $C_{24}H_{37}BN_2$, M_r = 364.36, colorless block, 0.246x0.198x0.145 mm^3, monoclinic space group $I12/a1$, a = 18.0912(2) Å, b = 9.93388(10) Å, c = 26.4542(3) Å, β = 109.0857(13)°, V = 4492.91(9) Å^3, Z = 8, ρ_{calcd} = 1.077 g·cm^{-3}, μ = 0.457 mm^{-1}, F(000) = 1600, T = 100.00(10) K, R_1 = 0.0437, wR^2 = 0.1193, 4420 independent reflections [$2\theta \leq 144.244°$] and 255 parameters.

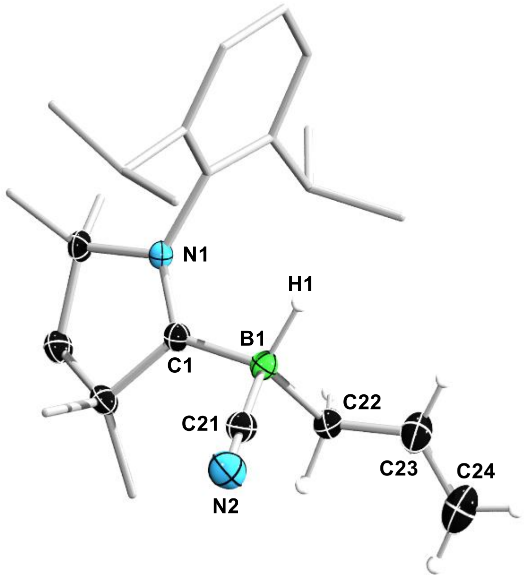

Figure S63. Crystallographically-derived solid-state structure of **4a^{C3H5}**. Atomic displacement ellipsoids drawn at 50% probability. Ellipsoids of the carbon atoms on the ligand periphery and hydrogen atoms omitted for clarity, except for the boron-bound hydride and those of the allyl group.

Crystal data for 4b^{C3H5}: $C_{27}H_{41}BN_2$, *M*r = 404.43, colorless block, 0.246x0.198x0.145 mm^3, monoclinic space group P2$_1$/*n*, a = 8.81342(9) Å, b = 17.05128(19) Å, c = 16.6957(2) Å, β = 103.4565(11)°, *V* = 2440.15(5) Å3, *Z* = 4, ρ_{calcd} = 1.101 g·cm^{-3}, μ = 0.466 mm^{-1}, *F*(000) = 888, *T* = 100.00(10) K, R_1 = 0.0446, wR^2 = 0.1206, 4811 independent reflections [2θ≤144.244°] and 281 parameters.

Refinement details for 4a^{Br}: All hydrogen atoms except H1 were assigned to idealized positions. The coordinates of H1 were refined freely. The Br and CN ligands were modelled as twofold disordered by reflection through the C1-B1-H1 plane refined to a 55:45 ratio. ADPs within the disordered parts were restrained to similarity with B1 using SIMU 0.01. The CN bond lengths were restrained with DFIX 1.16 0.005.

Crystal data for 4a^{Br}: $C_{21}H_{32}BBrN_2$, M_r = 403.20, colorless block, 0.252×0.118×0.115 mm^3, monoclinic space group *P*2$_1$/*n*, *a* = 9.5611(2) Å, *b* = 17.5409(3) Å, *c* = 12.6623(2) Å, α = 90°, β = 101.043(2)°, γ = 90°, *V* = 2084.28(7) Å^3, *Z* = 4, ρ_{calcd} = 1.285 g·cm^{-3}, μ = 2.705 mm^{-1}, *F*(000) = 848, *T* = 100.0(3) K, R_1 = 0.0722, wR^2 = 0.1956, 4430 independent reflections [2θ≤155.786°] and 266 parameters.

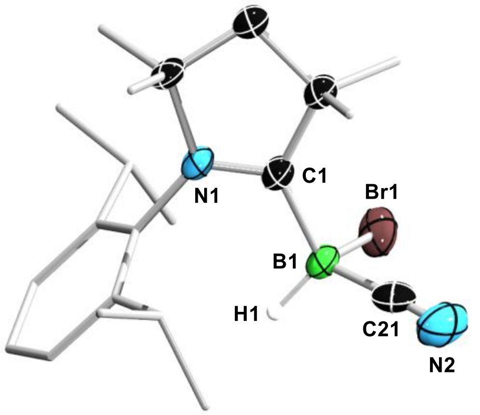

Figure S64. Crystallographically-derived solid-state structure of **4a^{Br}**. Atomic displacement ellipsoids drawn at 50% probability. Ellipsoids of the carbon atoms on the ligand periphery and hydrogen atoms omitted for clarity, except for the boron bound hydride.

Refinement details for 4b^{Br}: All hydrogen atoms except H1 were assigned to idealized positions. The coordinates of H1 were refined freely. The Br and CN ligands were modelled as twofold disordered by reflection through the C1-B1-H1 plane refined to a 81:19 ratio. ADPs

within the disordered parts were restrained to similarity with B1 using SIMU 0.003. The CN bond lengths were restrained with DFIX 1.16 0.005. Oblong ellipsoids in the aryl ring (C3_3 > C5_3) were restrained with ISOR 0.002.

Crystal data for 4b^{Br}: $C_{24}H_{36}BBrN_2$, M_r = 443.27, colorless plate, 0.378×0.182×0.092 mm^3, monoclinic space group $P2_1$, a = 9.30880(10) Å, b = 10.24510(10) Å, c = 11.97960(10) Å, β = 96.3960(10)°, V = 1135.378(19) Å^3, Z = 2, ρ_{calcd} = 1.297 g·cm^{-3}, μ = 2.531 mm^{-1}, F(000) = 468, T = 100.0(3) K, R_1 = 0.0302, wR^2 = 0.0811, Flack parameter = 0.025(11), 4810 independent reflections [$2\theta \leq 155.094°$] and 291 parameters.

Crystal data for 5a^{Mes}: $C_{39}H_{54}B_2N_2$, M_r = 572.46, pink block, 0.305×0.281×0.251 mm^3, monoclinic space group $P2_1/c$, a = 23.5259(14) Å, b = 9.318(3) Å, c = 16.408(9) Å, β = 97.100(15)°, V = 3569(2) Å^3, Z = 4, ρ_{calcd} = 1.065 g·cm^{-3}, μ = 0.060 mm^{-1}, F(000) = 1248, T = 100(2) K, R_1 = 0.0401, wR^2 = 0.1040, 6962 independent reflections [$2\theta \leq 52.042°$] and 406 parameters.

Crystal data for 5b^{Mes}: $C_{42}H_{58}B_2N_2$, M_r = 612.52, red block, 0.400×0.324×0.216 mm^3, monoclinic space group $P2_1/n$, a = 10.05638(6) Å, b = 34.29758(17) Å, c = 11.37023(6) Å, β = 108.8828(6)°, V = 3710.64(6) Å^3, Z = 4, ρ_{calcd} = 1.096 g·cm^{-3}, μ = 0.457 mm^{-1}, F(000) = 1336, T = 101(1) K, R_1 = 0.0386, wR^2 = 0.1008, 7310 independent reflections [$2\theta \leq 144.252°$] and 430 parameters.

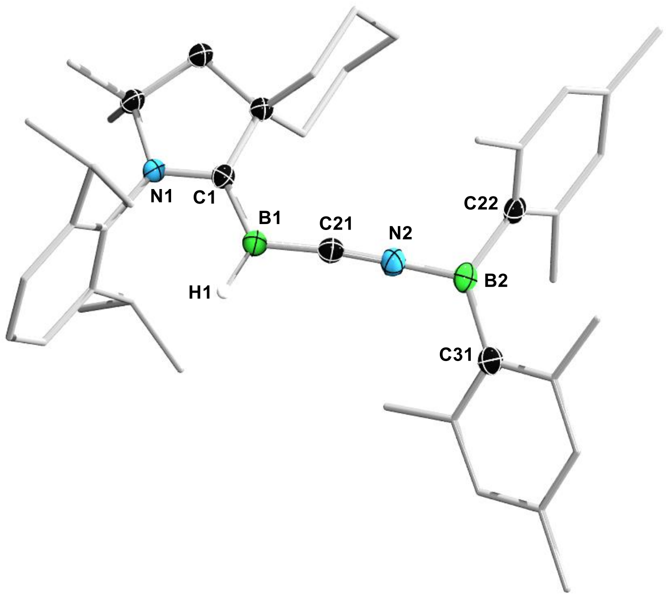

Figure S65. Crystallographically-derived solid-state structure of **5b$^{\mathbf{Mes}}$**. Atomic displacement ellipsoids drawn at 50% probability. Ellipsoids of the carbon atoms on the ligand periphery and hydrogen atoms omitted for clarity, except for the boron bound hydride.

Refinement details for 5a$^{\mathbf{NiPr2}}$: One isopropyl group (RESI 2 and 12 IPR) of the N*i*Pr_2 moiety showed a twofold disorder refined to a 75:25 ratio. The atomic displacement parameters of the atoms were restrained to the same value with the similarity restraint SIMU 0.005. The CAAC ligand (RESI 3 and 13 CAAC) was modelled with a twofold disorder refined to a 79:21 ratio in C1 > C8. ADPs within the disorder were restrained to similarity with SIMU 0.005.

Crystal data for 5a$^{\mathbf{N\textit{i}Pr2}}$: $C_{33}H_{60}B_2N_4$, M_r = 534.47, orange block, 0.310×0.253×0.088 mm^3, monoclinic space group $P2_1/c$, a = 14.33296(15) Å, b = 13.74944(12) Å, c = 17.9845(2) Å, β = 100.8604(11)°, V = 3480.73(7) $Å^3$, Z = 4, ρ_{calcd} = 1.020 g·cm^{-3}, μ = 0.435 mm^{-1}, F(000) = 1184, T = 100.0(2) K, R_1 = 0.0410, wR^2 = 0.1078, 6849 independent reflections [$2\theta \leq$ 144.238°] and 488 parameters.

Refinement details for 5b$^{\mathbf{N\textit{i}Pr2}}$: The CAAC ligand (RESI 2 and 12 CAAC) was modelled with a twofold disorder refined to a 83:17 ratio in C1 > C10. ADPs within the disorder were restrained to similarity with SIMU 0.005.

Crystal data for 5b$^{\mathbf{N}i\mathbf{Pr2}}$: $C_{36}H_{64}B_2N_4$, M_r = 574.53, orange block, 0.300× 0.255×0.133 mm^3, triclinic space group $P\,\bar{1}$, a = 9.45643(19) Å, b = 13.98764(18) Å, c = 15.3030(3) Å, α = 81.2587(14)°, β = 75.3574(18)°, γ = 70.8501(15)°, V = 1844.76(7) Å^3, Z = 2, ρ_{calcd} = 1.034 g·cm^{-3}, μ = 0.439 mm^{-1}, F(000) = 636, T = 100.0(2) K, R_1 = 0.0454, wR^2 = 0.1251, 7277 independent reflections [$2\theta\leq$144.210°] and 490 parameters.

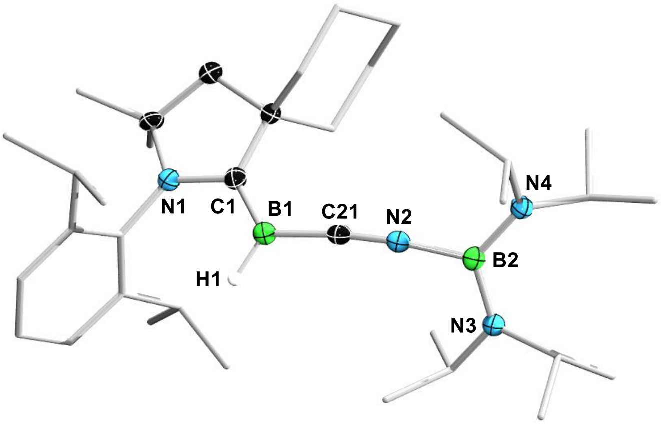

Figure S66. Crystallographically-derived solid-state structure of **5b**$^{\mathbf{N}i\mathbf{Pr2}}$. Atomic displacement ellipsoids drawn at 50% probability. Ellipsoids of the carbon atoms on the ligand periphery and hydrogen atoms omitted for clarity, except for the boron bound hydride.

Computational details

Geometry optimizations and Hessian calculations were performed for **5a**$^{\mathbf{Mes}}$ and **5a**$^{\mathbf{N}\boldsymbol{i}\mathbf{Pr2}}$ using the following levels of theory: B3LYP/def2-SVP[8] and PBE0-D3/def2-SVP.[9] Both optimized structures were characterized as minima based on all positive eigenvalues in vibrational analysis. Additionally single-point calculations over the optimized structures with basis sets of triple-ζ quality using the double-hybrids method were performed at B3LYP/def2-SVP//DSDPBEP86/6-311++G(d,p) level.[10] The canonical Kohn-Sham HOMO and LUMO orbitals of **5a**$^{\mathbf{Mes}}$ and **5a**$^{\mathbf{N}\boldsymbol{i}\mathbf{Pr2}}$ for both DFT methods describe the N-C-B-C-N-B π-systems, and their HOMO-LUMO gaps are 0.1020 and 0.1179 eV, respectively, for B3LYP and 0.1134 and 0.1335 eV, respectively, for PBE0-D3. On correlated levels the LUMO to LUMO–3 orbitals of **5a** $^{\mathbf{N}\boldsymbol{i}\mathbf{Pr2}}$ are localized on the periphery of the molecule. A similarly shaped orbital to those obtained with DFT methods was LUMO−4. For both compounds the HOMO–LUMO gaps at correlated levels were much larger than those obtained with standard DFT methods: 0.2086 and 0.2150 eV, respectively. The above-mentioned calculations were performed using Gaussian 16, revision B.01,[11] and illustrations of the orbitals were prepared with Molekel 4.3.[12] Bond orders were computed using Natural Resonance Theory Analysis, as included in NBO 7,[13] using the B3LYP/def2-SVP and PBE0-D3/def2-SVP wave functions resulting from Gaussian 16 calculations. Both DFT methods gave qualitatively similar values for the bond orders for each compound.

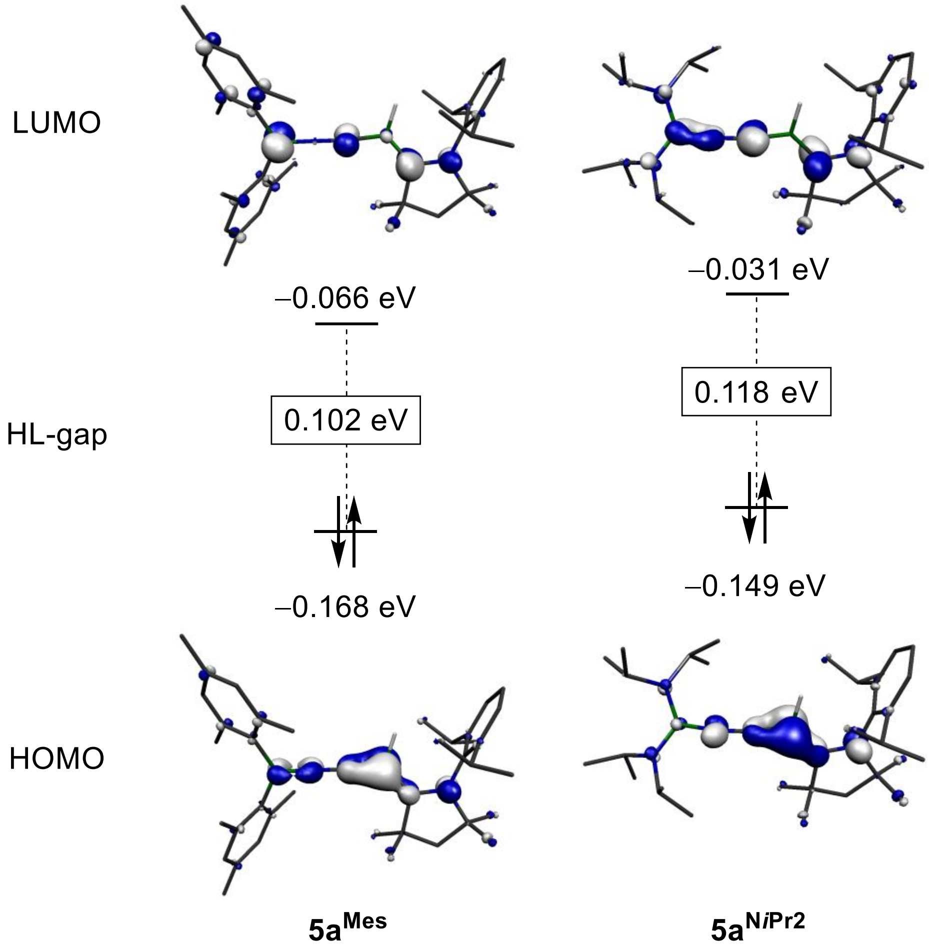

Figure S67. Canonical Kohn-Sham molecular orbitals of **5a^{Mes}** and **5a^{NiPr2}** at the B3LYP/def2-SVP level of theory.

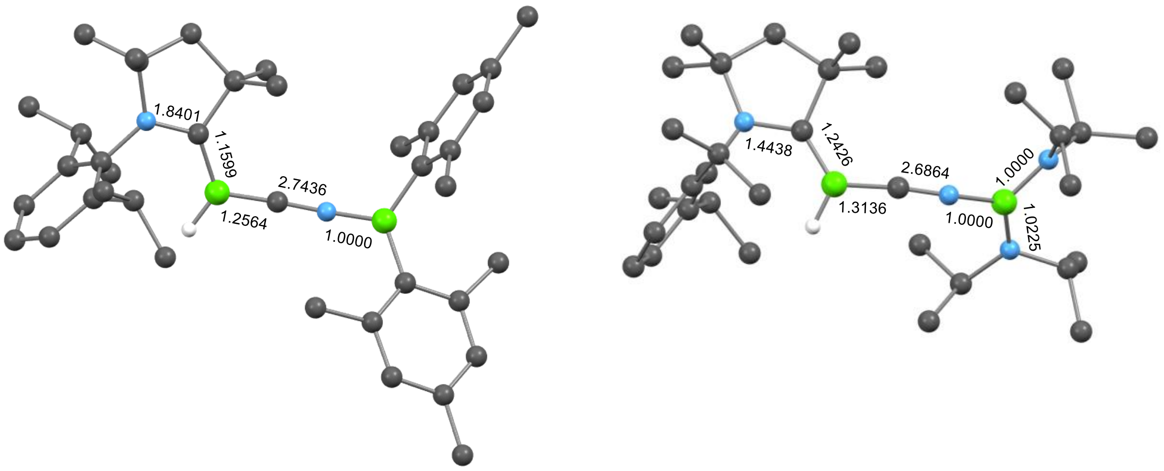

Figure S68. NRT bond orders calculated for of **5a^{Mes}** and **5a^{NiPr2}** at the B3LYP/def2-SVP level of theory.

Table S1. Optimized cartesian coordinates of **5a^{Mes}** (left B3LYP/def2-SVP, right PBE0-D3/Def2-SVP).

C	1.364872171502	0.306859789092	-1.524675883355	C	1.370582786094	0.437663942802	-1.260084028966
N	2.592750964230	0.171993777412	-2.092879907328	N	2.481581959715	0.291718689058	-2.009124602755
C	0.413338422396	0.934668303251	-2.552095590204	C	0.265446142799	1.007660073661	-2.142028975012
C	-0.549615862380	-0.141212432834	-3.10848999810	C	-0.721476855269	-0.113854063315	-2.506884947121
H	-1.170146233048	0.287294685517	-3.913135537111	H	-1.464395051016	0.259157873456	-3.230087683114
H	-1.218524929093	-0.508792122763	-2.31724652997	H	-1.258545617870	-0.459024242388	-1.611897140809
H	-0.013527440602	-1.009372111636	-3.51869049488	H	-0.216866261356	-0.984288437397	-2.950022107432
C	-0.423803103530	2.091842475573	-1.981141523255	C	-0.509942983937	2.152833703782	-1.492071891874
H	-0.972353156116	2.591967259883	-2.796823755581	H	-1.168413960119	2.626376442314	-2.237941121323
H	0.211958467142	2.842066006773	-1.486402664896	H	0.170084252426	2.920303177843	-1.092882466708
H	-1.167351857093	1.737554870285	-1.255328600692	H	-1.152684517334	1.797850605424	-0.676974547035
C	1.382037604746	1.463477056891	-3.639506977654	C	1.045211387347	1.519049715715	-3.368554679518
H	0.952989994174	1.397028790643	-4.650528896479	H	0.469037305335	1.425063575708	-4.300747638066
H	1.592930265042	2.527314176569	-3.447545574084	H	1.266539670749	2.588799910201	-3.228983801012
C	2.692843790151	0.661444893310	-3.517144289844	C	2.364834196837	0.741083596653	-3.425793124143
C	3.922655598185	1.550518928320	-3.745176548894	C	3.532813533921	1.632566600187	-3.838787193270
H	3.927122729937	1.891710498175	-4.792083675354	H	3.398960014730	1.939688107078	-4.886612172617
H	4.860037715991	1.002757499883	-3.567103958622	H	4.491626265508	1.098356478501	-3.761257121829
H	3.910325324000	2.441662962307	-3.105172614035	H	3.588281904039	2.541856857780	-3.227110400238
C	2.760350151092	-0.499865233030	-4.525943870331	C	2.311356814036	-0.443937516657	-4.391877819069
H	2.780908847317	-0.089412860543	-5.547438034712	H	2.212397697523	-0.070000607526	-5.421672896454
H	1.896172702742	-1.172744746732	-4.452777949353	H	1.462613158483	-1.110124995567	-4.192545229621
H	3.675585025703	-1.091692349747	-4.388225301715	H	3.237884102220	-1.031950749331	-4.334525750705
C	3.730179707797	-0.383351353916	-1.393327553526	C	3.683594132621	-0.294536623826	-1.499006399204
C	4.560185781479	0.480692056623	-0.630089118701	C	4.656513649607	0.542980927694	-0.911594211329
C	5.700441714593	-0.060466512716	-0.018858700704	C	5.853970904151	-0.035084820688	-0.479889016177
H	6.352855054371	0.591475493536	0.566255040497	H	6.620575672047	0.598496392520	-0.027369344046
C	6.009721635043	-1.413496823193	-0.126636776025	C	6.076288643352	-1.401798722069	-0.594634705704
H	6.907064547985	-1.812601483875	0.353331966430	H	7.021124903059	-1.834284241359	-0.256231649125
C	5.151705616168	-2.260169783811	-0.821718281351	C	5.077869779704	-2.221159259094	-1.105891499574
H	5.374206285488	-3.328786428627	-0.863328976703	H	5.236541141197	-3.301654399535	-1.143862245879
C	3.994405480846	-1.776767231819	-1.451309590708	C	3.860461829015	-1.694631016709	-1.549864302161
C	3.041786303738	-2.793843550302	-2.079967702905	C	2.759540025197	-2.655143914240	-1.965507978047
H	2.242184786301	-2.238789914895	-2.585495780505	H	1.919362766236	-2.057029524401	-2.341924928604
C	3.726341439918	-3.696142005657	-3.122185621986	C	3.195778509601	-3.618937786043	-3.069176101723
H	2.982552392508	-4.341764631702	-3.616932444936	H	2.343539607982	-4.232482618908	-3.400875788342
H	4.472231427536	-4.360041947269	-2.656299794980	H	3.975025583218	-4.311274828574	-2.713506030837
H	4.243236953095	-3.116873060273	-3.902240732727	H	3.597616601058	-3.093210518307	-3.947495155661
C	2.365556038562	-3.654810675180	-0.994202856878	C	2.239425233849	-3.431222961556	-0.751217583437
H	1.637768438997	-4.344879266250	-1.452366016643	H	1.404995153568	-4.087439443152	-1.046043946005
H	1.831941395795	-3.028136436768	-0.266097171373	H	1.878268705422	-2.746256331109	0.028184684967
H	3.104464375170	-4.263620877968	-0.447757038103	H	3.028681085801	-4.065472739908	-0.316623523454
C	4.231831344289	1.951370733443	-0.370638267081	C	4.411711223807	2.016708799847	-0.640120497484
H	3.385602803253	2.226568432271	-1.014659099089	H	3.479501401148	2.299250964665	-1.149723523020
C	3.767569741758	2.157537158330	1.084978226218	C	4.183075842487	2.246307188664	0.856820540624
H	3.480706204722	3.209295252460	1.250384826648	H	3.957525684469	3.306646561404	1.053154610522
H	4.571953221711	1.913740037391	1.798393802048	H	5.078764375245	1.977940040488	1.439933185894
H	2.899729157549	1.524437670565	1.318043116469	H	3.341297427283	1.641227610757	1.222443073540
C	5.401125507901	2.896854821921	-0.699007425880	C	5.533529878113	2.912408273481	-1.164789150223
H	5.082726136673	3.947068935992	-0.596290586733	H	5.270445627258	3.973544103526	-1.032300740652
H	5.776432495400	2.753845964906	-1.723579540495	H	5.733659779361	2.739985156560	-2.232548507126
H	6.249720822064	2.748781020431	-0.012041105474	H	6.475008223100	2.742392706616	-0.619226882148
C	-2.721208827771	-0.828792804972	3.070582713095	C	-2.712801718453	-0.89956656773 1	3.197244635883
C	-2.200684239592	-2.152799871763	3.139441524873	C	-2.081097545082	-2.155909401805	3.401673776454
C	-2.347362417426	-2.906347475346	4.313947124720	C	-2.429891983060	-2.939756796621	4.505008110112

H	-1.952694319934	-3.927034071140	4.336967198604	H	-1.942790554174	-3.911170633739	4.636745874842
C	-2.981963946440	-2.399650307784	5.450408770846	C	-3.380970802086	-2.528030992056	5.438361201999
C	-3.496596259501	-1.099322992767	5.378418202705	C	-4.005234279208	-1.297351621529	5.228271642271
H	-4.002957706688	-0.679210228427	6.253423874321	H	-4.761530368146	-0.956319729037	5.942389632051
C	-3.389394191070	-0.317151525277	4.222709798199	C	-3.700710504140	-0.488145518454	4.131806163212
C	-3.994774976268	1.070232553458	4.262326724898	C	-4.453094090676	0.808982127062	3.993679581239
H	-4.346207880182	1.311434936509	5.276422600244	H	-5.045003689937	1.013023615746	4.897114299942
H	-4.851796833467	1.163399822200	3.577722786527	H	-5.141253253868	0.788320251580	3.134813415531
H	-3.273470141998	1.847569775012	3.968708810797	H	-3.782913668855	1.664413985151	3.830623258326
C	-1.501650054924	-2.823415424422	1.974921539994	C	-1.054051382515	-2.717737601004	2.453197798287
H	-1.470038780801	-3.913489156862	2.120313477011	H	-0.894246302042	-3.787773006224	2.647004061257
H	-0.460532396289	-2.477130684260	1.866299856875	H	-0.080794025908	-2.213270044316	2.559083447748
H	-1.998765376833	-2.625786800164	1.013790400875	H	-1.350541590984	-2.603269013492	1.400503384580
C	-3.103260403776	-3.211500063743	6.716405562793	C	-3.706334246922	-3.366338683637	6.639947988821
H	-2.843993284629	-4.267504493639	6.548207901024	H	-3.516189141743	-4.433450936142	6.453398084103
H	-4.126239724763	-3.171709940513	7.124943597750	H	-4.758089450979	-3.250685123274	6.941378491566
H	-2.430192569251	-2.825473412960	7.501900476900	H	-3.086187190177	-3.069341941149	7.502788855416
C	-3.711980084363	0.935758396031	1.100015981728	C	-3.391933997351	0.862943899438	1.111768863854
C	-4.925036545017	0.342793405260	0.659822910024	C	-4.439720750972	0.240243278688	0.401457253990
C	-5.912163082978	1.133622973741	0.058753621117	C	-5.302156155490	1.014386574487	-0.378738583668
H	-6.837841902560	0.656841652063	-0.280192725005	H	-6.109940064695	0.519806313285	-0.928154512566
C	-5.751590452689	2.514158455244	-0.118492756316	C	-5.163732754118	2.401917330150	-0.472858010747
C	-4.558404755031	3.092038491045	0.326357242603	C	-4.129293100345	3.006974481383	0.244082995568
H	-4.415172861242	4.171400656261	0.212380200154	H	-4.012452621564	4.094602514332	0.196670629731
C	-3.539349186954	2.332054016976	0.920843042728	C	-3.240158252526	2.262915060538	1.025455181806
C	-2.286650776738	3.046970587498	1.384548341636	C	-2.146754549739	2.973938570897	1.778876669673
H	-2.427668338314	4.138082527660	1.370982852227	H	-2.304255320865	4.061922796642	1.778537095493
H	-1.420139928881	2.817093087315	0.744923667448	H	-1.155903449983	2.778831229874	1.339147217864
H	-1.998947022960	2.760037347281	2.408361773397	H	-2.088051552285	2.641420434803	2.827283153310
C	-5.179457132845	-1.141215131122	0.819546059445	C	-4.634938482663	-1.250376394596	0.477894784390
H	-6.126441130324	-1.434856966050	0.342773187496	H	-5.459605945055	-1.582901206373	-0.168359602928
H	-5.229241971337	-1.433570216011	1.880436473411	H	-4.853499721875	-1.572503891624	1.508304066161
H	-4.377440757045	-1.745989623950	0.365332473060	H	-3.726108439856	-1.792207773760	0.168917753573
C	-6.829340209992	3.339143118730	-0.779384313255	C	-6.084625058883	3.211669864976	-1.340333654224
H	-7.822285536367	3.122750237391	-0.352361912385	H	-7.103267844133	2.795656693381	-1.347470595576
H	-6.892355269944	3.122240158688	-1.860135403323	H	-5.732130641650	3.224178080740	-2.385777766844
H	-6.640094940806	4.417463971712	-0.668886471512	H	-6.144068505630	4.256907499610	-1.003197943499
B	1.033309490389	-0.168534162723	-0.129074058686	B	1.279919037542	-0.010840802831	0.177946009534
H	1.840582415933	-0.744360360447	0.560483681190	H	2.198598213118	-0.529711569708	0.768087689264
N	-1.332360154089	0.024028685323	1.075138612728	N	-1.061892284014	0.016844461594	1.427137383348
B	-2.572459738897	0.043340652961	1.756564837924	B	-2.370680586972	-0.011677556562	1.947074066113
C	-0.291469798746	-0.021805504764	0.482601120775	C	-0.010240715053	0.045320026083	0.858571105846

Table S2. Optimized cartesian coordinates of **5a**$^{\mathbf{N}i\mathbf{Pr2}}$ (left B3LYP/def2-SVP, right PBE0-D3/def2-SVP).

B	-0.166079335069	-0.800099769400	-0.276190026380	B	0.081989859186	-1.005011738830	-0.152126146413
H	0.701876686621	-1.288947147462	-0.965189453481	H	0.924326608912	-1.574894226392	-0.812496417249
C	0.309537254649	0.427118383369	0.399613339174	C	0.579800850138	0.197477431526	0.534862378351
N	0.844646257975	1.395637872598	0.847183543476	N	1.078152171588	1.174395659568	0.999289253861
B	1.527976682048	2.643275121566	1.167367331393	B	1.565788897721	2.531106724763	1.189784240813
N	1.138045769358	3.354413224766	2.368820129258	N	1.176396788656	3.215540216908	2.394819327306
C	1.023328923650	4.825974680952	2.361799738535	C	0.819962832586	4.632856219708	2.358169978146
H	1.425117862489	5.145045933283	1.391431986801	H	1.051275802198	4.965883304399	1.336783225220
N	2.565991783760	3.080391750613	0.268468126847	N	2.403768380297	3.090170329680	0.173543250182
C	0.709341177691	2.672747731445	3.608770993357	C	0.975330259919	2.521901774797	3.671023920340
H	0.686005035403	3.451236533106	4.387530238724	H	0.861033696609	3.312991860328	4.430285974194

C	2.662407975389	2.672145963054	-1.154523449162	C	2.402930146255	2.637083542790	-1.225601781821
H	3.432425351583	3.324805014556	-1.594354976888	H	3.000613150053	3.382205664020	-1.775714988715
C	1.843732550065	5.538210405412	3.452791733140	C	1.622861037131	5.508716158351	3.321642664645
H	1.431082957273	5.371658562594	4.460596013352	H	1.375969710688	5.297200869535	4.373830927694
H	1.835216812520	6.626472677981	3.280320559601	H	1.399148287006	6.572083789518	3.145612295229
H	2.892469418125	5.205453888423	3.462106723547	H	2.706007506232	5.363961577715	3.197241734217
C	-0.438448991144	5.309298899377	2.399155663643	C	-0.681105962105	4.854256642140	2.555734983682
H	-1.029092689316	4.839910787150	1.597648994114	H	-1.260554409041	4.254539822904	1.837880803637
H	-0.483415294575	6.402411903884	2.262966167315	H	-0.938414956266	5.914949514885	2.408720602376
H	-0.923608137479	5.080637733571	3.361719300775	H	-1.002451133050	4.576620417290	3.572157803812
C	1.745633562025	1.636431702665	4.067036796165	C	2.216243029322	1.721396513051	4.056430927416
H	1.837079841480	0.814908483292	3.339404133070	H	2.397622690540	0.913213925899	3.330796526828
H	1.454659963480	1.195032820312	5.034090239271	H	2.096840440627	1.260797714646	5.049366523223
H	2.737389987335	2.099703439327	4.185958827382	H	3.105654253355	2.368930584965	4.077620455732
C	-0.701313378952	2.062674245206	3.570153257094	C	-0.284347931955	1.659077486849	3.752585970293
H	-1.446023048815	2.784280262545	3.205801990757	H	-1.172218955279	2.206763994272	3.407849557795
H	-1.004426825551	1.744349392018	4.581529002480	H	-0.461760466394	1.342669722126	4.792725851746
H	-0.736999641784	1.180812619903	2.918937926678	H	-0.183377513493	0.754113160489	3.140385416298
C	3.151685614108	1.230866384865	-1.386367058240	C	3.085376332039	1.288441777584	-1.465400563794
H	4.069124965805	1.023572644157	-0.817679303621	H	4.080524692349	1.258005693554	-1.000937809670
H	3.379688276561	1.084494172918	-2.455248907794	H	3.210434512334	1.121689663666	-2.547010376069
H	2.402011595179	0.480362727347	-1.106254367421	H	2.500261624366	0.447688907852	-1.072474005289
C	1.371702686713	2.967259743393	-1.931838457345	C	1.004812203911	2.683623666758	-1.837473723263
H	0.536886662987	2.332057986226	-1.601718521770	H	0.338406623325	1.928104970766	-1.395480718497
H	1.523562777167	2.779409866092	-3.007065106688	H	1.053168744736	2.481595487103	-2.918701996878
H	1.074966952819	4.020616883958	-1.808045893290	H	0.553080534137	3.676438091883	-1.690910179212
C	3.692547617586	3.897630690022	0.765104119546	C	3.402405821843	4.105596573471	0.512137695742
H	3.466855050156	4.091786589489	1.820046512105	H	3.268125003456	4.296835256203	1.584829197397
C	5.033308346388	3.142264039299	0.748875457393	C	4.833461520131	3.594639806076	0.342301411003
H	4.952767595285	2.183916174645	1.284217728070	H	4.983604870646	2.661759266456	0.905710191265
H	5.811892967693	3.745780342942	1.243559717956	H	5.551829046470	4.342009083906	0.713847044682
H	5.383395645856	2.935061525542	-0.274830351984	H	5.078848440316	3.401095284164	-0.713786869833
C	3.836911012977	5.260412517460	0.064595697377	C	3.211328704485	5.431921682249	-0.224950015217
H	4.168974558703	5.158338862914	-0.980941060701	H	3.418576833123	5.337686260950	-1.302490460335
H	4.591564118914	5.874031924361	0.582573999021	H	3.902080970299	6.191640554026	0.172087220753
H	2.889668832615	5.820422829971	0.060277267138	H	2.185640640227	5.813564184725	-0.115226585045
C	-1.535326553079	-1.390744602636	-0.190661853576	C	-1.353036534727	-1.406634527335	-0.137652903810
N	-1.925307152869	-2.519068564775	-0.874673852830	N	-1.848539194291	-2.445521405144	-0.866805182634
C	-2.727829730188	-0.902120119243	0.644174028977	C	-2.505426541596	-0.797212223305	0.653111233477
C	-3.920502074379	-1.680191683627	0.034072721550	C	-3.745779038123	-1.370869571640	-0.058521826278
H	-4.688533686815	-1.926870881278	0.783020461521	H	-4.589667938075	-1.530860240283	0.629270071490
H	-4.405500676270	-1.053909040914	-0.731328878742	H	-4.081149807602	-0.653492188498	-0.824028132820
C	-3.348015564103	-2.942824342198	-0.642139781598	C	-3.311787438362	-2.668711937124	-0.752586632698
C	-3.449420514587	-4.187302162189	0.262098981293	C	-3.639584053026	-3.918661753203	0.069628351366
H	-2.990008871892	-5.061586999827	-0.220170296678	H	-3.233054094449	-4.816380907700	-0.417340711696
H	-4.509910802089	-4.425276132214	0.440322761437	H	-4.731106709234	-4.038491701642	0.140843294458
H	-2.970292620865	-4.039998933374	1.238456497092	H	-3.239039823705	-3.867377546634	1.089862901274
C	-4.093536468325	-3.262263287499	-1.946288277180	C	-3.978025648716	-2.823381726814	-2.117946040695
H	-4.134736476697	-2.395974037660	-2.618439559384	H	-3.844966877122	-1.927776283415	-2.737763197536
H	-5.129756998017	-3.549224024381	-1.707794206900	H	-5.058301761538	-2.981588335468	-1.981320385533
H	-3.629367531872	-4.102325559896	-2.484470593669	H	-3.574289543111	-3.689689641672	-2.663455107054
C	-2.966423369823	0.612988653026	0.521633773924	C	-2.530984037118	0.730876494745	0.609178786046
H	-3.894453632236	0.894414797594	1.048225906940	H	-3.454044727610	1.105787814712	1.081408438335
H	-3.063698664524	0.915841267028	-0.532144834157	H	-2.498227104003	1.095853610330	-0.428479231241
H	-2.144882053168	1.193277805973	0.962994206378	H	-1.680809979718	1.169563887066	1.147963866542
C	-2.523548872254	-1.259975106400	2.134586675154	C	-2.423071388698	-1.261342565605	2.115353744499
H	-1.650720079682	-0.733902005820	2.544972868459	H	-1.510435493119	-0.874864628277	2.589036432921
H	-2.354091709017	-2.335992492319	2.282498902239	H	-2.394193634210	-2.356884217682	2.197579444488
H	-3.410271146762	-0.971318378903	2.724319476592	H	-3.294149314977	-0.897277247346	2.684726744986
C	-1.037541051122	-3.255374970591	-1.739611347352	C	-1.013413178638	-3.273218281531	-1.675819757357

C	-0.223053812420	-4.293110529946	-1.212569924901	C	-0.392683522790	-4.409900098880	-1.112288032280
C	0.569873118173	-5.037701248738	-2.099182012311	C	0.362809588974	-5.238676702204	-1.948220912251
H	1.193093968304	-5.845329178128	-1.708078249585	H	0.841891561810	-6.126664512627	-1.528203409407
C	0.596301335579	-4.757253510311	-3.462016133005	C	0.536627079106	-4.939964741048	-3.293659642327
H	1.218208706259	-5.353444227955	-4.135083986270	H	1.127507254941	-5.602251743203	-3.931475501933
C	-0.150244395252	-3.691480245610	-3.956819370124	C	-0.012598051320	-3.775150646894	-3.815624872798
H	-0.091149967076	-3.446144655345	-5.019795383178	H	0.171227858575	-3.515903453052	-4.861322433606
C	-0.962700916709	-2.914960481829	-3.117660745223	C	-0.778464330751	-2.915197779794	-3.022639355677
C	-1.660578108003	-1.694460801595	-3.718181091634	C	-1.244803729381	-1.596167181778	-3.613451749633
H	-2.323214844898	-1.277227578498	-2.948205066095	H	-1.892370728251	-1.111620080312	-2.868523516772
C	-0.095460585107	-4.583887315195	0.282481689156	C	-0.424260582497	-4.705437014908	0.376335978469
H	-0.837465494295	-3.969556776283	0.806526233690	H	-1.127146761349	-3.999914964617	0.837839031156
C	1.288098366632	-4.150812176094	0.806979418200	C	0.948049753782	-4.430794038014	0.998602314541
H	2.096201248251	-4.736752828558	0.338523723015	H	1.715144862436	-5.104624881362	0.583687227766
H	1.350478278973	-4.307691444410	1.896863039519	H	0.913927309937	-4.589244506310	2.088518977829
H	1.470811594396	-3.086454951619	0.602840725310	H	1.259217917613	-3.393495019224	0.810830273752
C	-0.361884176126	-6.057790263689	0.636006717830	C	-0.889177386633	-6.125858169410	0.695299768567
H	-1.335032377096	-6.407580237608	0.259341238690	H	-1.864910551321	-6.354696443077	0.242001435094
H	-0.352565454102	-6.197149023538	1.729514987793	H	-0.979534889141	-6.264638550168	1.784188965669
H	0.411868405448	-6.722373427735	0.218656909216	H	-0.170834561754	-6.877326450650	0.331059042982
C	-2.515962041638	-2.035546157126	-4.951583351301	C	-2.041136038060	-1.775316865404	-4.906401143714
H	-1.893088609417	-2.358844197452	-5.801261118905	H	-1.407697898155	-2.167599446650	-5.717694674389
H	-3.079196709297	-1.147993146487	-5.283800296549	H	-2.442417691432	-0.808030108791	-5.247887986112
H	-3.238666788902	-2.839591245469	-4.747109699240	H	-2.883993474581	-2.470294666485	-4.780257192957
C	-0.637761646815	-0.594839401276	-4.066016831841	C	-0.053182938775	-0.660397606024	-3.839039917971
H	-0.056167686929	-0.303617524711	-3.180191594591	H	0.492818496446	-0.490865848655	-2.900817650109
H	-1.154145023884	0.301315459919	-4.448996354892	H	-0.398034120901	0.315387134490	-4.217726609607
H	0.065006129241	-0.934281108696	-4.845043572055	H	0.647657246011	-1.080535365179	-4.578647331389

References

[1] R. Jazzar, R. D. Dewhurst, J.-B. Bourg, B. Donnadieu, Y. Canac, G. Bertrand, *Angew. Chem. Int. Ed.* **2007**, *46*, 2899–2902; *Angew. Chem.* **2007**, *119*, 2957–2960.

[2] M. Arrowsmith, D. Auerhammer, R. Bertermann, H. Braunschweig, M. A. Celik, J. Erdmannsdörfer, I. Krummenacher, T. Kupfer, *Angew. Chem. Int. Ed.* **2017**, *56*, 11263–11421; *Angew. Chem.* **2017**, *129*, 11417–1421.

[3] J. J. Eisch, B. Shafii, J. D. Odom, A. L. Rheingold, *J. Am. Chem. Soc.* **1990**, *112*, 1847–1853.

[4] J. Haberecht, A. Krummland, F. Breher, B. Gebhardt, H. Rüegger, R. Nesper, H. Grützmacher, *Dalton Trans.* **2003**, 2126– 2132.

[5] G. Sheldrick, *Acta Cryst.* **2015**, *A71*, 3–8.

[6] G. Sheldrick, *Acta Cryst.* **2008**, *A64*, 112–122.

[7] A. L. Spek, *Acta Cryst*. **2015**, *C71*, 9–18.

[8] A. D. Becke, *J. Chem. Phys.* **1993**, *98*, 5648–5652.

[9] a) C. Adamo, V. Barone, *J. Chem. Phys.* **1999**, *110*, 6158–6169; b) S. Grimme, J. Antony, S. Ehrlich, H. Krieg, *J. Chem. Phys*. **2010**, *132*, 154104.

[10] S. Kozuch, J. M. L. Martin, *Phys. Chem. Chem. Phys.* **2011**, *13*, 20104–20107.

[11] M. J. Frisch, G. W. Trucks, H. B. Schlegel, G. E. Scuseria, M. A. Robb, J. R. Cheeseman, G. Scalmani, V. Barone, G. A. Petersson, H. Nakatsuji, X. Li, M. Caricato, A. V. Marenich, J. Bloino, B. G. Janesko, R. Gomperts, B. Mennucci, H. P. Hratchian, J. V. Ortiz, A. F. Izmaylov, J. L. Sonnenberg, D. Williams-Young, F. Ding, F. Lipparini, F. Egidi, J. Goings, B. Peng, A. Petrone, T. Henderson, D. Ranasinghe, V. G. Zakrzewski, J. Gao, N. Rega, G. Zheng, W. Liang, M. Hada, M. Ehara, K. Toyota, R. Fukuda, J. Hasegawa, M. Ishida, T. Nakajima, Y. Honda, O. Kitao, H. Nakai, T. Vreven, K. Throssell, J. A. Montgomery, Jr. J. E. Peralta, F. Ogliaro, M. J. Bearpark, J. J. Heyd, E. N. Brothers, K. N. Kudin, V. N. Staroverov, T. A. Keith, R. Kobayashi, J. Normand, K. Raghavachari, A. P. Rendell, J. C. Burant, S. S. Iyengar, J. Tomasi, M. Cossi, J. M. Millam, M. Klene, C. Adamo, R. Cammi, J. W. Ochterski, R. L. Martin, K. Morokuma, O. Farkas, J. B. Foresman, D. J. Fox, *Gaussian 16, Revision B.01*, Gaussian, Inc. Wallingford CT, **2016**.

[12] P. Flükiger, H. P. Lüthi, S. Portmann, J. Weber, *Molekel 4.0*, Swiss Center for Scientific Computing, Manno (Switzerland), **2000**.

[13] E. D. Glendening, J. K. Badenhoop, A. E. Reed, J. E. Carpenter, J. A. Bohmann, C. M. Morales, P. Karafiloglou, C. R. Landis, F. Weinhold, *NBO 7.0*, Theoretical Chemistry Institute, University of Wisconsin, Madison, WI, **2018**.
